# Qualitative and quantitative proteomic analyses of *Schistosoma japonicum* eggs and egg-derived secretory-excretory proteins

**DOI:** 10.1186/s13071-019-3403-1

**Published:** 2019-04-16

**Authors:** Carolina De Marco Verissimo, Jeremy Potriquet, Hong You, Donald P. McManus, Jason Mulvenna, Malcolm K. Jones

**Affiliations:** 1 School of Veterinary Science, The University of Queensland, Brisbane, QLD Australia; 2 QIMR Berghofer Medical Research Institute, Brisbane, QLD Australia; 3 Present Address: Medical Biological Centre, Queen’s University Belfast, Belfast, UK

**Keywords:** Eggs, ESP, Mass spectrometry *Schistosoma japonicum*, Schistosomiasis, SWATH analysis

## Abstract

**Background:**

Schistosome parasites lay up to a thousand eggs per day inside the veins of their mammalian hosts. The immature eggs deposited by females against endothelia of venules will embryonate within days. Approximately 30% of the eggs will migrate to the lumen of the intestine to continue the parasite life-cycle. Many eggs, however, are trapped in the liver and intestine causing the main pathology associated with schistosomiasis mansoni and japonica, the liver granulomatous response. Excretory-secretory egg proteins drive much of egg-induced pathogenesis of schistosomiasis mansoni, and *Schistosoma japonicum* induce a markedly distinct granulomatous response to that of *S. mansoni*.

**Methods:**

To explore the basis of variations in this responsiveness, we investigated the proteome of eggs of *S. japonicum*. Using mass spectrometry qualitative and quantitative (SWATH) analyses, we describe the protein composition of *S. japonicum* eggs secretory proteins (ESP), and the differential expression of proteins by fully mature and immature eggs, isolated from faeces and *ex vivo* adults.

**Results:**

Of 957 egg-related proteins identified, 95 were exclusively found in *S. japonicum* ESP which imply that they are accessible to host immune system effector elements. An *in-silico* analysis implies that ESP are able of stimulating the innate and adaptive immune system through several different pathways. While quantitative SWATH analysis revealed 124 proteins that are differentially expressed by mature and immature *S. japonicum* eggs, illuminating some important aspects of eggs biology and infection, we also show that mature eggs are more likely than immature eggs to stimulate host immune responses.

**Conclusions:**

Here we present a list of potential targets that can be used to develop better strategies to avoid severe morbidity during *S. japonicum* infection, as well as improving diagnosis, treatment and control of schistosomiasis japonica.

**Electronic supplementary material:**

The online version of this article (10.1186/s13071-019-3403-1) contains supplementary material, which is available to authorized users.

## Background

Schistosomiasis is a chronic parasitic disease affecting more than 250 million people in tropical and subtropical countries [1, 2]. Eggs of the three main *Schistosoma* species (*Schistosoma mansoni*, *S. haematobium* and *S. japonicum*) have been demonstrated to play a major role in human disease attributable to schistosomiasis, being involved in its morbidity, pathology, host immune modulation, diagnosis and transmission [3, 4]. A single female *S. japonicum* lays approximately 1000 eggs per day inside the veins of its mammal host [5]. Some 30% of those eggs will reach the faeces, whereas the remainder will be trapped in the liver and intestinal tissues, where they induce the main pathology of schistosomiasis, the host granulomatous response [6]. The presence of eggs in the tissues leads to a continuous antigenic stimulation that induces chronic inflammation, subsequent liver fibrosis and portal hypertension, which often evolve into conditions such as hepatosplenomegaly and ascites [2, 6, 7].

When first released into the host circulation, the eggs of *S. mansoni* and *S. japonicum* are immature and, therefore, smaller and less complex than mature eggs [8, 9]. Subsequent egg maturation takes seven days. During this process, the eggs develop an extra-embryonic envelope beneath the shell, the inner envelope, that is highly active metabolically and is thought to be the primary source of the egg’s secretions [8]. The shell has numerous pores through which these secretions escape, releasing in *S. mansoni* at least, highly immunogenic molecules such as Kappa-5, IL-4-inducing principle (IPSE/ alpha-1), and the T2 ribonuclease Omega-1 [10–12]. Over recent years, total and specific proteins from whole eggs, egg secretory proteins (ESP), and soluble egg antigens (SEA) have been implicated in processes such as modulation of the host immune response towards a *T helper* (*Th*2) pattern [6, 13, 14]. Moreover, specific egg-derived proteins, including Sm-P40 and thioredoxin peroxidase-1 (Sm-TPx-1), were shown to induce liver lesions and peri-ovular reactions [15, 16]. Other proteins, such as Sjp40 have shown potential to be used for early diagnosis of schistosomiasis [17].

Egg ESP provoke and maintain the granulomatous response of the host and, therefore, play an important role in pathogenesis. For the parasite, however, this response is adaptive, in that it pushes eggs across endothelial boundaries into the intestinal lumen for excretion from the host, an extremely important step for the parasite to continue its life-cycle, but one still poorly understood [18, 19]. Indeed, the stimulation of the host immune response generates a cascade of reactions that leads to the activation of molecules, such as Interleukins, surface-adhesion molecules of vascular endothelia (ICAM-1, E-selectin and VCAM-1) and plasma factors that appear to help the eggs cross the intestinal barrier by causing modifications of the tight junctions [19–22].

Of significant interest in relation to the host-schistosome parasite interaction is the nature of the proteins that are released from the eggs. Different proteomic analyses of the schistosome eggs and derived secretions have revealed their complexity and variability [23, 24]. Whereas Liu et al. [23] evaluated the whole *S. japonicum* egg and identified a set of 258 proteins in this type of sample, two distinct investigations of the ESP derived from *S. mansoni* showed that the eggs release as few as six proteins [25], or as many as 188 [26]. This wide discrepancy might be explained by the different proteomic methodologies applied in each investigation and indicate the necessity of a careful comparison between studies.

Nonetheless, a more specific proteomic approach of the *S. japonicum* eggs could result in a better understanding of its biology, as well as the severe pathology of schistosomiasis associated with the granulomatous response and the host-parasite interactions. Moreover, these data could lead to improvement of the diagnosis of schistosomiasis, which is currently primarily based on direct or indirect detection of eggs and their quantification [27–29]. To address these questions, we investigated the protein composition of ESP derived from *S. japonicum* eggs, as well as the differential expression of proteins in fully mature and immature eggs through mass spectrometry qualitative and quantitative (SWATH) analyses. Through these approaches we explored the potential role of different proteins in the host-parasite relationship, immune modulation and pathogenesis of schistosomiasis japonica and generated a list of suitable molecular targets with potential for diagnosis and treatment of this disease.

## Methods

### Purification of *S. japonicum* eggs

ARC Swiss mice were infected percutaneously with 40 cercariae of *S. japonicum* (Anhui strain, China) shed from field isolates of *Oncomelania hupensis* snails. After 6 weeks, feces were collected and the mice were perfused to collect the livers and worms.

Embryonated *S. japonicum* eggs from mice faeces (herein designated mSj eggs for mature *S. japonicum* eggs) were purified from samples of at least 15 infected animals by serial passage through meshes of 500, 150 and 45 µm, respectively. The eggs retained in the last sieve were submitted to a 60% iodixanol (w/v) (OptiPrep, Sigma, Gillingham, UK) density gradient and centrifuged at 2000× *rpm* for 2 min. The layer containing the purified eggs in feces was verified by optical microscopy in order to ensure that debris were removed (Additional file 1: Figure S1). Liver eggs were obtained by Collagenase B (Sigma) digestion, according to the method of Dalton et al. [30], and were further purified using 60% iodixanol density gradient and verified by optical microscopy, as described above.

After perfusion, the *S. japonicum* worms were cultured in Sterile RPMI 1640 Cell Culture Media (Gibco, Dublin, Ie), supplemented with 10 µl/ml of penicillin-streptomycin (10,000 U/ml, Gibco), for 24 h at 37 °C, 5% CO_2_. Immature eggs (iSj eggs) were then collected within the whole culture media, washed three times with sterile phosphate buffer solution (PBS 1×), verified by optical microscopy (Additional file 2: Figure S2) and frozen at -20 °C. All the procedures for isolation of liver eggs and iSj eggs were repeated at least three times and each time involved a group of three animals. For further analyses, all samples from mSj eggs, liver eggs or iSj eggs were pooled together.

### Egg secretory proteins (ESP)

To obtain ESP, approximately 500 eggs purified from liver were added per well in a 96-well plate with sterile RPMI 1640 media (Gibco), supplemented with 10 µl/ml of penicillin-streptomycin (10,000 U/ml, Gibco), and incubated at 37 °C, 5% CO_2_. The supernatant was collected each hour in the first 3 h incubation. To determine whether eggs hatched and if eggs were viable post-incubation, the percentage of hatched eggs were observed under microscopy before and after incubation. The viability of the eggs after incubation was verified by making the eggs hatch (Additional file 3: Figure S3). Supernatants collected in the first 3 h were then centrifuged at 4 °C, 14,000× *g* for 30 min and the supernatant was frozen at -20 °C. These procedures were repeated at least three times (eggs obtained from different group of animals).

### Protein extraction and fractionation

In order to extract the total proteins from mSj, iSj, and liver eggs, eggs were ruptured by freezing in liquid nitrogen, after which lysis buffer was added (1% SDS, 10 mM CHAPS, 0.5 M MgCl_2_, and protease inhibitor cocktail 1× in 100 mM TEAB). The samples were agitated at 4 °C for 40 min, centrifuged at 14,000× *g* for 10 min, and the supernatant was collected and frozen at -20 °C. The pellets were verified under an optical microscope to verify the rupture of the eggs. The proteins were concentrated and the buffer was exchanged using Millipore Amicon filters 10K. The protein concentration of each sample was verified using the Bicinchoninic acid assay (Pierce, Thermo Fisher Scientific, Paisley, UK) following the manufacturer’s protocol.

### Proteomic analysis: in-gel fractionation

Triplicate samples (20 µg) of proteins extracted from *S. japonicum* liver eggs and ESP, were resolved in 12% one-dimension (1DE) SDS-PAGE gels. After gel fixation, the proteins were stained with EZBlue G-250 colloidal Coommassie Stain (Sigma), and each line was fractionated into 12 gel bands. In-gel trypsin digestion was performed [31], and the mixture of peptides was then collected, dried in speed vacuum and frozen at -20 °C.

### Proteomic analysis: filter-aided sample preparation method (FASP)

The total proteins from mSj and iSj were processed by the FASP method [32]. Briefly, triplicate samples of 30 µg of protein were reduced (final concentration of 20 mM DTT, at 95 °C, 10 min) and alkylated (final concentration of 40 mM iodoacetamide, at room temperature for 30 min in the dark). The proteins were then transferred into a 10K Millipore Amicon filter (Merck Millipore, Hertfordshire, UK) and washed first with 8 M urea in 100 mM TEAB and then 50 nM TEAB. Trypsin was added into the samples (ratio 1:50) and tryptic digestion was performed at 37 °C for 18 h. The peptides were recovered in 50 mM TEAB, dried in speed vacuum, and frozen at -20 °C.

### Mass spectrometry (MS) analyses: information-dependent acquisition (IDA)

The peptide fractions from the *S. japonicum* liver eggs and ESP were suspended in 0.1% TFA and desalted on ZipTip C18 pipette tips, 5 µg (Millipore). The C18 tips were activated with 70% ACN/ 0.1% TFA and then equilibrated with 0.1% TFA before loading the peptides. Loaded peptides were washed with 0.1% TFA and eluted with 80% ACN/ 0.1% TFA before drying in speed vacuum, resuspended in 20 µl of 0.2% ACN/ 0.1% TFA complemented with iRT calibrant (Biognosys, Zurich, Switzerland) [33], and analyzed by reverse phase chromatography with an Eksigent nanoflex cHiPLC coupled to a TripleTOF 5600 mass spectrometer (ABSCIEX, Canada) equipped with a nano-electrospray ion source. The peptides were separated on the eksigent analytical cHiPLC column (3 μm, ChromXP C18CL, 120 Å, 15 cm × 200 µm) with a method using three consecutive linear gradients: 5–10% solvent B (acetonitrile/0.1% formic acid) over 2 min; 10–40% solvent B over 58 min; 40–50% solvent B over 5 min, at a 500 nl/min flow rate. Eluted peptides were acquired in positive mode by electrospray (voltage 2300 V) using IDA method, and those ions exceeding a threshold of 50 counts and possessing a charge state of +2 to +4, were set to trigger the acquisition of product ion spectra for the 10 the most intense ions with 10 second exclusion after one occurrence.

### MS: Sequential window acquisition of all theoretical fragment ion spectra (SWATH)

Peptides from mSj and iSj samples (triplicates), obtained from FASP were resuspended in 0.1% TFA, normalized and desalted on ZipTip C18 pipette tips, 5 µg (Millipore) as described above, dried in speed vacuum and resuspended in 30 µl of 2% ACN/0.1% FA complemented by iRT calibrant (Biognosys, Zurich, Switzerland) [33]. The peptides were separated on an Eksigent analytical cHiPLC column (3 m, ChromXP C18CL, 120 Å, 15 cm × 200 µm) using three linear gradients: 5–10% solvent B (ACN/0.1% formic acid) over 2 min, 10–40% solvent B over 58 min and 40–50% solvent B over 5 min at a 300 nl/min flow rate, and then directly introduced into a TripleTOF 5600 mass spectrometer (ABSCIEX, Ontario, Canada), as previously described. A rolling collision energy method was used to fragment all ions in a set of 26 sequential overlapping windows of 25 AMU over a mass range coverage of 350–1000 (m/z). An accumulation time of 100 ms was used for each fragment ion scan resulting in a total cycle time of 2.9 s. Data were acquired and processed using Analyst TF 1.7 software (AB SCIEX).

### Data analysis

The detected protein threshold was set as 0.1 and the false-discovery rate (FDR) was calculated using searches against a decoy database comprised of reversed sequences. All searches were conducted against *Schistosoma* sp. specific protein databases downloaded from WormBase Parasite (http://www.ebi.ac.uk), UniProt (http://www.uniprot.org), and Scientific Data Sharing Information Bioinformation (http://lifecenter.sgst.cn/protein). The set comprised a total of 39,768 non-redundant protein sequences.

X! Tandem Jackhammer TPP (2013.06.15.1) was used to search all IDA files (in triplicate for each sample) against a target/decoy version of the *Schistosoma* databases reference proteome set additionally containing a reversed version of each sequence and the iRT sequences. The spectral library was generated accordingly following Schubert et al. [62]; results were assessed for statistical validity in the Trans Proteomic Pipeline (TPP) using PeptideProphet and iProphet, and the false discovery rate was estimated using Mayu. SpectraST (version 5.0) in the TPP was used to normalize the retention time and generate a consensus library, while the OpenSWATH was used to build a target/decoy spectral library [33]. The final lists of proteins were generated based on the results of the triplicates, and included all proteins identified with at least two unique peptides.

For SWATH analysis, ProteoWizard msconvert was used to convert the files to mzML format, and then searches against the spectral library were performed using OpenSWATH with a FDR < 0.1%. Feature alignment was performed using the TRansition of Identification Confidence (TRIC) algorithm (https://pypi.python.org/pypi/msproteomicstools). Next, the R package *SWATH2stats* was used to filter low scoring peak groups and remove proteins with less than two associated peptides [34]. Then, the data were normalized (‘equalizeMedians’ method), summarized (Tukey’s median polish’ parameter estimation method), and finally differential expression analysis was performed with MSstats [35]. Differential expression values with an adjusted *P*-value ≤ 0.05 were considered significant.

The mass spectrometry proteomics data have been deposited to the ProteomeXchange Consortium *via* the PRIDE partner repository with the dataset identifier PXD012835, available at http://www.proteomexchange.org/submission/index.html.

### Bioinformatics analysis

The gene ontology and enrichment analyses were made using FunRich (Version 3.1.3) (http://funrich.org/download) and the gene ontology (GO) database (http://www.geneontology.org/). Pathways analysis was performed using the Reactome database (Version 3.5) (https://reactome.org/PathwayBrowser/) and SecretomeP 2.0 (http://www.cbs.dtu.dk/services/SecretomeP/), with mammalian parameters, was used to determine whether the proteins identified were predicted to be secreted through classical or non-classical pathways.

## Results

### Processing effects on egg viability

Matheson & Wilson [25] suggested that purification of schistosome eggs using the Dalton et al. method [30] could lead to their rupture and death. However, at the end of incubation, the eggs were verified as alive by hatching experiments, conducted in de-ionized water, which revealed hatching rates of over 90%, indicating that our methods of purification of liver eggs did not adversely affect their integrity or viability.

### Library: total proteins from *S. japonicum* eggs

To improve the sensitivity of the LC-MS/MS analysis, we fractionated the total proteins extracted from whole *S. japonicum* eggs purified from livers by resolving them in 1-DE SDS-PAGE gels. Of the total proteins identified using IDA analysis, excluding repeats and redundancies, we identified 862 proteins, filtered to 95% sequence identity, with a unique peptide count ≥ 2 (Additional file 4: Table S1). This list of proteins was used as our reference library for SWATH analysis. SecretomeP analysis (SecP) revealed that 524 (61%) of the proteins found in *S. japonicum* eggs contained a predicted signal or a non-classical sequence (Additional file 4: Table S1).

### Proteins found exclusively in the ESP

A total of 461 proteins were identified in the analysis of *S. japonicum* ESP. Of these, some 62% were predicted to contain classical or non-classical secretory sequences (Additional file 4: Table S1). In order to distinguish those proteins primarily secreted, the set of proteins identified in ESP was compared with the set of proteins identified in the total extract of whole *S. japonicum* eggs (Additional file 4: Table S1); those proteins found in both extracts were separated from proteins only present in ESP, resulting in a list of 95 proteins in ESP (Table 1).

**Table 1:** List of proteins identified exclusively in ESP from *Schistosoma japonicum* eggs

Accession	Protein name	Unused	% Cov	Peptides (95%)	SecP
tr|Q1HDV2	Fructose-bisphosphate aldolase	66.55	88.98	116	–
tr|B2LXU1	Enolase	0.20	65.89	47	–
tr|C1LRC5	Carbonyl reductase 1	0.00	81.95	45	–
tr|Q5DFP8	Putative uncharacterized protein	2.00	75.83	43	–
tr|C1LKA0	Purine nucleoside phosphorylase	2.17	72.13	40	NC
tr|G4VHK8	Tubulin beta chain	40.95	6.24	33	NC
tr|C1LIX7	Loss of heterozygosity 11 chromosomal region 2 gene A protein homolog	31.78	45.39	23	NC
tr|C1LIA3	Dihydrolipoyl dehydrogenase	36.56	57.74	22	NC
tr|C1LAW4	Major egg antigen (P40)	19.2	48.01	22	–
tr|A0A183N4E4	Uncharacterized protein	0.00	43.66	19	SP
tr|Q5DBS1	SJCHGC07012 protein	29.51	51.88	18	NC
tr|C1LNQ7	Protein disulfide-isomerase	27.32	43.20	17	SP
tr|Q5DEE6	Peptidyl-prolyl cis-trans isomerase	10.54	55.48	15	–
tr|G4V9B9	Putative heat-shock protein	0.03	26.78	15	–
tr|C1LS07	Cell wall integrity and stress response component 1	0.00	51.16	15	–
tr|C1L675	Arginase OS=Schis	25.96	52.20	14	–
tr|C1LWQ9	Elongation factor 1-alpha	17.02	64.45	14	–
tr|A0A094ZS46	Spectrin alpha chain	25.99	33.16	12	–
tr|C7TYX3	Putative uncharacterized protein	0.00	4.30	12	SP
tr|A0A183QGT0	Tubulin alpha chain	0.00	29.92	11	–
tr|G4VSJ5	Putative glycogen phosphorylase	0.00	37.74	11	–
tr|Q7Z0T1	Phosphoglycerate kinase	15.49	49.75	9	–
tr|C1LLK8	Peptidase M8, leishmanolysin,domain-containing protein	9.56	33.91	9	SP
tr|C7TQR6	Ribonuclease X25	8.96	53.29	9	SP
tr|Q5DHF8	Histone H3	5.64	41.17	9	NC
tr|C1L4U6	3’(2’), 5’-bisphosphate nucleotidase	16.83	36.62	8	SP
tr|C1LYD5	Ubiquitin C	7.32	7.80	8	–
tr|C1LQN5	Uncharacterized protein	0.08	44.90	8	SP
tr|A0A094ZSL4	Spectrin beta chain	13.2	21.92	7	–
tr|C1L6J2	Uncharacterized protein	12.2	47.83	7	SP
tr|Q86DZ2	Clone ZZZ395 mRNA sequence	10.59	39.91	7	–
tr|C1LEJ4	Histone H2A	5.28	56.80	7	NC
tr|C1LUP9	Peptidase inhibitor 16	0.00	46.02	7	SP
tr|C1L9E0	Receptor expression-enhancing protein	8.30	35.44	6	NC
tr|G4VS58	Putative prohibitin	7.97	34.02	6	NC
tr|A0A183L8B7	Uncharacterized protein	1.70	35.49	6	NC
tr|G4VG96	Cell polarity protein	10.72	18.52	5	–
tr|Q5DAE9	SJCHGC09095 protein	8.60	43.95	5	–
tr|C1LRF9	Fatty acid binding protein 7, brain	8.27	80.29	5	–
tr|Q7JNB4	Aspartic protease (Fragment)	6.14	27.34	5	SP
tr|A0A183MKN4	Uncharacterized protein	5.08	3.04	5	–
tr|Q5DF47	Putative uncharacterized protein	8.77	41.81	4	–
tr|A0A183NI42	Uncharacterized protein	7.00	31.92	4	–
tr|G4VFV4	Putative rap1	4.97	30.97	4	NC
tr|A0A183PY58	Uncharacterized protein	4.02	15.36	4	NC
tr|A0A183NZF1	Peptidyl-prolyl cis-trans isomerase	0.07	30.52	4	SP
tr|C1LID9	LAMA-like protein 2	6.72	1.90	3	SP
tr|C1LA47	Cathepsin B-like cysteine proteinase	6.18	25.15	3	SP
tr|Q5C774	SJCHGC02069 protein (Fragment)	5.38	3.84	3	SP
tr|Q5D9E0	SJCHGC01621 protein	4.86	2.87	3	NC
tr|C1LCS9	Phosphatase 2A inhibitor I2PP2A	4.86	52.49	3	–
tr|C1LKV5	Rho GDP-dissociation inhibitor 2	4.85	45.73	3	–
tr|Q5DBC3	SJCHGC06819 protein	4.57	17.52	3	NC
tr|Q5DFY2	SJCHGC06929 protein	4.40	25.61	3	SP
tr|Q5DFR4	ATP:ADP antiporter	4.01	40.83	3	–
tr|A0A183QZZ1	Uncharacterized protein	4.00	19.41	3	NC
tr|Q8I8A1	Rho2 GTPase (Fragment)	3.82	32.80	3	NC
tr|Q03528	22.6kd tegumental associated antigen	3.75	35.60	3	NC
tr|G4VDJ6	Putative adp-ribosylation factor, arf	0.06	45.86	3	NC
tr|A0A183NLB0	T-complex protein 1 subunit gamma	0.03	24.59	3	–
tr|C1LNJ0	Putative Retinol dehydrogenase 11	4.92	39.01	2	NC
tr|A0A183K1U2	Uncharacterized protein	4.81	19.40	2	SP
tr|A0A183JQD4	Uncharacterized protein	4.48	31.16	2	–
tr|C7TUC3	Putative uncharacterized protein	4.05	71.74	2	SP
tr|G4V9G5	Putative 60s ribosomal protein L12	4.03	45.44	2	NC
tr|C1L7G5	Ferritin	4.00	31.97	2	–
tr|Q5BT38	SJCHGC02867 protein (Fragment)	3.73	21.87	2	–
tr|C1LTL1	Universal stress protein	3.65	35.22	2	–
tr|C1LKU6	Erythrocyte band 7 integral membrane protein	3.24	15.64	2	NC
tr|C1LNJ7	Egg protein CP1531	3.18	17.52	2	SP
tr|G4V7S8	Serine hydroxymethyltransferase	3.09	69.43	2	–
tr|C1L857	Chaperonin containing TCP1, subunit 5 (Epsilon)	2.98	35.98	2	–
tr|A0A094ZXB9	Aminoacylase-1	2.89	17.02	2	–
tr|Q9B8Z2	Cytochrome *c* oxidase subunit 2	2.82	84.16	2	SP
tr|G4VJ08	Acyl-CoA thioesterase-related	2.47	10.39	2	NC
tr|C1LEX9	Transmembrane protease, serine 6	2.21	13.34	2	SP
tr|G4M1G4	REVERSED Dynein heavy chain, putative	2.14	57.13	2	–
tr|A0A183P199	Uncharacterized protein	2.06	13.37	2	NC
tr|A0A0R4I956	Myosin 2 heavy chain	2.05	15.50	2	–
tr|G4VB27	Putative uncharacterized protein	2.02	95.39	2	–
tr|G4V6J0	Putative copine	2.02	56.00	2	NC
tr|A0A094ZYC9	Dolichyl-diphosphooligosaccharide–protein glycosyltransferase subunit 2	2.01	9.40	2	SP
tr|C1LP66	Aquaporin-3 (AQP-3)	2.00	15.24	2	–
tr|A0A183LXE3	Uncharacterized protein	2.00	32.67	2	NC
tr|G4LXF1	Rab6, putative	1.87	33.98	2	NC
tr|A0A183KN75	Uncharacterized protein	1.68	17.46	2	NC
tr|Q7Z1I7	GTP-binding protein-like protein	1.37	14.06	2	–
tr|A0A183NZ41	Uncharacterized protein	1.37	79.14	2	–
tr|Q5DI05	Elongation factor Tu	1.24	16.21	2	SP
tr|A0A183MXQ1	Uncharacterized protein	1.24	27.05	2	NC
tr|A0A183QCJ7	Uncharacterized protein	1.16	95.11	2	NI
tr|A0A183LI65	Uncharacterized protein	0.77	19.61	2	–
tr|Q7Z1I5	40S ribosomal protein S8	0.64	23.57	2	–
tr|G4VQJ4	Putative adenine phosphoribosyltransferase	0.09	96.77	2	–
tr|A0A095ACH1	REVERSED Uncharacterized protein (Fragment)	0.00	24.43	2	NC

*Abbreviations*: ESP, egg secretory proteins; Unused, quantification for proteins in the ProteinPilot software; %Cov, ratio of the protein sequence covered by the matched peptides; Peptides (95%) total number of detected peptides with 95% of confidence; SecP, (Secretome P 2.0) results described as: SP, indicate presence of predicted signal sequence; NC, indicate non-classical secreted proteins; (–), non-detected

**Fig. 1:**
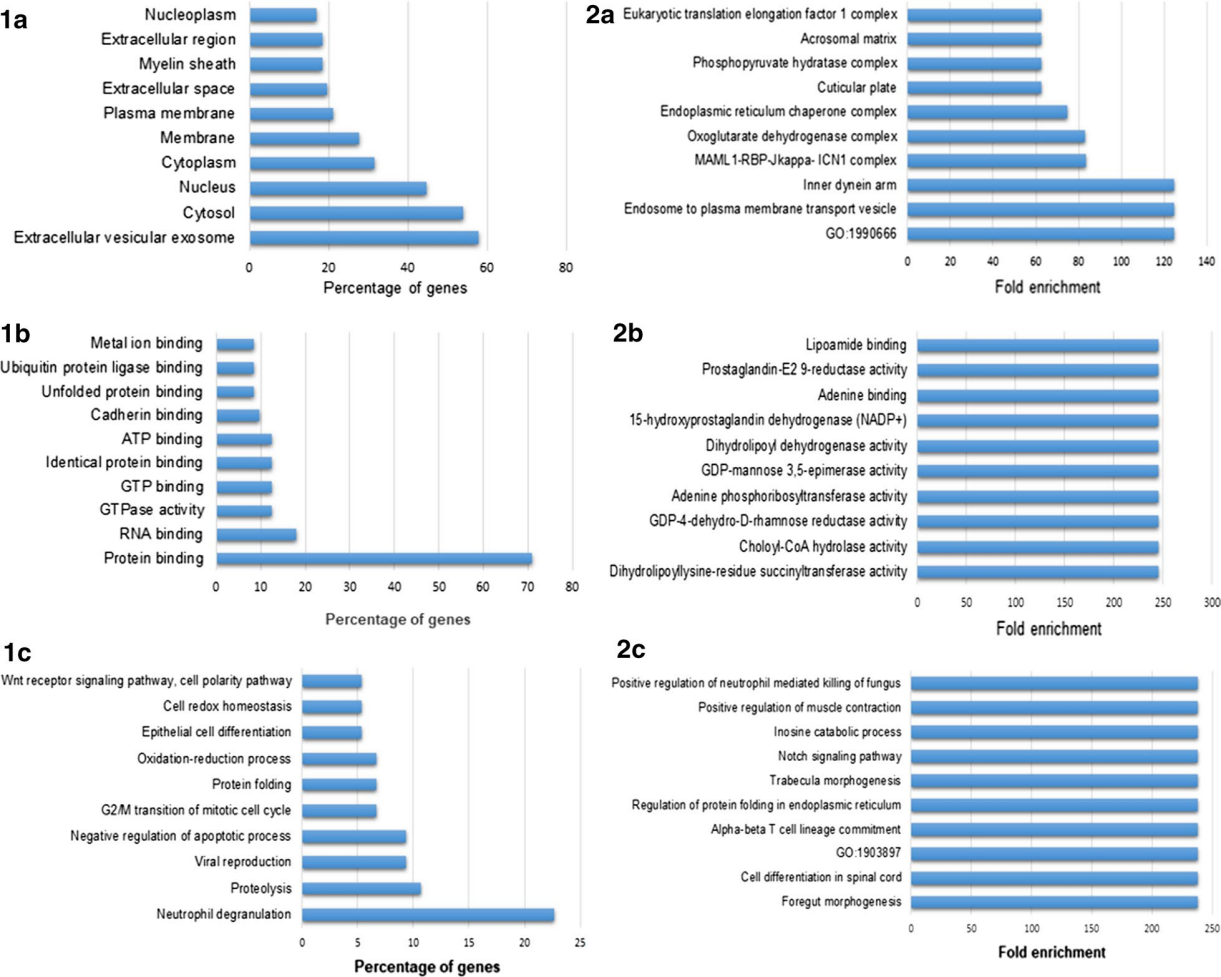
Gene ontology and enrichment analyses of the *S. japonicum* ESP. The panels show the top 10 classes of: Cellular component and related enrichment analysis (**1a** and **2a**); Molecular function and related enrichment analysis (**1b** and **2b**); Biological process and related enrichment analysis (**1c** and **2c**). Analyses performed using FunRich (V 3.1.3)

The complete gene ontology and enrichment analyses of the 95 proteins primary present in ESP is shown in Fig. 1 that presents the ten predominant cellular components, molecular functions and biological processes in which the proteins were classified. The analysis, based on homology of the *S. japonicum* proteins with human proteins, indicated that proteins present in the ESP were those commonly associated with extracellular vesicles, which is ultimately in line with the verification of the presence of classical or non-classical secretory sequences in 53 (54%) of these proteins (Table 1). Most of these proteins contained non-classical signal sequence that could be associated with protein secreted inside vesicles. In relation to the molecular function, most proteins were verified to be involved in “binding” and “catalytic activity”, while in terms of biological processes, the proteins in ESP were primary associated with neutrophil degranulation and proteolysis (Fig. 1).

### Proteomic comparison between immature (iSj) and mature (mSj) eggs

SWATH/MS is a type of data-independent acquisition method of analysis used to evaluate quantitatively complex samples with high reproducibility [36]. Using this method, we compared the expression level of each protein identified in immature (iSj) and mature (mSj) eggs. Our spectra libraries were constructed through analyses of the whole *S. japonicum* eggs from liver and comprised 862 identified proteins (Additional file 4: Table S1).

**Fig. 2:**
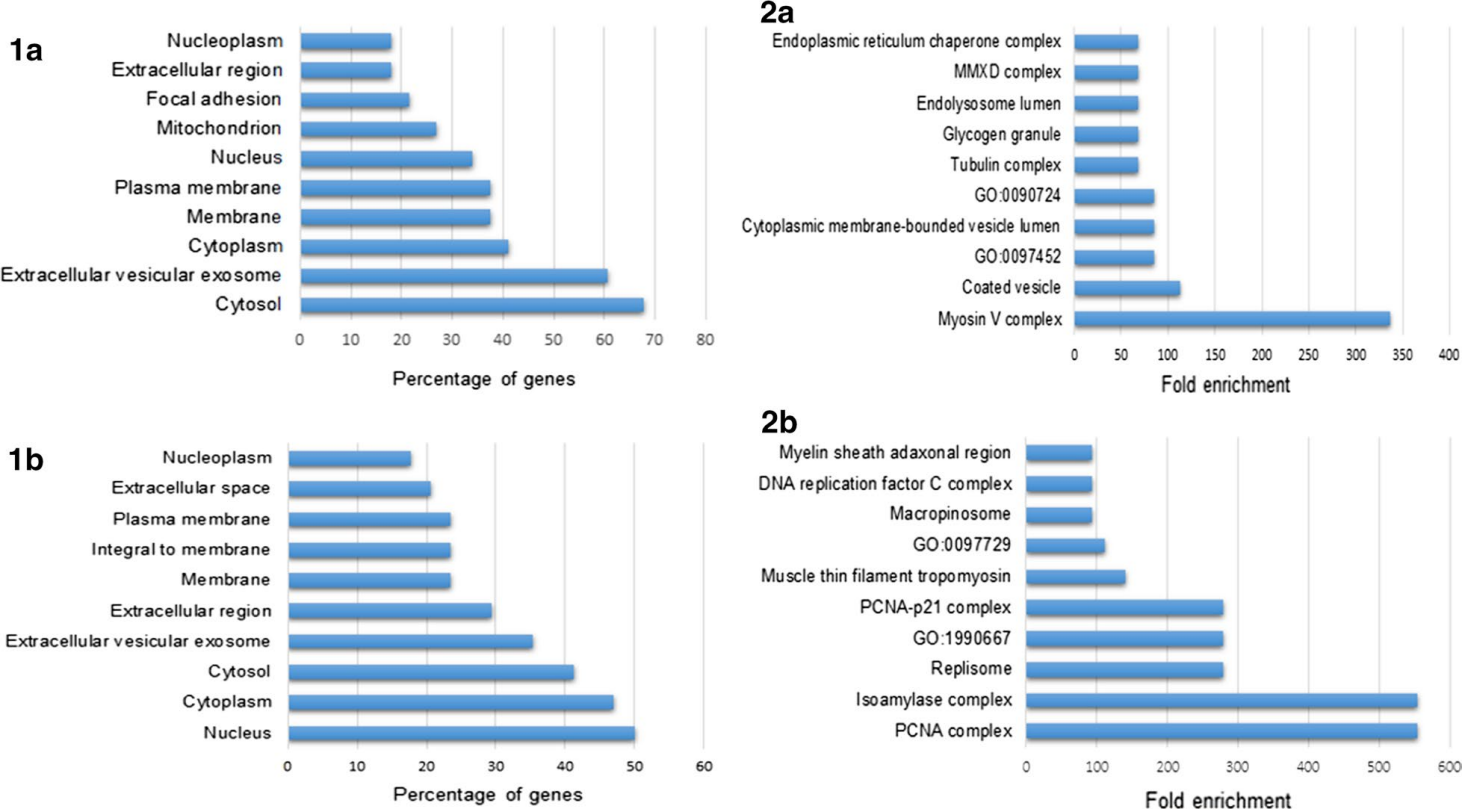
Cellular components and enrichment analyses of proteins that are up- and downregulated in *S. japonicum* immature eggs when compared to mature ones. The top 10 classes of cellular components and related enrichment analysis of proteins upregulated (**1a** and **2a**) and proteins down-regulated (**1b** and **2b**) in iSj. Analyses performed using FunRich (V 3.1.3)

**Fig. 3:**
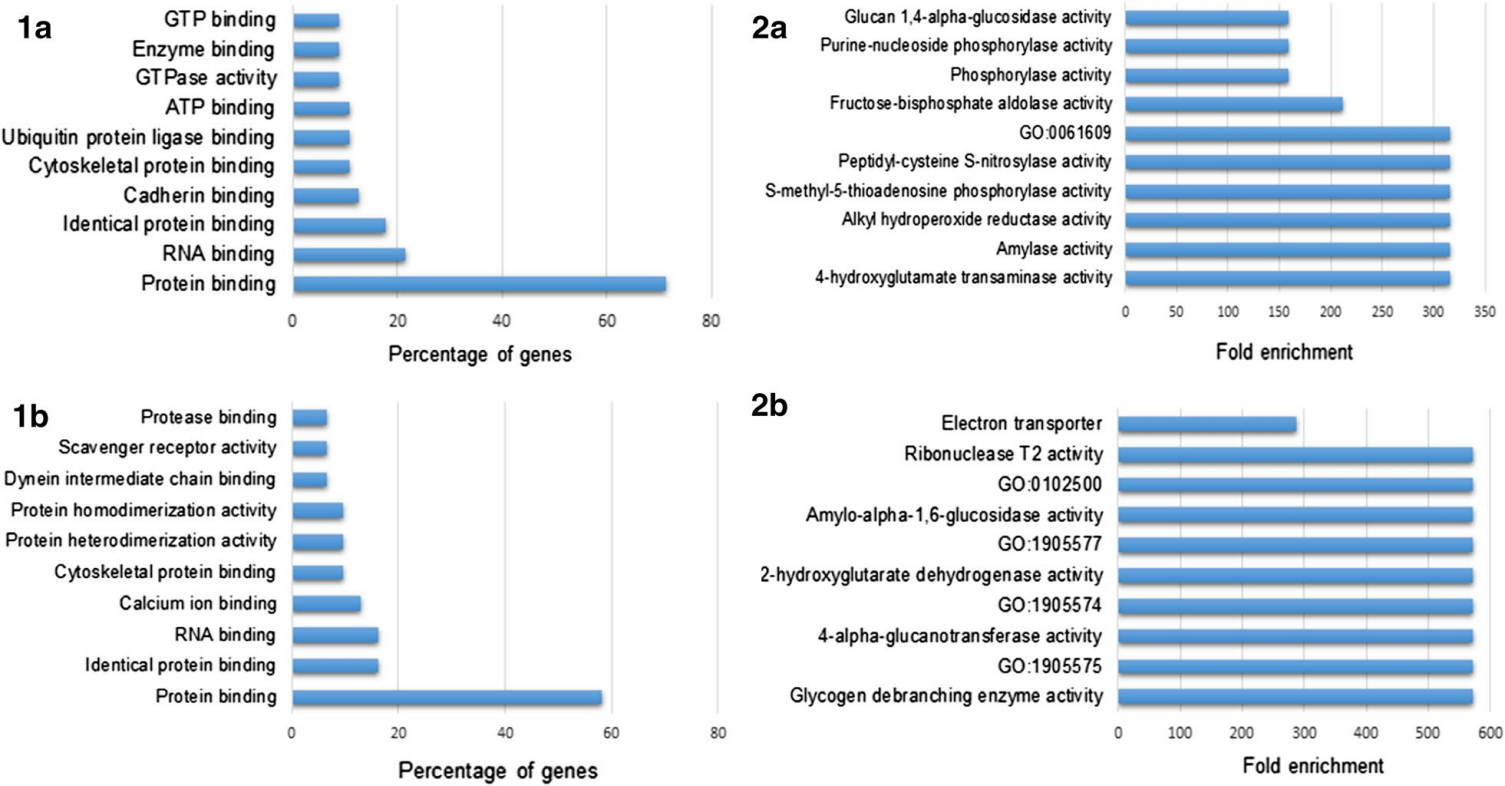
Molecular functions and enrichment analyses of proteins up- and downregulated in *S. japonicum* immature eggs when compared to mature ones. The top 10 classes of molecular functions and related enrichment analysis of proteins upregulated (**1a** and **2a**) and proteins downregulated (**1b** and **2b**) in iSj. Analyses performed using FunRich (V 3.1.3)

**Fig. 4:**
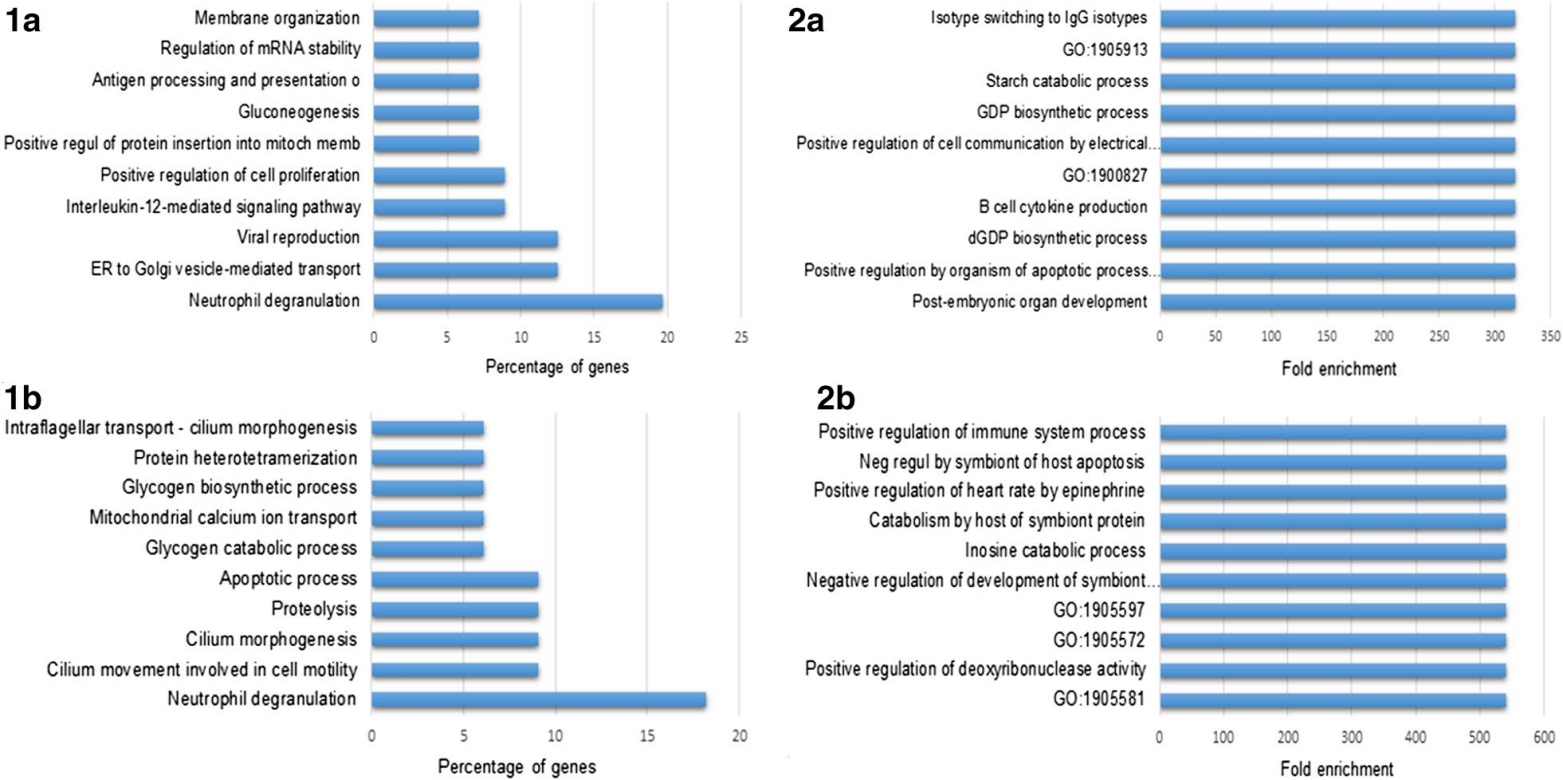
Biological processes and enrichment analyses of proteins up- and downregulated in *S. japonicum* immature eggs when compared to mature ones. The top 10 classes of biological processes and related enrichment analysis of proteins upregulated (**1a** and **2a**) and proteins downregulated (**1b** and **2b**) in iSj. Analyses performed using FunRich (V 3.1.3)

The relative expression levels of proteins were calculated using OpenSWATH and MStats and revealed 124 proteins expressed differentially between the two samples. Of these, 74 were shown to be upregulated, while 50 were downregulated proteins in iSj eggs when compared with mSj ones (Additional file 5: Table S2). According to gene ontology analysis (Figs. 2, 3 and 4), both up- and downregulated proteins in iSj are associated with a range of intracellular organelles and the cytosol. The enrichment analysis demonstrated that, in general, the proteins of iSj are components of organelles and structures participating in cell organization. Analysis of proteins predominantly expressed by mSj eggs showed they are associated with structures responsible for motility and the uptake of molecules (Fig. 2).

In relation to molecular functions, proteins up- and downregulated in eggs were classified with similar functions mainly related to binding and catalytic activity (Figs. 1a, b, 3). The enrichment analysis of iSj eggs revealed that the upregulated proteins are mostly involved in protein and energy production, while proteins predominantly in mSj eggs are associated with cell signaling, movement, transport and regulatory functions (Figs. 2a, b, 3).

Gene ontology analysis also demonstrated that both iSj and mSj eggs seem to be capable of stimulating the host immune system, since their proteins were predicted to be mainly involved in the process of neutrophil degranulation. On the other hand, the other main processes associated with each egg stage indicated that iSj and mSj eggs are performing quite different processes (Fig. 4). While proteins of immature eggs were primary involved in cell proliferation, those of mature eggs were mostly associated with energetic metabolism, especially glycogen degradation, movement and proteolytic activities.

**Fig. 5:**
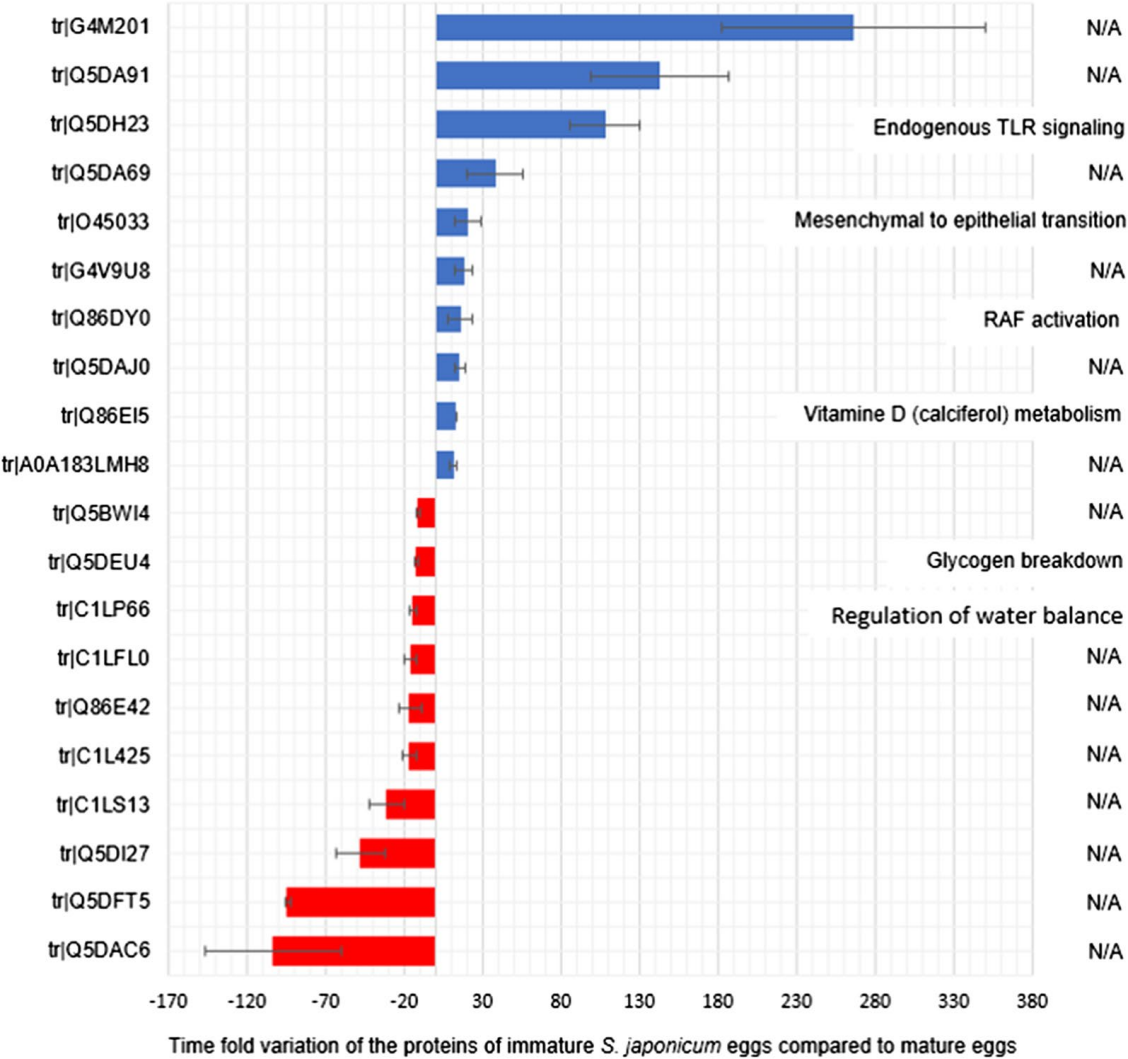
Top 10 proteins that are differentially expressed in immature and mature *S. japonicum* eggs. Proteins analyzed by SWATH method. Results presented as average ± standard deviation of the negative (red) or positive (blue) variation of the proteins of *S. japonicum* immature eggs compared with the mature eggs. The bars on the left, accession number of the proteins. The text of the right, the respective predicted biological pathway in which the protein is involved. Biological pathway analyses performed using FunRich (V 3.1.3)

The 10 most significantly up- and downregulated proteins and the respective biological pathways in which they were predicted to be involved are shown in Fig. 5. Considering proteins previously characterized, the stromal cell-derived factor 2-like (G4M201) was the most significantly abundant protein in iSj eggs, while the most abundant protein in mSj eggs was the cell wall integrity and stress response component 1 protein. Many proteins could not be classified according to their biological pathway, but those that were indicated that iSj eggs prioritize pathways related to development, organization and immunomodulation. On the other hand, mSj eggs seemed to be expressing proteins with roles in energy production and regulation of water (Fig. 5), which could be related to the presence of a highly active miracidium and the environmental conditions that mature eggs have to face.

### Proteins from ESP, immature and mature eggs stimulate the host immune system in different ways

Reactome analysis, concentrating only on those pathways related to the immune system, was performed in order to investigate how the ESP and proteins differentially expressed by eggs at different stages of development are potentially able to interact with and stimulate the host immune system (Table 2). The analysis showed that all the samples contain proteins able to stimulate the host immune system, although it is evident that ESP is capable of doing it *via* many different ways. ESP is likely to play a central role in driving the immune response towards a Th2 pattern, since it is predicted to stimulate the production of several interleukins, including IL1, IL3, IL5, IL4 and IL13.

**Table 2:** Immune system pathways predicted by Reactome 3.5 analysis to be stimulated by proteins present in ESP, immature and mature eggs

Type of response	ESP	Immature eggs	Mature eggs
Cytokine signalling in immune response	Interferon signalling: Antiviral mechanism	–	–
	Signalling by Interleukins: IL1, IL3, IL5, GM-CFS, IL4, IL13	–	–
	TNF alpha non-canonical NF-Kappaβ pathway	–	–
Innate immune system	–	Toll-like receptor cascade: Trafficking and processing of endosomal TLR	–
	Neutrophil degranulation	Neutrophil degranulation	Neutrophil degranulation
	Fc gamma receptor (FCGR) dependent phagocytosis	–	–
	Fc epsilon receptor (FCERI) signalling; Toll-like receptor cascade	–	–
	C-type lectin receptors (CLR’s): CLEC7A (Dectin-1)	–	–
	Nucleotide-Binding domain, leucine rich repeat containing receptor (NLR) signalling pathways	–	–
	DDX58/IFIHI mediated induction of IFN alpha/beta pathways	–	–
	Cytosolic sensors of pathogens associated DNA	–	–
Adaptive immune system	MHC class II antigen presentation	MHC class II antigen presentation	MHC class II antigen presentation
	RAP-1 signalling	RAP-1 signalling	–
	MHC class I mediated antigen processing and presentation	–	–
	Signalling by the B cell receptor	–	–
	TRL signalling	–	–

*Note*: Analysis performed using the software Reactome 3.5

## Discussion

Proteomic analysis of a whole eggs extract used eggs purified from mice livers at six weeks post-infection and, therefore, consisted of eggs at different stages of development. It was important to include eggs ranging in development from freshly laid, early zygotic stages, through to fully mature eggs, since this set of data was used as a reference library for subsequent differential analysis of immature and mature eggs. A total of 862 proteins was identified from this hepatic eggs proteome, a composition consistent with other reports of the *S. mansoni* and *S. japonicum* egg proteomes (Additional file 4: Table S1) [23–25].

Although excretory-secretory extracts from various life-cycle stages of *S. mansoni* and *S. japonicum* have been published [8, 25, 26, 37–39], to our knowledge, the work described here is the first to investigate and identify proteins in the ESP of *S. japonicum* eggs. In general, the set of 461 proteins we found in *S. japonicum* ESP is comparable to those components previously characterized in ES products from different developmental stages of *S. japonicum* [39, 40]. Likewise, Liu et al. [23], used high-throughput proteomics to characterize the protein profiles of different developmental stages of *S. japonicum* and identified 1441 egg-associated proteins, with 473 of these being exclusively present in eggs when compared with other life-cycle stages. Although Liu et al. [23] verified an overlapping of proteins across the life-cycle, their analysis showed that certain proteins are stage-enriched or probably expressed in response to environmental stimuli.

When we refined the ESP data through comparison with proteins present in a total egg extract, the complexity of *S. japonicum* ESP, consisting of 95 proteins, still differed considerably from the published data obtained for *S. mansoni* ESP. For example, Cass et al. [26] identified 188 proteins using shotgun proteomic analysis. By contrast, Ashton and colleagues [8] and Mathieson & Wilson [25], allied 1- and 2-DE gels with mass spectrometry, and identified very few proteins in *S. mansoni* ESP, notably Omega-1 and IPSE/alpha-1 proteins, and a few proteases. In another study comparing soluble proteins across four life-cycle stages of *S. mansoni*, including eggs, only 32 distinct proteins were identified [37].

It is important to explain such discrepancies in the number of reported proteins. Mathieson & Wilson [25] attributed the paucity of proteins in ESP products they obtained to the rigorous but gentle processing schedule they used to prepare their egg samples for incubation, notably through the use of saline solutions and trypsin to digest liver samples for egg extraction. The authors further argued that the high egg hatching rates they obtained in *S. mansoni* egg samples after incubation and collection of ESP was evidence of a superior processing schedule that did not kill or rupture eggs. In our study of *S. japonicum* eggs, we also performed hatching assays after incubations to obtain ESP and found high hatching rates similar to those of Mathieson & Wilson [25] (Additional file 3: Figure S3). We therefore consider that the variation of the MS sensitivity, the use of different databases, and the application of different methods to prepare and analyze the samples, e.g. straight shotgun MS analysis [26] or MS analysis of 2-DE gel spots [25, 37], could be reasonable explanations for the differences observed between the studies. We argue that even the most carefully undertaken protocol cannot guarantee that a small number of eggs will not hatch, resulting in contamination, albeit minimal, with contents from inside the eggs. It is also possible that a small number of eggs will hatch or rupture within a host.

As expected, most of the proteins identified in *S. japonicum* ESP were also detected in the proteome of the whole hepatic egg extracts (Additional file 4: Table S1). The fact that 95 proteins were identified exclusively in the ESP does not mean that they are absent in the whole egg, but indicates that these proteins are primarily secreted and therefore transitory in the eggs. Consequently, their presence in a very low concentration in eggs could prevent the identification by the methods used. Proteins such as actin, HSPs, enolase, Glyceraldehyde-3-phosphate dehydrogenase, thioredoxin, proteasome subunits enolase, GLPR1-like protein 1 and cathepsins were among the proteins found in both the whole egg and ESP proteomes. Several proteins identified in the extracts of *S. japonicum* eggs were previously shown to be present in *Schistosoma* spp. eggs, e.g. thioredoxin, tetraspanin, cathepsins, serpins, Sjp40 and SM22.6 [23, 26], although the main antigenic proteins found in *S. mansoni* egg-derived extracts, namely Omega-1, IPSE/ alpha-1 and Kappa-5, were not found in any of the *S. japonicum* extracts. Additionally, various motor proteins (tubulin, actin and myosin), chaperones, structural and binding proteins, as well as proteins involved in metabolism and in signal transduction were identified. Remarkably, most of the proteins present in ESP were associated with extracellular vesicles in our analysis (Fig. 1) which, allied to the prior evidence of cytoskeleton proteins in ES products, suggest that these proteins possibly follow unconventional routes of excretion in parasites [26, 39, 41].

Egg-related proteins were previously shown to have central roles in polarizing the host immune responses to prevent adaptive responses during schistosomiasis. ESPs, such as Sjp40 and tetraspanin, modulate dendritic cells (DC) through specific interaction with Toll-Like receptor (TLR), stimulating cytokines such as IL-4 and IL-10, thereby driving the immune response towards a Th2 pattern that favors parasite survival [12, 42–44]. Reinforcing these data, our pathway analysis demonstrated the ability of *S. japonicum* ESP to stimulate the human immune system through several ways, including elicit secretion of interleukins related to the Th2 response (Table 2).

Of note, *S. japonicum* ESP has been predicted to interact with C-type lectin receptors (CLR’s) in a specific way involving Dectin-1. Although this mechanism is still poorly understood, it was recently demonstrated that *S. mansoni* SEA can induce a Th2 response through specific interactions involving Dectin 1/2 [45]. Notably, Kaiser and colleagues also showed that SEA depleted of Omega-1 glycoprotein was able to trigger a Th2 response [45]. By implication, this observation suggests that *S. japonicum* ESP could stimulate the immune system in a similar Omega-1-independent way.

Gene ontology analyses revealed that *S. japonicum* ESP proteins are mainly associated with neutrophil degranulation (Fig. 1). It is well established that neutrophils play a central role during granuloma formation in response to the presence of schistosome eggs. Indeed, the relative abundance of neutrophils in granulomas is variable in infections with different schistosome species. For example, with schistosomiasis japonica neutrophils accumulate around the eggs eight days after egg deposition starts and the granulomas formed contain higher number of neutrophils than those observed in *S. mansoni* infections [46–48]. Since IPSE/alpha-1 glycoproteins can inhibit the infiltration of neutrophils, the absence of such a protein in *S. japonicum* eggs might explain the elevated number of neutrophils in granulomas around these eggs [6, 49].

Recently Zheng et al. [50] reported that during *S. japonicum* infection, Vγ2 T cells can recruit neutrophils and aggravate liver fibrosis by secreting IL-17A, thereby causing the eggs to be trapped. In addition, it has been shown that neutrophils associated with *S. japonicum* hepatic granuloma, but not *S. mansoni*, release neutrophil extracellular traps (NETs) [6, 51], strongly suggesting that there are major differences in ESP constituency between the two species causing hepato-intestinal schistosomiasis and that further studies may lead to a better understanding of the differential pathogenesis of the two infections.

Major immunomodulatory ESP components of *S. mansoni* eggs are Omega-1 (Ribonuclease-RNase T2) and ISPE [11, 12]. Orthologues of Omega-1 protein have been identified in the genome of *S. japonicum* [52], but it does not appear to be part of the ESP of its eggs. While we did not identify either protein for *S. japonicum* ESP, we did find a Ribonuclease T2 (C1LS35) in both *S. japonicum* eggs and ESP. After BLAST analysis against protein sequences from trematode parasites, we determined that C1LS35 has 32% (score 166, E-value 1.2E-14) identity with the putative hepatotoxic ribonuclease Omega-1 protein from *S. mansoni* (UniProt G4V5C6).

Of interest, recently Ke et al. [4] identified a *S. japonicum* RNase T2 family member, CP1412 protein, as a component of ESP of eggs. Alignment and BLAST analysis have shown that C1LS35 and CP1412 are the same protein, since they have 99% identity. Additional characterization of the CP1412 revealed that, as observed for Omega-1, this Ribonuclease T2 also stimulates polarization of the host Th2 immune response. Further information on the importance of this molecule in the processes of infection and pathogenesis in schistosomiasis japonica might be demonstrated using RNA interference methods.

ESP of schistosome eggs provokes an immune attack to drive the eggs from tissues into the intestinal lumen for excretion from the host. Proteins such as serine, cysteine, aspartyl and metallo-proteases that we identified in *S. japonicum* ESP participate in these fundamental processes, including invasion, escape, and modulation of the host immune system [53, 54], making them suitable targets for the development of vaccines and new treatments [55]. Although most of the proteases identified here remain uncharacterized in terms of egg function, it was previously demonstrated that serine proteases can interfere with host coagulation and the immune response. A 27 kDa serine protease with fibrinolytic activity was characterized from *S. mansoni* eggs and hypothesized to block intravascular fibrin deposition by platelets activated by the eggs [56].

Our analyses demonstrated that protease inhibitors, including serpin, cystatin, serine and cysteine proteases inhibitors respectively, are differentially expressed in mature and immature eggs (Fig. 5). These molecules have central roles in host-parasite interactions [57, 58] and could also represent important targets for future development of drugs and vaccines. Serpins can activate aggregation of platelets and inhibition of fibrin deposition in the vascular endothelium [56], both fundamental processes to guarantee successful infection. The Serpin B6 here demonstrated upregulated in mSj eggs has predicted anti-thrombin activity that might help in parasite propagation, since inhibition of thrombin would culminate in less fibrin available to for clot formation, ultimately helping eggs escape through the intestinal wall. In addition, cystatins contribute to immune evasion and modulation of the Th2 response by activating macrophages and inducing IL-10 production [58–60], and also suppressing exogenous-antigen presentation by DCs, affirming their immunosuppressive role [61].

The comparison of the levels of expression of proteins between immature and mature *S. japonicum* eggs demonstrated important differences in terms of metabolic processes occurring in eggs at different stages of development. Immature eggs primarily produce proteins related to structure and cell organization (e.g. tubulin beta chain, putative dynein and actin depolymerizing factor), mature eggs mostly express proteins involved with energy metabolism and homeostasis regulation. While both immature and mature *S. japonicum* eggs expressed proteins highly involved in neutrophil degranulation process, mSj eggs expressed proteins associated with movement and proteolytic processes, which is likely linked to the presence of an active miracidium and the need for eggs to escape the host and ultimately to hatch.

Mathieson & Wilson [25] compared *S. mansoni* eggs at two different stages of development and showed some 80% similarity in expressed proteins expressed in the two stages. These authors demonstrated that Sm_p40 was an abundant protein in mature S. *mansoni* eggs, while the heat-shock protein-70 (HSP70) was the most abundant protein identified in immature eggs. We also found that proteins related to response to stress were enriched in mSj eggs (e.g. cell wall integrity and stress response component 1) compared with immature, which probably has to do with changes in the environment that mature schistosome eggs have to confront.

Another remarkable difference that we found between immature and mature eggs reflects their predicted ability to stimulate the host immune system (Table 2). Previously, Ashton et al. [8] highlighted that immature *S. mansoni* eggs induce a weaker immune response than the mature forms. By *in vitro* analyzing those proteins more highly expressed by iSj or mSj eggs in terms of their ability to interact and trigger the immune response we found that mature eggs were more likely to activate an innate response by activating T cells (Table 2). The mSj eggs also may elicit adaptive responses through at least two different pathways, including that involving Rap-1 signaling. Rap-1 is a member of the mitogen-activated protein kinase (MAPK) signaling pathways that are pivotal transmitters of extracellular signals, including cytokines. Therefore, once activated, MAPKs can regulate key cellular processes including T cell responses [62, 63]. Taking together the strong potential of ESP for stimulating host immune responses, our data highlight the complexity and significance of immunomodulatory events that *S. japonicum* eggs trigger in order to maintain the life-cycle of the parasite.

## Conclusions

In the present study, we present a list of 957 egg and egg-derived excretory-secretory proteins from *S. japonicum* of which 95 were exclusively found in *S. japonicum* ESP; such proteins are completely accessible to the host immune system effector elements, and therefore in general represent interesting targets for diagnosis and as vaccines. Often, proteins from ESP are involved in key functions that permit schistosome eggs withstand stressful environmental features, including host immune responses that lead to the granulomatous inflammatory reaction observed around the eggs trapped in tissues, but also assist their passage across the gut wall. Further investigation of some of the key proteins differentially expressed in *S. japonicum* eggs and ESP, and even in immature and mature eggs, can lead to better strategies to avoid severe morbidity during schistosomiasis japonica, as well as improving diagnosis, treatment and control of this persistent parasitic infection.

## Additional files

**Additional file 1: Figure S1.** *S. japonicum* mature eggs isolation. Eggs isolated from mice feces after six weeks of infection.

**Additional file 2: Figure S2.** *S. japonicum* immature eggs isolation. **a** *S. japonicum* female worm in the media laying eggs. **b** *S. japonicum* immature eggs isolated after 24 h incubation.

**Additional file 3: Figure S3.** *S. japonicum* egg secretory proteins production. **a** *S. japonicum* liver eggs incubated in a well with RPMI media (time 0 h). White arrow indicates immature eggs, black arrow indicates mature eggs. **b** *S. japonicum* liver eggs incubated in a well with RPMI media, at 37 °C, 5% CO_2_ (time 3 h). **c** *S. japonicum* miracidia. After incubation in RPMI media the eggs were induced to hatch by being placed in water and exposed to direct light for 1 h.

**Additional file 4: Table S1.** List of proteins identified in the whole eggs and those shared with ESP from *S. japonicum* eggs.

**Additional file 5: Table S2.** Proteins identified with significant variation in *S. japonicum* immature eggs compared with mature eggs, listed in descending order of variation.

## Abbreviations

CLR’s: C-type lectin receptors
DC: Dendritic cells
ESP: Egg secretory proteins
FASP: Filter Aided Sample Preparation Method
ICAM-1: Intercellular Adhesion Molecule 1
IDA: Information Dependent Acquisition
IL: Interleukins
IPSE/ alpha-1: IL-4-inducing principle
iSj: Immature *S. japonicum* eggs
LC-MS/MS: Liquid chromatography with mass spectrometry
mSj: Mature *S. japonicum* eggs
NETs: Neutrophil extracellular traps
SEA: Soluble egg antigens
SecP: SecretomeP analysis
SWATH: Sequential window acquisition of all theoretical fragment ion spectra
Th2: T helper 2
TLR: Toll-Like receptor
VCAM-1: Vascular cell adhesion molecule 1
