# Supplementary figures and images for: Qualitative and quantitative proteomic analyses of Schistosoma japonicum eggs and egg-derived secretory-excretory proteins
Source: Parasit Vectors. 2019 Apr 16;12:173. doi: 10.1186/s13071-019-3403-1 (PMC6469072; doi:10.1186/s13071-019-3403-1)

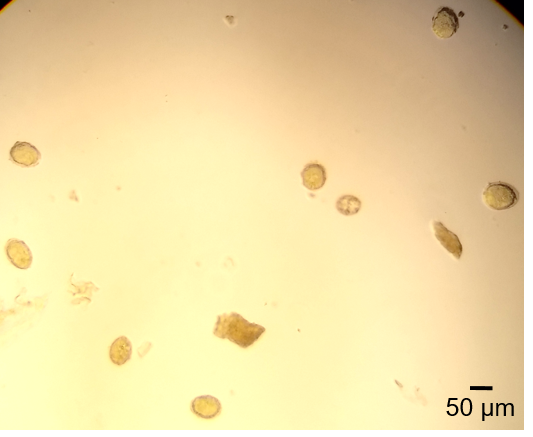

Supplement: Supplementary file 1 — Additional file 1: Figure S1. S. japonicum mature eggs isolation. Eggs isolated from mice feces after six weeks of infection. [file 13071_2019_3403_MOESM1_ESM.png]

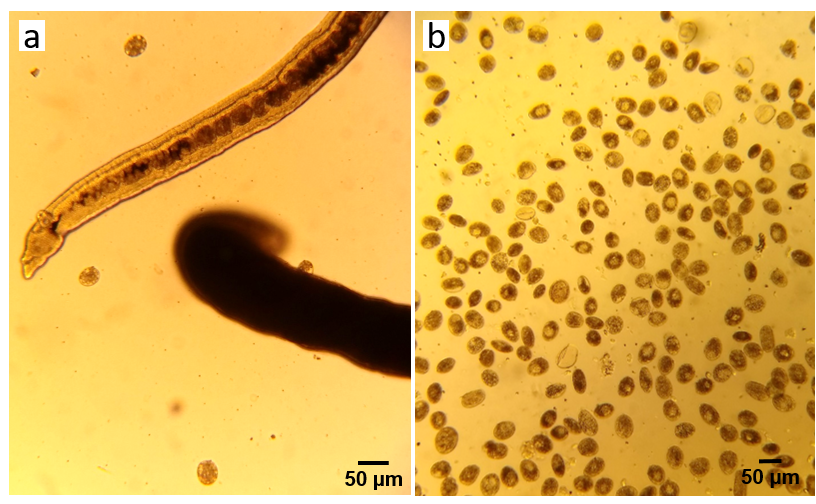

Supplement: Supplementary file 2 — Additional file 2: Figure S2. S. japonicum immature eggs isolation. a S. japonicum female worm in the media laying eggs. b S. japonicum immature eggs isolated after 24 h incubation. [file 13071_2019_3403_MOESM2_ESM.png]

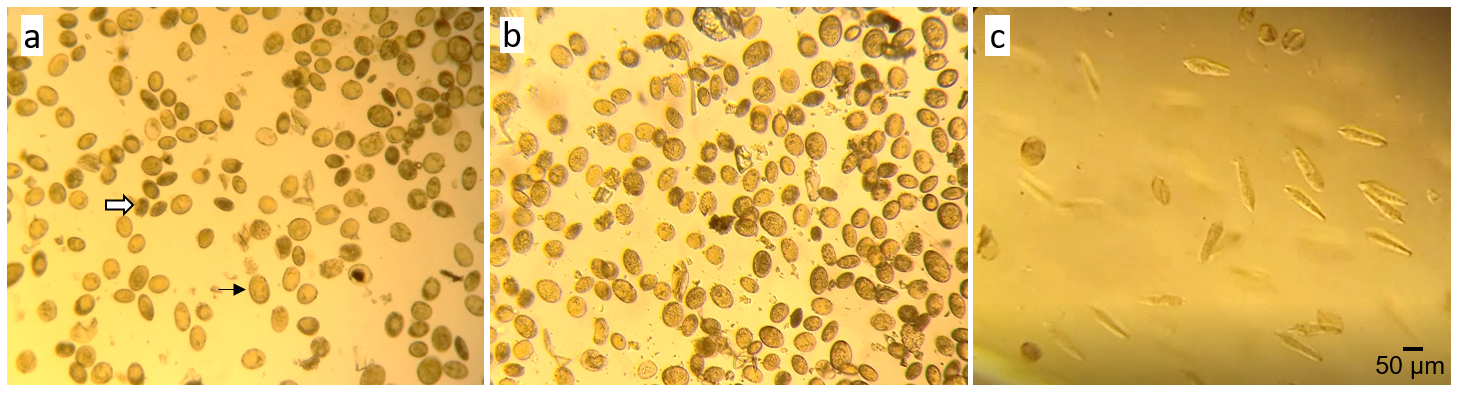

Supplement: Supplementary file 3 — Additional file 3: Figure S3. S. japonicum egg secretory proteins production. a S. japonicum liver eggs incubated in a well with RPMI media (time 0 h). White arrow indicates immature eggs, black arrow indicates mature eggs. b S. japonicum liver eggs incubated in a well with RPMI media, at 37 °C, 5% CO2 (time 3 h). c S. japonicum miracidia. After incubation in RPMI media the eggs were induced to hatch by being placed in water and exposed to direct light for 1 h. [file 13071_2019_3403_MOESM3_ESM.png]
