# Supplementary material for: Qualitative and quantitative proteomic analyses of Schistosoma japonicum eggs and egg-derived secretory-excretory proteins
Source: Parasit Vectors. 2019 Apr 16;12:173. doi: 10.1186/s13071-019-3403-1 (PMC6469072; doi:10.1186/s13071-019-3403-1)
Supplement: Supplementary file 4 — Additional file 4: Table S1. List of proteins identified in the whole eggs and those shared with ESP from S. japonicum eggs. [file 13071_2019_3403_MOESM4_ESM.docx]

| **Supplementary table 1.** List of proteins identified in the whole eggs and those shared with ESP from *Schistosoma* *japonicum* eggs. | | | | | | | | |
| --- | --- | --- | --- | --- | --- | --- | --- | --- |
| **Accession** | **Protein Name** | **Unused** | **%Cov** | **Peptides (95%)** | **Unused** | **%Cov** | **Peptides (95%)** | **SecP** |
|  |  | **Eggs** | | | **ESP** | | |  |
| tr\|C1L4Z7 | Actin 5C | 105.96 | 84,31 | 254 | 8.71 | 77,38 | 42 | - |
| tr\|Q5DDV5 | Enolase | 119.02 | 98,15 | 227 | 67.96 | 93,08 | 117 | NC |
| tr\|A0A183KH93 | Uncharacterized protein | 4.01 | 92,01 | 211 | - | - | - | - |
| tr\|Q71TT8 | Actin | 26 | 84,31 | 198 | 41.09 | 76,33 | 44 | NC |
| tr\|Q5DH23 | SJCHGC09129 protein | 22 | 80,83 | 196 | - | - | - | - |
| tr\|A0A183JXY1 | Uncharacterized protein | 2 | 92,01 | 192 | - | - | - | - |
| tr\|C7TZI6 | Heat shock protein 60 (Fragment) | 138.17 | 92,48 | 182 | 16.41 | 37,77 | 9 | - |
| tr\|A0A0N7I6P5 | Heat shock protein 70 | 161.72 | 92,50 | 157 | 61.55 | 72,65 | 48 | - |
| tr\|Q5DFZ8 | Fructose-bisphosphate aldolase | 91.95 | 96,42 | 136 | 0 | 88,98 | 115 | - |
| tr\|Q5DF69 | Actin 5C | 20.54 | 85,11 | 131 | 0 | 58,78 | 17 | - |
| tr\|Q5D947 | SJCHGC00820 protein | 143.87 | 89,14 | 124 | 62.01 | 65,50 | 46 | - |
| tr\|Q5DGF3 | SJCHGC06900 protein | 93.2 | 91,21 | 114 | 65.67 | 84,82 | 60 | - |
| tr\|C4QIC0 | Tubulin beta chain | 69.28 | 86,48 | 107 | 2 | 63,05 | 33 | NC |
| tr\|C1LVE4 | Tubulin beta chain | 8 | 91,80 | 99 | - | - | - | NC |
| tr\|C1LK99 | Purine nucleoside phosphorylase | 55.04 | 96,85 | 96 | 40.17 | 81,52 | 60 | NC |
| tr\|B3GUU7 | Putative uncharacterized protein | 113.8 | 81,01 | 92 | 40.55 | 53,70 | 31 | SP |
| tr\|A0A183LF13 | Fructose-bisphosphate aldolase | 2.01 | 97,25 | 92 | - | - | - | - |
| tr\|C1LJM5 | Malate dehydrogenase | 62.31 | 90,03 | 86 | 42.94 | 72,14 | 56 | - |
| tr\|Q5D8S4 | Citrate synthase | 79.09 | 82,98 | 83 | 19.23 | 47,65 | 12 | NC |
| tr\|Q27776 | Glyceraldehyde-3-phosphate dehydrogenase | 70.79 | 94,66 | 83 | 34.19 | 69,52 | 33 | - |
| tr\|Q5D9T6 | S-methyl-5'-thioadenosine phosphorylase | 61.18 | 94,65 | 79 | 30.18 | 70,23 | 23 | - |
| tr\|C1LFC4 | Glutamyl aminopeptidase | 70.51 | 49,79 | 76 | 63.69 | 48,78 | 73 | NC |
| tr\|G4V865 | Tubulin alpha chain | 60.35 | 76,77 | 74 | 38.35 | 60,39 | 35 | - |
| tr\|C1LD27 | Glyceraldehyde-3-phosphate dehydrogenase | 2 | 94,66 | 73 | 2 | 69,52 | 27 | - |
| tr\|G4XPB3 | Thioredoxin glutathione reductase | 81.3 | 89,42 | 70 | 2 | 72,14 | 32 | NC |
| tr\|C1LB64 | Tryparedoxin peroxidase | 9.56 | 79,35 | 68 | - | - | - | NC |
| tr\|C1LV50 | Tryparedoxin peroxidase | 6 | 75,54 | 68 | - | - | - | NC |
| tr\|B3GUU0 | Tyrosine 3-monooxygenase/tryptophan 5-monooxygenase activation protein, beta polypeptide | 70.42 | 93,30 | 67 | 14.11 | 8.345 | 36 | - |
| tr\|C1LV44 | Tryparedoxin peroxidase | 48.67 | 75,54 | 66 | 20.03 | 66,29 | 27 | NC |
| tr\|G4XPB5 | Thioredoxin glutathione reductase | 2 | 88,01 | 65 | 48.83 | 73,62 | 30 | - |
| tr\|C1LIA5 | Dihydrolipoyl dehydrogenase | 62.54 | 71,82 | 64 | - | - | - | NC |
| tr\|A0A183LCR5 | Uncharacterized protein | 2.02 | 75,92 | 64 | - | - | - | NC |
| tr\|Q5DEY1 | SJCHGC00794 protein | 44.91 | 74,33 | 63 | 17.6 | 69,91 | 24 | SP |
| tr\|Q5DF59 | Malate dehydrogenase | 84.9 | 91,51 | 62 | 28.65 | 70,30 | 19 | - |
| tr\|Q5DAM7 | Pyruvate kinase | 66.3 | 83,42 | 59 | 24.93 | 58,10 | 14 | NC |
| tr\|Q5D938 | Putative uncharacterized | 40.79 | 73,00 | 59 | 31.87 | 7.178 | 44 | SP |
| tr\|C1LN60 | Uncharacterized protein | 2 | 63,77 | 59 | 2 | 62,56 | 44 | SP |
| tr\|C1LGS7 | Transketolase | 86.16 | 94,23 | 58 | 43.39 | 70,19 | 27 | NC |
| tr\|Q5DDC4 | SJCHGC01945 protein | 65.34 | 84,02 | 58 | 30.79 | 55,94 | 19 | SP |
| tr\|Q5DA69 | SJCHGC01759 protein | 30.79 | 94,09 | 57 | 33.91 | 87,40 | 40 | - |
| tr\|C1LN62 | Uncharacterized protein | 11.3 | 68,69 | 57 | 8.64 | 65,64 | 39 | SP |
| tr\|C1LGS6 | Transketolase | 2 | 91,50 | 57 | - | - | - | NC |
| tr\|Q5D9C5 | SJCHGC09453 protein | 85.41 | 78,43 | 56 | 4.44 | 19,58 | 2 | - |
| tr\|C1L502 | Protein disulfide-isomerase | 83.38 | 92,97 | 55 | 41.84 | 80,79 | 25 | SP |
| tr\|C1LL13 | ATP synthase subunit beta | 62.56 | 84,53 | 54 | 23.27 | 59,38 | 23 | NC |
| tr\|C1LK30 | Glucose-6-phosphate isomerase | 55.81 | 80,44 | 54 | 41.13 | 71,85 | 38 | - |
| tr\|C1LHH0 | Cell division cycle and apoptosis regulator protein 1 | 80.43 | 72,71 | 53 | 3.92 | 25,02 | 2 | - |
| tr\|C1LIN3 | Chaperonin containing TCP1, subunit 7 | 66.27 | 91,87 | 53 | 6.61 | 3.395 | 3 | - |
| tr\|C1LQJ2 | Uncharacterized protein | 30.97 | 60,39 | 53 | 23.98 | 52,67 | 34 | SP |
| tr\|Q26513 | Glutathione S-Transferase | 37.26 | 93,36 | 51 | 17.93 | 72,99 | 28 | - |
| tr\|Q5DC05 | SJCHGC05973 protein | 32.98 | 92,39 | 51 | 16.18 | 85,32 | 24 | - |
| tr\|Q5DCT4 | Adenosylhomocysteinase | 48.11 | 54,40 | 50 | 19.28 | 46,05 | 13 | NC |
| tr\|C1LCI7 | Glutathione S-Transferase | 0.16 | 93,36 | 50 | 2 | 72,99 | 30 | - |
| tr\|A0A0P0EPT8 | Heat shock protein 110 | 55.81 | 64,52 | 49 | 29.45 | 33,64 | 17 | - |
| tr\|Q5C296 | SJCHGC01885 protein (Fragment) | 93.41 | 81,44 | 48 | - | - | - | - |
| tr\|C1LFZ8 | Arginine kinase | 78.85 | 76,39 | 47 | 71.61 | 76,26 | 39 | - |
| tr\|Q5DF55 | SJCHGC06338 protein | 58.36 | 84,03 | 46 | 8.09 | 4.065 | 6 | NC |
| tr\|Q5DEG0 | Transaldolase | 44.23 | 79,50 | 46 | 33.46 | 69,41 | 31 | NC |
| tr\|C1L5C5 | Putative aminopeptidase | 62.75 | 85,40 | 45 | 32.7 | 64,49 | 19 | NC |
| tr\|A0A183QVR5 | Histone H4 | 38.5 | 89,13 | 45 | 16.65 | 63,77 | 31 | - |
| tr\|Q5DD64 | Aldo-keto reductase family 1, member B4 (Aldose reductase) | 17.69 | 91,28 | 45 | 6.01 | 63,55 | 20 | - |
| tr\|C1L542 | Chaperonin containing TCP1, subunit 2 (Beta) | 61.05 | 86,22 | 44 | - | - | - | - |
| tr\|C1L524 | Dihydropyrimidinase-like 2 | 52.78 | 79,18 | 44 | 10.46 | 33,45 | 7 | - |
| tr\|C1LRD1 | Carbonyl reductase 1 | 44.54 | 98,18 | 44 | 4 | 81,95 | 45 | - |
| tr\|C1LRQ1 | Ferritin | 35.57 | 83,24 | 44 | 2 | 67,04 | 22 | NC |
| tr\|C1LH70 | Dipeptidylpeptidase 3 | 71.57 | 74,62 | 43 | 33.74 | 43,30 | 18 | NC |
| tr\|O45034 | Calreticulin | 60.54 | 82,58 | 43 | - | - | - | SP |
| tr\|C1LRF1 | Aldo-keto reductase family 1, member B4 (Aldose reductase) | 51.02 | 78,71 | 43 | 28.08 | 61,61 | 21 | - |
| tr\|Q5C1P9 | SJCHGC07313 protein (Fragment) | 46.58 | 75,23 | 43 | 16.34 | 57,20 | 12 | SP |
| tr\|Q7Z1I3 | L-lactate dehydrogenase | 8.78 | 89,73 | 43 | 4.04 | 53,46 | 24 | NC |
| tr\|C1LQ45 | Calreticulin | 3.78 | 85,10 | 43 | 28.52 | 7.904 | 22 | SP |
| tr\|O96409 | Ferritin | 2.01 | 83,24 | 43 | 18.67 | 67,04 | 22 | NC |
| tr\|C1L5N9 | T-complex protein 1 subunit delta | 52.28 | 84,75 | 42 | - | - | - | NC |
| tr\|O96460 | Protein disulfide-isomerase (Fragment) | 43.99 | 78,75 | 42 | - | - | - | NC |
| tr\|G4V910 | Putative heat shock protein 70 (Hsp70) | 2.02 | 54,47 | 42 | - | - | - | SP |
| tr\|C1LJA9 | Glutamate dehydrogenase | 63.34 | 81,01 | 41 | 25.37 | 44,74 | 13 | - |
| tr\|C1L860 | L-lactate dehydrogenase | 47.65 | 89,73 | 41 | 23.19 | 59,52 | 23 | NC |
| tr\|C1LRC9 | Carbonyl reductase 1 | 2.61 | 98,18 | 41 | - | - | - | - |
| tr\|C1LI13 | T-complex protein 1 subunit delta | 0.21 | 84,00 | 41 | 11.44 | 36,80 | 5 | NC |
| tr\|C1LGT2 | Glucan (1,4-alpha-), branching enzyme 1 | 65.49 | 67,25 | 40 | 37.94 | 58,77 | 21 | NC |
| tr\|C1L457 | Leukotriene A4 hydrolase | 60.09 | 60,10 | 40 | 33.29 | 5.224 | 19 | NC |
| tr\|Q5DE66 | Major egg antigen (P40) | 45.26 | 80,51 | 40 | - | - | - | - |
| tr\|G4V5L4 | Putative chaperonin containing t-complex protein 1, zeta subunit, tcpz | 35.73 | 61,97 | 40 | - | - | - | NC |
| tr\|Q86DW6 | Hydroxyacylglutathione hydrolase | 34.68 | 82,38 | 40 | 18.87 | 67,43 | 15 | - |
| tr\|C4QHD7 | Histone H3 | 29.97 | 92,65 | 40 | - | - | - | NC |
| tr\|C1LGW8 | Chaperonin containing TCP1, subunit 2 (Beta) | 4 | 88,87 | 40 | - | - | - | - |
| tr\|C1LRE9 | Aldo-keto reductase family 1, member B4 (Aldose reductase) | 0.28 | 73,22 | 40 | - | - | - | - |
| tr\|A0A183MUN0 | Uncharacterized protein | 64.96 | 47,13 | 39 | 3.85 | 29,85 | 12 | - |
| tr\|Q5D8L5 | SJCHGC06436 protein | 34.36 | 85,47 | 39 | 21.13 | 63,37 | 20 | NC |
| tr\|C1L566 | Alpha-1,4 glucan phosphorylase | 59.63 | 65,06 | 38 | 30.95 | 46,61 | 16 | NC |
| tr\|C1LMS0 | Chaperonin containing TCP1, subunit 5 (Epsilon) | 47.86 | 86,23 | 38 | - | - | - | - |
| tr\|Q5DDU9 | Histone H2B | 24.98 | 84,42 | 38 | 9.33 | 62,30 | 20 | - |
| tr\|C1L537 | Glucan (1,4-alpha-), branching enzyme 1 | 2.04 | 68,26 | 38 | 2 | 59,64 | 21 | NC |
| tr\|C1LGD4 | Ubiquinol-cytochrome-c reductase complex core protein 2, mitochondrial | 54.09 | 83,00 | 37 | - | - | - | NC |
| tr\|Q5DB51 | 2-deoxy-D-gluconate 3-dehydrogenase | 32.53 | 76,6 | 37 | 20.03 | 46,79 | 18 | - |
| tr\|O96398 | Myosin (Fragment) | 0.7 | 76,42 | 37 | - | - | - | - |
| tr\|Q5DGD4 | SJCHGC06677 protein | 59.3 | 65,49 | 36 | - | - | - | - |
| tr\|C1LEZ9 | Putative TyrA protein | 58 | 85,21 | 36 | - | - | - | NC |
| tr\|Q5DFY5 | SJCHGC09310 protein | 54.82 | 76,28 | 36 | - | - | - | - |
| tr\|Q86DV3 | Annexin | 54.22 | 81,20 | 36 | - | - | - | - |
| tr\|Q5DFR7 | SJCHGC06291 protein | 32.7 | 82,40 | 36 | 11.96 | 58,79 | 14 | NC |
| tr\|A0A183MVA0 | Uncharacterized protein | 0.06 | 37,70 | 36 | 0.07 | 31,15 | 17 | - |
| tr\|C1LL84 | Alpha-glucosidase | 64.04 | 64,16 | 35 | 11.29 | 35,83 | 5 | NC |
| tr\|C1LM88 | 20S proteasome subunit alpha 7 | 41.69 | 76,77 | 35 | 22.97 | 55,90 | 17 | - |
| tr\|Q5DDI7 | Gelsolin | 52.09 | 87,26 | 34 | 23.61 | 54,57 | 14 | - |
| tr\|C1LMQ9 | Triosephosphate isomerase | 33.69 | 82,53 | 34 | 17.57 | 62,30 | 19 | NC |
| tr\|Q9U8F3 | Thioredoxin | 19.63 | 83,02 | 34 | 20.15 | 83,02 | 23 | NC |
| tr\|Q5DE07 | Succinate dehydrogenase [ubiquinone] flavoprotein subunit, mitochondrial | 56.04 | 66,72 | 33 | 1.07 | 25,88 | 6 | - |
| tr\|C1L6T6 | Hypotherical protein | 49.67 | 75,92 | 33 | - | - | - | - |
| tr\|Q5BXP5 | SJCHGC05927 protein (Fragment) | 40.1 | 61,48 | 33 | 13.31 | 34,67 | 8 | NC |
| tr\|Q7YW79 | Elongation factor 1-alpha | 39.5 | 83,23 | 33 | - | - | - | - |
| tr\|Q5DCH6 | Superoxide dismutase | 34.9 | 88,78 | 33 | 8.24 | 39,46 | 8 | NC |
| tr\|G4VI41 | Putative chaperonin containing t-complex protein 1, eta subunit, tcph | 2.03 | 65,86 | 33 | - | - | - | - |
| tr\|Q5DGY9 | Heat shock 70kDa protein 9B | 56.49 | 76,10 | 32 | 7.54 | 3.828 | 5 | - |
| tr\|C1LI70 | Putative Mitochondrial processing peptidase beta subunit, mitochondrial | 51.67 | 70,03 | 32 | - | - | - | NC |
| tr\|C1LIX6 | Loss of heterozygosity 11 chromosomal region 2 gene A protein homolog | 43.14 | 50,84 | 32 | - | - | - | NC |
| tr\|C1L674 | Arginase | 50.59 | 83,24 | 31 | - | - | - | - |
| tr\|A0A183R6B1 | ATP synthase subunit alpha | 44.37 | 65,64 | 31 | 24.8 | 41,17 | 16 | - |
| tr\|C1L8P3 | Voltage-dependent anion-selective channel 2 | 43.82 | 86,87 | 31 | - | - | - | - |
| tr\|C7TZ54 | Endothelin converting enzyme 2 | 43.03 | 39,30 | 31 | - | - | - | NC |
| tr\|Q540A3 | Clone ZZD245 mRNA sequence | 39.18 | 77,06 | 31 | 14.98 | 64,67 | 9 | - |
| tr\|Q86E11 | Clone ZZZ345 mRNA sequence | 12.04 | 97,92 | 31 | - | - | - | NC |
| tr\|C1L6P8 | Calnexin | 51.11 | 77,25 | 30 | - | - | - | SP |
| tr\|Q5DE25 | SJCHGC06488 protein | 50.8 | 94,88 | 30 | 8.51 | 43,43 | 4 | NC |
| tr\|Q5C1F3 | SJCHGC04997 protein (Fragment) | 41.04 | 79,36 | 29 | 26.91 | 65,86 | 15 | - |
| tr\|Q5DAN5 | SJCHGC01391 protein | 36.65 | 71,92 | 29 | 9.43 | 4.408 | 5 | - |
| tr\|C1L9K4 | Thioredoxin | 35.17 | 83,02 | 29 | 5.43 | 83,02 | 20 | NC |
| tr\|C1LLI7 | Proteasome subunit alpha type | 33.11 | 90,49 | 29 | 11.39 | 45,24 | 5 | - |
| tr\|Q95VB7 | Albumin | 15 | 53,13 | 29 | 23.78 | 58,21 | 33 | SP |
| tr\|C1L6T8 | Hypotherical protein | 2.02 | 72,86 | 29 | - | - | - | - |
| tr\|A0A183MVD8 | Uncharacterized protein | 42.32 | 60,40 | 28 | 8.02 | 32,78 | 4 | - |
| tr\|Q86DY0 | Putative tyrosine 3-monooxygenase | 36.58 | 75,19 | 28 | 20.29 | 6.463 | 16 | - |
| tr\|A0A183R3C1 | Uncharacterized protein | 3.38 | 41,17 | 28 | - | - | - | - |
| tr\|B1PS35 | Paramyosin | 56.86 | 61,65 | 27 | - | - | - | - |
| tr\|Q5DES2 | Proteasome (Prosome, macropain) subunit, alpha type 6 | 32.2 | 93,93 | 27 | 7.68 | 43,32 | 9 | - |
| tr\|Q5DHB0 | SJCHGC06638 protein | 31.33 | 93,55 | 27 | 6.22 | 41,94 | 4 | - |
| tr\|A0A095AGK7 | Alpha-1,4 glucan phosphorylase | 2.14 | 50,05 | 27 | 0.66 | 4.031 | 11 | - |
| tr\|C1LNX4 | Voltage-dependent anion-selective channel 2 | 0.27 | 86,87 | 27 | 8.14 | 29,08 | 4 | - |
| tr\|G4V9U9 | Putative major vault protein | 37.07 | 54,25 | 26 | - | - | - | - |
| tr\|Q5D8I3 | Deoxyhypusine hydroxylase | 35.02 | 77,31 | 26 | 19.47 | 48,46 | 13 | NC |
| tr\|Q5DE13 | SJCHGC07024 protein | 34.2 | 92,33 | 26 | 21.44 | 6.863 | 17 | - |
| tr\|Q5DBC8 | Aspartate aminotransferase | 26.4 | 65,23 | 26 | 23.52 | 57,30 | 25 | NC |
| tr\|B3W660 | Putative uncharacterized protein | 22.42 | 62,00 | 26 | - | - | - | SP |
| tr\|C7TQR4 | Ribonuclease X25 | 17.95 | 68,27 | 26 | - | - | - | SP |
| tr\|C7TQS0 | Putative Ribonuclease Oy | 5.88 | 70,03 | 26 | - | - | - | SP |
| tr\|A0A183JNP8 | Uncharacterized protein | 2.39 | 45,75 | 26 | - | - | - | - |
| tr\|C1L5V1 | Adenine phosphoribosyltransferase | 2 | 93,55 | 26 | - | - | - | - |
| tr\|C1L6P9 | Calnexin | 2 | 72,85 | 26 | - | - | - | SP |
| tr\|B1PS36 | Paramyosin | 0.09 | 64,20 | 26 | 13.1 | 37,52 | 6 | - |
| tr\|C1LFZ6 | Serine hydroxymethyltransferase | 42.31 | 87,58 | 25 | - | - | - | - |
| tr\|Q5C1E2 | SJCHGC06674 protein (Fragment) | 39.59 | 88,30 | 25 | 12.58 | 72,45 | 6 | NC |
| tr\|A0A183QF49 | Uncharacterized protein | 38.23 | 36,77 | 25 | - | - | - | - |
| tr\|C1LHZ3 | GLIPR1-like protein 1 | 26.89 | 59,02 | 25 | 10.23 | 52,46 | 18 | SP |
| tr\|Q86H19 | Nucleoside diphosphate kinase (Fragment) | 17.55 | 87,26 | 25 | 13.04 | 87,26 | 18 | - |
| tr\|Q86H19 | Nucleoside diphosphate kinase (Fragment) | 17.55 | 87,26 | 25 | 13.04 | 87,26 | 18 | - |
| tr\|Q5C327 | SJCHGC07169 protein (Fragment) | 9.37 | 64,84 | 25 | 6.32 | 67,11 | 23 | - |
| tr\|Q5DEF8 | SJCHGC07058 protein | 7.53 | 88,60 | 25 | - | - | - | - |
| tr\|C1LMQ4 | Uncharacterized protein | 2.19 | 71,68 | 25 | - | - | - | - |
| tr\|Q5DA59 | SJCHGC01783 protein | 39.72 | 75,27 | 24 | 29.58 | 55,72 | 15 | - |
| tr\|Q5DEZ9 | Proteasome subunit beta type | 38.15 | 60,50 | 24 | 10.06 | 28,11 | 6 | - |
| tr\|Q86DX7 | Phosphoglycerate kinase (Fragment) | 35.95 | 88,01 | 24 | - | - | - | - |
| tr\|B3GUV6 | Phosphoglycerate mutase | 31.78 | 91,20 | 24 | 10.31 | 48,39 | 9 | - |
| tr\|Q5MB11 | Hypoxanthine phosphoribosyltransferase 1 | 28.53 | 93,94 | 24 | 5.44 | 27,27 | 4 | - |
| tr\|Q5DBQ9 | SJCHGC01755 protein | 20.18 | 71,71 | 24 | 21.28 | 56,56 | 20 | - |
| tr\|C1LHD3 | T-complex protein 1 subunit gamma | 10.19 | 89,46 | 24 | - | - | - | NC |
| tr\|C1LKB8 | Prohibitin-2 (B-cell receptor-associated protein BAP37) | 36.35 | 84,82 | 23 | - | - | - | NC |
| tr\|Q5DFE9 | Proteasome subunit alpha type | 31.95 | 74,88 | 23 | 11.51 | 60,00 | 8 | - |
| tr\|Q5DVX4 | Peroxiredoxin 5037 | 25.48 | 76,81 | 23 | 7.74 | 40,00 | 9 | NC |
| tr\|C1LCF0 | Peptidyl-prolyl cis-trans isomerase | 22.83 | 88,41 | 23 | - | - | - | - |
| tr\|U3N6C3 | Lethal giant larvae protein | 37.7 | 40,97 | 22 | - | - | - | NC |
| tr\|A0A094ZHZ3 | V-type proton ATPase subunit B | 36.67 | 73,25 | 22 | - | - | - | NC |
| tr\|C1LFP4 | Putative aldehyde dehydrogenase 1B1 | 32.65 | 75,76 | 22 | 21.51 | 46.02 | 16 | NC |
| tr\|Q5DHC0 | Proteasome subunit beta type | 27.21 | 76,88 | 22 | 10.24 | 55,29 | 10 | - |
| tr\|C1LH45 | Fructose-1,6-bisphosphatase | 26.58 | 67,04 | 22 | 18.81 | 42,60 | 13 | NC |
| tr\|C1L4K7 | Putative catechol-O-methyltransferase domain containing 1 | 24.75 | 64,75 | 22 | - | - | - | NC |
| tr\|Q5DA88 | Proteasome subunit beta type | 23.59 | 92,91 | 22 | 10.06 | 45,28 | 5 | - |
| tr\|C1LNY3 | Superoxide dismutase [Cu-Zn] | 21.05 | 92,90 | 22 | 16.75 | 74,55 | 19 | NC |
| tr\|Q5DF77 | SJCHGC01853 protein | 17.63 | 68,84 | 22 | 18.54 | 69,95 | 25 | - |
| tr\|Q5C1V0 | SJCHGC07757 protein (Fragment) | 15.84 | 61,40 | 22 | - | - | - | NC |
| tr\|A0A183QL58 | Transaldolase | 6.5 | 44,24 | 22 | 4 | 37,88 | 16 | NC |
| tr\|C1LLL8 | Proteasome subunit alpha type | 0.06 | 96,36 | 22 | - | - | - | - |
| tr\|Q86F27 | Clone ZZD1165 mRNA sequence | 31.05 | 88,13 | 21 | - | - | - | NC |
| tr\|Q86BY2 | Adenylate kinase | 28.08 | 84,76 | 21 | 7.83 | 60,40 | 9 | NC |
| tr\|A0A183N2T0 | Uncharacterized protein | 26.39 | 63,51 | 21 | - | - | - | - |
| tr\|C7TZP2 | NADH:ubiquinone oxidoreductase complex I intermediate-associated protein 30,domain-containing protein (Fragment) | 20.03 | 47,85 | 21 | 15.57 | 52,99 | 18 | SP |
| tr\|Q5C3V3 | SJCHGC05011 protein (Fragment) | 18.91 | 84,61 | 21 | 5.35 | 42,53 | 3 | - |
| tr\|C1LQK0 | Uncharacterized protein | 7.17 | 45,96 | 21 | 3.06 | 4.194 | 14 | SP |
| tr\|Q5DFW7 | SJCHGC09409 protein | 6.07 | 65,39 | 21 | - | - | - | NC |
| tr\|A0A183RMS5 | Succinate dehydrogenase [ubiquinone] flavoprotein subunit, mitochondrial | 4.08 | 67,83 | 21 | 9.53 | 42,71 | 6 | NC |
| tr\|B3W670 | Heme-binding protein 2 | 2 | 88,13 | 21 | 6.76 | 32,76 | 4 | NC |
| tr\|Q86F62 | Proteasome subunit alpha type | 39.02 | 96,36 | 20 | 12.38 | 44,35 | 6 | - |
| tr\|C1L8V7 | Proliferating cell nuclear antigen | 34.01 | 71,92 | 20 | - | - | - | NC |
| tr\|C1LFX5 | Proteasome subunit alpha type | 30.19 | 84,46 | 20 | 7.75 | 39,01 | 7 | - |
| tr\|C1LFJ1 | Elongation FacTor | 29.33 | 70,53 | 20 | 15.58 | 39,41 | 7 | - |
| tr\|C7TXR1 | Lymphocyte cytosolic protein 1 | 29.27 | 66,86 | 20 | 8.55 | 2.669 | 4 | - |
| tr\|Q86F38 | Clone ZZD114 mRNA sequence | 26.73 | 71,09 | 20 | 9.98 | 36,73 | 8 | NC |
| tr\|Q86F39 | 20S proteasome subunit beta 4 | 24.64 | 72,64 | 20 | 6.44 | 47,26 | 6 | NC |
| tr\|C1LS73 | Putative peptidylglycine alpha-hydroxylating monooxygenase | 23.58 | 50 | 20 | 22.85 | 35,08 | 17 | SP |
| tr\|C1LS13 | Cell wall integrity and stress response component 1 | 19.24 | 56,25 | 20 | 18.4 | 46,48 | 15 | - |
| tr\|G4VFE8 | Histone H2 | 17.28 | 56,00 | 20 | - | - | - | NC |
| tr\|Q5C2Y3 | SJCHGC05326 protein (Fragment) | 4.01 | 62,30 | 20 | 4.02 | 60,44 | 17 | - |
| tr\|A0A183PJU8 | Uncharacterized protein | 1.14 | 44,56 | 20 | - | - | - | - |
| tr\|A0A094ZVZ1 | Glycerol-3-phosphate dehydrogenase | 33.92 | 50,74 | 19 | - | - | - | SP |
| tr\|B5B7Q8 | Fatty acid binding protein 7, brain | 31.94 | 95,45 | 19 | - | - | - | - |
| tr\|A0A183QI45 | Uncharacterized protein | 30.23 | 52,60 | 19 | - | - | - | - |
| tr\|Q5DCA0 | Hypotherical protein | 25.15 | 61,64 | 19 | - | - | - | - |
| tr\|C1L730 | Peptidyl-prolyl cis-trans isomerase | 18.02 | 72,04 | 19 | 14.34 | 52,39 | 13 | SP |
| tr\|Q5D959 | SJCHGC06651 protein | 8.65 | 88,88 | 19 | - | - | - | - |
| tr\|C1LM50 | Proteasome subunit beta type | 2 | 92,91 | 19 | - | - | - | - |
| tr\|Q5C3T7 | SJCHGC06627 protein (Fragment) | 2 | 87,29 | 19 | - | - | - | NC |
| tr\|C1LFU0 | Tubulin alpha chain | 0.37 | 47,26 | 19 | - | - | - | NC |
| tr\|Q95WT4 | Na/K-transporting ATPase subunit alpha | 32.57 | 37,83 | 18 | 2.92 | 15,28 | 2 | NC |
| tr\|Q5DI18 | SJCHGC02266 protein | 31.49 | 70,28 | 18 | 19.31 | 6.047 | 10 | NC |
| tr\|C1LJU5 | Putative actin-interacting protein 1 | 28.67 | 61,12 | 18 | 31.29 | 55,65 | 18 | NC |
| tr\|Q5C3M2 | SJCHGC03231 protein (Fragment) | 20.93 | 60,19 | 18 | 13.19 | 55,09 | 11 | - |
| tr\|C7TYR0 | Proteasome subunit beta type | 19.87 | 71,21 | 18 | 11.16 | 58,53 | 8 | - |
| tr\|Q5DFE8 | SJCHGC05888 protein | 32.86 | 71,39 | 17 | - | - | - | SP |
| tr\|C1LFM1 | Stomatin-like protein 2 | 27.55 | 69,78 | 17 | - | - | - | NC |
| tr\|Q5C1N0 | SJCHGC06754 protein (Fragment) | 25.68 | 59,88 | 17 | 16.07 | 41,62 | 9 | NC |
| tr\|C1LK00 | Proteasome subunit alpha type | 25.58 | 69,23 | 17 | 13.04 | 41,69 | 12 | - |
| tr\|Q5DD07 | SJCHGC06229 protein | 22.35 | 64,85 | 17 | 15.01 | 32,51 | 8 | - |
| tr\|C1LH93 | Ubiquinol-cytochrome c reductase cytochrome c1 | 21.6 | 65,75 | 17 | - | - | - | NC |
| tr\|Q5BXX0 | Nucleotide diphosphatase (Fragment) | 16.71 | 80,90 | 17 | 5.03 | 48,73 | 3 | NC |
| tr\|C1LH44 | Fructose-1,6-bisphosphatase | 2 | 68,41 | 17 | 2 | 40,13 | 10 | NC |
| tr\|C1LS05 | Cell wall integrity and stress response component 1 | 0.9 | 44,92 | 17 | 1.35 | 46,48 | 14 | NC |
| tr\|Q86E33 | Clone ZZZ282 mRNA sequence | 26.31 | 56,38 | 16 | - | - | - | - |
| tr\|Q5C1S0 | SJCHGC02324 protein (Fragment) | 25.32 | 45,44 | 16 | 10.6 | 19,92 | 4 | NC |
| tr\|Q5MB10 | Succinate dehydrogenase [ubiquinone] iron-sulfur subunit, mitochondrial | 24.51 | 75,54 | 16 | - | - | - | NC |
| tr\|C1L5H5 | Proteasome (Prosome, macropain) subunit, beta type, 6 | 22.55 | 65,6 | 16 | - | - | - | - |
| tr\|Q5BYZ9 | SJCHGC06040 protein (Fragment) | 21.36 | 63,91 | 16 | - | - | - | - |
| tr\|Q5DC61 | Nitrilase superfamily protein | 20.83 | 52,41 | 16 | 6.3 | 3.828 | 4 | SP |
| tr\|C1L7Z2 | Enhancer of zeste homolog 1 (ENX-2) | 20.12 | 68,34 | 16 | - | - | - | - |
| tr\|Q5DGI7 | SJCHGC02838 protein | 18.69 | 51,08 | 16 | 9.8 | 23,66 | 6 | SP |
| tr\|C1LI78 | Stringent starvation protein A | 17.38 | 82,16 | 16 | 0.06 | 49,79 | 12 | NC |
| tr\|Q5DBE6 | ADP-ribose pyrophosphatase, mitochondrial | 17.13 | 93,40 | 16 | 10.67 | 56,94 | 13 | NC |
| tr\|C7TZG8 | Myosin heavy chain (Fragment) | 6.51 | 55,59 | 16 | - | - | - | - |
| tr\|A0A183MIN8 | Uncharacterized protein | 6.04 | 36,10 | 16 | - | - | - | NC |
| tr\|C1LI77 | Stringent starvation protein A | 0.58 | 82,99 | 16 | 13.75 | 49,79 | 12 | - |
| tr\|C1LG62 | 1-pyrroline-5-carboxylate dehydrogenase | 25.75 | 60,03 | 15 | 9.02 | 26,33 | 5 | NC |
| tr\|Q5DFC2 | SJCHGC04324 protein | 23.55 | 70,14 | 15 | - | - | - | - |
| tr\|Q5FX73 | Serine/threonine-protein phosphatase | 23.4 | 66,97 | 15 | 11.68 | 34,25 | 6 | NC |
| tr\|C1LL56 | Proteasome subunit beta type | 12.82 | 56,68 | 15 | 4.23 | 26,71 | 7 | NC |
| tr\|C7TY75 | Glycerol-3-phosphate dehydrogenase | 12.07 | 53,61 | 15 | - | - | - | SP |
| tr\|Q5D925 | SJCHGC09142 protein | 10.6 | 76,08 | 15 | 3.56 | 49,27 | 7 | - |
| tr\|C1LS80 | Putative peptidylglycine alpha-hydroxylating monooxygenase | 2.17 | 48,33 | 15 | 4.36 | 34,81 | 13 | SP |
| tr\|O96462 | Ribosomal protein L40 | 2 | 82,81 | 15 | 0.07 | 77,34 | 6 | NC |
| tr\|Q86F28 | BAR,domain-containing protein | 23.63 | 74,90 | 14 | 2.37 | 25,49 | 2 | NC |
| tr\|C1LEW5 | Peptidase M8, leishmanolysin,domain-containing protein | 20.83 | 36,82 | 14 | 13.9 | 31.58 | 7 | - |
| tr\|Q5DB96 | Beta-hexosaminidase | 15.45 | 46,18 | 14 | 2.82 | 14,49 | 3 | SP |
| tr\|Q5BWI4 | SJCHGC04358 protein (Fragment) | 7.45 | 30,86 | 14 | 7.48 | 30,86 | 14 | NC |
| tr\|Q5DD62 | Caspase 3, apoptosis-related cysteine peptidase | 5.96 | 56,25 | 14 | 9.82 | 41,31 | 8 | - |
| tr\|C1LS11 | Cell wall integrity and stress response component 1 | 3.26 | 68,75 | 14 | - | - | - | - |
| tr\|A0A183P9C3 | Uncharacterized protein | 0.22 | 49,75 | 14 | - | - | - | NC |
| tr\|Q5DH83 | Glucose-6-phosphate 1-dehydrogenase | 29.19 | 74,30 | 13 | 20.98 | 5.979 | 8 | - |
| tr\|C1LUI2 | Nascent polypeptide-associated complex subunit alpha | 22.2 | 95,27 | 13 | - | - | - | NC |
| tr\|C1LJL2 | Actin-like protein 3 | 18.79 | 56,29 | 13 | - | - | - | - |
| tr\|C1LD57 | Acetyltransferase component of pyruvate dehydrogenase complex | 18.64 | 57,55 | 13 | - | - | - | - |
| tr\|Q5BZC8 | SJCHGC03398 protein (Fragment) | 18.05 | 63,52 | 13 | 1.07 | 35,19 | 5 | - |
| tr\|C1LPF1 | Caspase 3, apoptosis-related cysteine peptidase | 17.73 | 61,19 | 13 | 2.01 | 35,12 | 5 | - |
| tr\|Q5DI38 | Carboxypeptidase | 16.07 | 38,44 | 13 | 6.99 | 23,71 | 7 | SP |
| tr\|A0A094ZHZ7 | Pyruvate carboxylase | 8.69 | 35,21 | 13 | - | - | - | - |
| tr\|Q5DBX7 | Tubulin alpha chain | 8.13 | 55,80 | 13 | - | - | - | - |
| tr\|C1L7I9 | Glyoxalase I | 0.8 | 68,80 | 13 | - | - | - | NC |
| tr\|C7TXY1 | Putative phosphoglucomutase 2 | 24.75 | 48,17 | 12 | 12.28 | 31,83 | 5 | - |
| tr\|Q86E42 | Annexin | 24.42 | 56,36 | 12 | 18.67 | 4.797 | 9 | NC |
| tr\|C1L425 | Serpin B6 (Placental thrombin inhibitor) | 21.74 | 47,79 | 12 | 10.35 | 26,60 | 5 | - |
| tr\|Q5BYA8 | SJCHGC00475 protein (Fragment) | 20.85 | 72,65 | 12 | 11.89 | 53,24 | 8 | NC |
| tr\|Q86FH9 | Clone ZZZ276 mRNA sequence | 20.07 | 54,29 | 12 | 14.52 | 37,09 | 8 | SP |
| tr\|Q5DF67 | DnaJ homolog, subfamily B, member 4 | 20.05 | 89,24 | 12 | - | - | - | NC |
| tr\|C1LB54 | Eukaryotic translation elongation factor 1 gamma | 19.57 | 47,36 | 12 | 4.29 | 27,27 | 3 | - |
| tr\|Q5C3Z6 | SJCHGC06202 protein (Fragment) | 18.49 | 61,72 | 12 | 4.24 | 35,01 | 2 | NC |
| tr\|Q86ER0 | ATPase, H+ transporting, lysosomal 31kDa, V1 subunit E | 18.43 | 69,33 | 12 | - | - | - | NC |
| tr\|A0A183N888 | Uncharacterized protein | 16.43 | 61,54 | 12 | - | - | - | NC |
| tr\|A0A183K3C2 | Uncharacterized protein | 14.45 | 36,89 | 12 | - | - | - | - |
| tr\|C1LEZ4 | Tegumental protein | 13.99 | 59,21 | 12 | 14.38 | 56,30 | 17 | NC |
| tr\|C1LIB1 | Ribonuclease T2,domain-containing protein | 11.53 | 43,09 | 12 | - | - | - | SP |
| tr\|Q5DBI5 | SJCHGC01741 protein | 8.77 | 70,63 | 12 | 7.39 | 5.687 | 12 | - |
| tr\|Q5DBJ0 | SJCHGC00560 protein | 6.61 | 46,50 | 12 | 0.06 | 24,28 | 4 | - |
| tr\|A0A183MDF7 | Uncharacterized protein | 3.83 | 77,64 | 12 | - | - | - | NC |
| tr\|G4VHU6 | Peroxiredoxin, Prx4 | 1.11 | 40,72 | 12 | - | - | - | NC |
| tr\|G4V9S0 | Adenylate kinase | 0.47 | 81,22 | 12 | 0.77 | 54,82 | 7 | NC |
| tr\|Q5DFD6 | SJCHGC06332 protein | 22.9 | 44,53 | 11 | - | - | - | NC |
| tr\|M4QLF4 | Ago2 | 21.79 | 49,30 | 11 | - | - | - | - |
| tr\|C1LFX7 | Protein kinase | 19.88 | 47,06 | 11 | 13.16 | 36,68 | 6 | - |
| tr\|C1LQM8 | Uncharacterized protein | 19.82 | 64,09 | 11 | 22.31 | 64,09 | 12 | SP |
| tr\|C1LKV4 | Rho GDP-dissociation inhibitor 2 | 18.97 | 81,41 | 11 | - | - | - | - |
| tr\|C1LG70 | Furin-1 | 18.92 | 39,26 | 11 | 12.35 | 37,25 | 6 | SP |
| tr\|Q5DCA9 | SJCHGC06227 protein | 18.87 | 41,76 | 11 | 8.62 | 33,18 | 5 | NC |
| tr\|C1LE16 | Putative TatD DNase domain containing 1 | 18.67 | 60,47 | 11 | 6.11 | 4.476 | 3 | - |
| tr\|C1L5D0 | Phosphotransferase | 18.61 | 53,88 | 11 | - | - | - | - |
| tr\|G4VBW4 | Putative alpha-actinin | 18.51 | 36,09 | 11 | - | - | - | - |
| tr\|Q86F46 | Clone ZZD1120 mRNA sequence | 16.73 | 72,47 | 11 | - | - | - | NC |
| tr\|C1LD67 | Endothelin-converting enzyme 1 | 15.78 | 30,52 | 11 | - | - | - | NC |
| tr\|C1LUR6 | Peptidase inhibitor 16 | 15.02 | 52,38 | 11 | 14.24 | 74,59 | 11 | SP |
| tr\|C1LMW5 | Universal stress protein | 13.6 | 88,12 | 11 | - | - | - | - |
| tr\|C1LQN6 | Uncharacterized protein | 12.7 | 31,87 | 11 | 12.42 | 32,55 | 10 | SP |
| tr\|Q5DFP6 | SJCHGC00967 protein | 11.69 | 55,26 | 11 | 3.79 | 24,01 | 4 | NC |
| tr\|Q5BXD7 | SJCHGC03429 protein (Fragment) | 10.92 | 54,58 | 11 | 2.48 | 44,49 | 2 | NC |
| tr\|Q5D8P1 | SJCHGC06542 protein | 9.97 | 72,78 | 11 | - | - | - | NC |
| tr\|C1LS35 | Ribonuclease T2 | 8.02 | 69,99 | 11 | 9.49 | 56.2 | 11 | SP |
| tr\|Q5D8E5 | SJCHGC09536 protein | 5.32 | 69,99 | 11 | 3.79 | 69,99 | 11 | - |
| tr\|A0A095B2I3 | Thimet oligopeptidase | 3.4 | 37,20 | 11 | - | - | - | - |
| tr\|C1L5R4 | N-acetyl galactosaminidase, alpha | 2.18 | 42,17 | 11 | 0.01 | 26,73 | 3 | SP |
| tr\|A0A183RCJ8 | Uncharacterized protein | 2.03 | 30,84 | 11 | 2.71 | 29,75 | 9 | SP |
| tr\|A0A095BZ75 | Tropinone reductase 2 | 2.01 | 45,03 | 11 | - | - | - | - |
| tr\|C1LHK9 | Phosphotransferase | 2 | 57,64 | 11 | - | - | - | - |
| tr\|A0A183N316 | Uncharacterized protein | 2 | 46,90 | 11 | - | - | - | NC |
| tr\|C1LG71 | Furin-1 | 0.39 | 39,91 | 11 | 0.52 | 35,94 | 5 | SP |
| tr\|A0A094ZT60 | Endoplasmin | 0.13 | 47,17 | 11 | - | - | - | - |
| tr\|Q5D9F5 | SJCHGC05822 protein | 20.24 | 61,77 | 10 | - | - | - | - |
| tr\|Q5DBN7 | SJCHGC09380 protein | 18.91 | 58,57 | 10 | - | - | - | NC |
| tr\|C1LG87 | 3'(2'), 5'-bisphosphate nucleotidase | 18.12 | 58,45 | 10 | - | - | - | SP |
| tr\|C1LIR8 | Aspartate aminotransferase | 17.32 | 52,46 | 10 | 5.97 | 24,62 | 3 | NC |
| tr\|Q5D8R2 | SJCHGC08964 protein (Fragment) | 17.21 | 65,93 | 10 | 12.15 | 38,04 | 6 | - |
| tr\|Q86EQ6 | Clone ZZD1530 mRNA sequence | 16.3 | 75,34 | 10 | 9.53 | 46,11 | 4 | NC |
| tr\|C1LGR1 | AP complex subunit beta | 16.03 | 31,90 | 10 | 1.54 | 15,85 | 2 | NC |
| tr\|Q5DC26 | ADP-ribosylation factor 3 | 16.03 | 82,31 | 10 | 6.82 | 43,65 | 6 | NC |
| tr\|Q5BVV0 | SJCHGC09648 protein (Fragment) | 15.81 | 68,45 | 10 | 7.13 | 42,32 | 3 | NC |
| tr\|Q5DCB4 | SJCHGC05854 protein | 15.52 | 56,38 | 10 | 8.31 | 22,93 | 5 | SP |
| tr\|Q86EQ2 | Clone ZZD1548 mRNA sequence | 15.41 | 74,66 | 10 | 7.96 | 35,85 | 7 | - |
| tr\|A0A183MGF1 | Uncharacterized protein | 15.28 | 26,51 | 10 | - | - | - | - |
| tr\|Q5DGC4 | SJCHGC02082 protein | 14.8 | 79,50 | 10 | 7.39 | 4.34 | 4 | NC |
| tr\|Q26520 | JF-2 protein (Fragment) | 14.71 | 53,36 | 10 | - | - | - | - |
| tr\|Q5DBS4 | SJCHGC05684 protein | 14.7 | 43,61 | 10 | - | - | - | NC |
| tr\|Q5DBK7 | Activator of 90 kDa heat shock protein ATPase homolog 1 | 13.72 | 39,35 | 10 | 8.97 | 3.294 | 5 | - |
| tr\|C1L595 | 2-oxoglutarate dehydrogenase E2 component | 13.56 | 50,67 | 10 | - | - | - | - |
| tr\|C1LNI8 | Putative Retinol dehydrogenase 11 | 13.5 | 70,59 | 10 | - | - | - | NC |
| tr\|Q5DFC5 | SJCHGC06267 protein | 12.99 | 73,97 | 10 | - | - | - | NC |
| tr\|Q5BSG7 | SJCHGC04345 protein (Fragment) | 12.28 | 100 | 10 | 3.12 | 27,03 | 2 | NC |
| tr\|Q5DCC6 | SJCHGC02843 protein | 11.5 | 61,16 | 10 | 4.64 | 24,46 | 4 | - |
| tr\|C7TZA4 | Putative uncharacterized protein | 10.05 | 41,58 | 10 | 10.12 | 4.33 | 12 | SP |
| tr\|Q5DF72 | Histone H2A | 7.37 | 66,42 | 10 | - | - | - | NC |
| tr\|C1LLL3 | Peptidase M8, leishmanolysin,domain-containing protein | 6.96 | 39,78 | 10 | 3.01 | 26,64 | 3 | SP |
| tr\|Q5DFR6 | Aprt protein | 4 | 55,68 | 10 | - | - | - | - |
| tr\|Q5DCI0 | SJCHGC01111 protein | 2 | 57,34 | 10 | - | - | - | - |
| tr\|A0A094ZGB5 | Filamin-A | 0.1 | 28,47 | 10 | 20.62 | 24,65 | 10 | NC |
| tr\|C4QFX9 | Heat shock protein 70 (Hsp70)-4, putative | 0.08 | 16,87 | 10 | 0.11 | 10,39 | 3 | - |
| tr\|Q5DEW6 | SJCHGC09345 protein | 17.96 | 34,72 | 9 | 6.26 | 23,52 | 3 | SP |
| tr\|C1LNT1 | Estrogen-regulated protein EP45 | 16.65 | 34,40 | 9 | 9.46 | 32,17 | 5 | NC |
| tr\|Q86ES4 | Actin depolymerizing factor-like protein | 16.26 | 80,58 | 9 | 8.59 | 65,46 | 5 | NC |
| tr\|Q86EU0 | Histone H2A | 16.06 | 57,05 | 9 | 3.57 | 37,18 | 2 | - |
| tr\|C1LJ48 | Thioredoxin 1 | 15.82 | 62,5 | 9 | - | - | - | - |
| tr\|C1LFS0 | Tektin-2 | 15.53 | 45,89 | 9 | - | - | - | NC |
| tr\|Q5DAH1 | SJCHGC06231 protein | 14.51 | 53,75 | 9 | - | - | - | SP |
| tr\|C1LNG0 | 6-phosphogluconate dehydrogenase, decarboxylating | 13.95 | 46,50 | 9 | 5.01 | 20,78 | 3 | NC |
| tr\|Q5DA91 | Receptor expression-enhancing protein | 12.89 | 35,98 | 9 | - | - | - | NC |
| tr\|Q86EE3 | Clone ZZD485 mRNA sequence | 11.46 | 58,20 | 9 | - | - | - | NC |
| tr\|Q86EG4 | Chloride intracellular channel 4 | 10.99 | 49,05 | 9 | - | - | - | NC |
| tr\|Q5DBC5 | SJCHGC01379 protein | 10.28 | 69,67 | 9 | - | - | - | SP |
| tr\|C1LLK9 | Peptidase M8, leishmanolysin,domain-containing protein | 10.1 | 46,63 | 9 | - | - | - | SP |
| tr\|C1LL90 | Globin-3 | 9.6 | 79,18 | 9 | 8.44 | 72,47 | 7 | - |
| tr\|C1L3P6 | Uncharacterized protein | 9 | 35,80 | 9 | 6.6 | 21,11 | 3 | - |
| tr\|C1LNV0 | 16 kDa calcium-binding protein (Egg antigen SME16) | 7.6 | 79,30 | 9 | 8.29 | 76,55 | 9 | NC |
| tr\|A0A095AHW6 | Putative aminopeptidase W07G4.4 | 2 | 34,58 | 9 | - | - | - | - |
| tr\|C1LRH4 | Calcium/calmodulin-dependent serine protein kinase (MAGUK family) | 1.45 | 31,20 | 9 | 10.54 | 32,91 | 8 | SP |
| tr\|C1LQP0 | Uncharacterized protein | 0.74 | 54,11 | 9 | 1.15 | 5.889 | 10 | SP |
| tr\|A0A183R049 | Uncharacterized protein | 0.28 | 33,39 | 9 | - | - | - | NC |
| tr\|Q5DBP4 | ATP synthase gamma subunit | 15.46 | 88,70 | 8 | - | - | - | - |
| tr\|C1L3V1 | Uroporphyrinogen decarboxylase | 14.4 | 48,08 | 8 | 9.84 | 37,09 | 4 | NC |
| tr\|Q5DBP9 | Complement component 1 Q subcomponent-binding protein, mitochondrial | 14.06 | 63,63 | 8 | - | - | - | SP |
| tr\|Q2LE75 | Cytosolic 5'-nucleotidase II (Fragment) | 13.84 | 35,98 | 8 | - | - | - | NC |
| tr\|Q5DBB7 | SJCHGC06753 protein | 13.66 | 58,27 | 8 | - | - | - | - |
| tr\|O45033 | Calpain (Fragment) | 13.42 | 35,78 | 8 | - | - | - | - |
| tr\|Q5DF24 | SJCHGC03234 protein | 13.36 | 46,72 | 8 | 9.68 | 36,17 | 6 | NC |
| tr\|C1LIE7 | Pyruvate dehydrogenase E1 component, beta | 13.24 | 50,69 | 8 | - | - | - | NC |
| tr\|Q86ET1 | Rab-protein 8 | 12.81 | 66,15 | 8 | 6.37 | 45,96 | 4 | - |
| tr\|Q86DX8 | SJCHGC06009 protein | 12.25 | 61,33 | 8 | - | - | - | NC |
| tr\|Q5DD05 | SJCHGC05876 protein | 12.19 | 33,12 | 8 | 6.28 | 20,43 | 4 | NC |
| tr\|Q86DW4 | SJCHGC01387 protein | 12 | 89,92 | 8 | 10.78 | 88,37 | 6 | NC |
| tr\|Q5DD72 | SJCHGC02571 protein | 11.81 | 22,46 | 8 | - | - | - | - |
| tr\|C1LMN4 | Cytochrome b-c1 complex subunit Rieske, mitochondrial | 11.79 | 62,58 | 8 | - | - | - | NC |
| tr\|C1LLG3 | Histidine triad nucleotide binding protein 1 | 10.01 | 60,90 | 8 | 9.2 | 58,96 | 6 | NC |
| tr\|A0A094ZFT4 | Glycogen debranching enzyme (Fragment) | 9.9 | 22,41 | 8 | 7.53 | 1.008 | 3 | - |
| tr\|C1LRH3 | Calcium/calmodulin-dependent serine protein kinase (MAGUK family) | 9.23 | 28,25 | 8 | 2 | 29,98 | 7 | SP |
| tr\|C1L418 | Hypotherical protein | 9.23 | 44,29 | 8 | 6.51 | 33,53 | 3 | SP |
| tr\|Q5DHK5 | ARMET-like protein | 8.18 | 72,28 | 8 | 3.69 | 51,09 | 4 | SP |
| tr\|Q5BZH7 | SJCHGC08025 protein (Fragment) | 7.81 | 52,96 | 8 | 4.04 | 2.624 | 4 | NC |
| tr\|Q5C0N2 | SJCHGC05656 protein (Fragment) | 7.72 | 44,71 | 8 | - | - | - | SP |
| tr\|C7TZ90 | Putative uncharacterized protein | 6.19 | 28,94 | 8 | 8.38 | 35,26 | 8 | SP |
| tr\|Q5DAC6 | SJCHGC05576 protein | 6 | 25,51 | 8 | 6.01 | 22,63 | 8 | SP |
| tr\|C7TYB5 | Putative uncharacterized protein | 4 | 41,33 | 8 | - | - | - | SP |
| tr\|C1L7D7 | Histidine triad nucleotide binding protein 1 | 0.61 | 60,90 | 8 | - | - | - | NC |
| tr\|Q3KZ47 | SJCHGC06122 protein (Fragment) | 0.15 | 54,64 | 8 | - | - | - | NC |
| tr\|Q86EV4 | Clone ZZD1379 mRNA sequence (Fragment) | 14.01 | 42,23 | 7 | - | - | - | NC |
| tr\|Q5DBK4 | Ng-dimethylarginine dimethylaminohydrolase | 13.66 | 62,72 | 7 | 10.33 | 41,11 | 5 | - |
| tr\|Q86F02 | Alpha-soluble NSF attachment protein | 13.59 | 52,93 | 7 | - | - | - | - |
| tr\|C1L3N1 | Malic enzyme 1, NADP(+)-dependent, cytosolic | 13.15 | 39,35 | 7 | - | - | - | - |
| tr\|Q5DC37 | SJCHGC02147 protein | 12.91 | 38,76 | 7 | - | - | - | SP |
| tr\|Q5BZG5 | SJCHGC05582 protein (Fragment) | 12.65 | 39,57 | 7 | 7.83 | 32,85 | 4 | - |
| tr\|Q5DD24 | SJCHGC09278 protein | 12.48 | 20,91 | 7 | - | - | - | - |
| tr\|Q86F61 | NIF3-like protein 1 | 12.4 | 47,58 | 7 | 8.89 | 39,32 | 6 | - |
| tr\|C1L4E6 | Phosphodiesterase-nucleotide pyrophosphatase | 12.4 | 32,17 | 7 | - | - | - | NC |
| tr\|A0A183LHF7 | Uncharacterized protein | 12.33 | 24,42 | 7 | - | - | - | NC |
| tr\|C1LN65 | Chromobox protein homolog 1 (Heterochromatin protein 1 homolog beta) | 12.19 | 65,97 | 7 | - | - | - | NC |
| tr\|C1L5J3 | Tropomodulin | 11.47 | 61,05 | 7 | - | - | - | - |
| tr\|Q5BYN0 | Sulfhydryl oxidase (Fragment) | 10.45 | 32,71 | 7 | - | - | - | SP |
| tr\|C4N150 | Putative uncharacterized protein | 9.12 | 47,76 | 7 | 5.77 | 28,78 | 3 | - |
| tr\|C7TY94 | Serine/threonine-protein phosphatase | 9.08 | 53,70 | 7 | - | - | - | - |
| tr\|Q86DY5 | Eukaryotic translation initiation factor 5A | 8.57 | 68,75 | 7 | - | - | - | - |
| tr\|C1LIX2 | Putative Thioredoxin, mitochondrial | 8.3 | 80,54 | 7 | 6.28 | 71,13 | 5 | NC |
| tr\|A0A095ARH4 | Adenylosuccinate synthetase | 8.24 | 48,39 | 7 | 8.12 | 38,76 | 5 | NC |
| tr\|Q86FC9 | Clone ZZD252 mRNA sequence | 7.85 | 57,98 | 7 | - | - | - | SP |
| tr\|Q86E40 | Eukaryotic translation initiation factor 6 | 7.73 | 56,37 | 7 | - | - | - | NC |
| tr\|C1LEH2 | Ribonuclease Oy | 7.61 | 29,75 | 7 | 7.67 | 29,75 | 7 | SP |
| tr\|C1LDN5 | Tetraspanin | 6.83 | 20,99 | 7 | 3.65 | 21,45 | 4 | SP |
| tr\|Q5DBB2 | Calycin-like domain-containing protein | 6.75 | 52,27 | 7 | 6.42 | 49,23 | 6 | NC |
| tr\|C1LFL0 | Uncharacterized protein | 6.4 | 51,34 | 7 | 11.04 | 48,12 | 9 | SP |
| tr\|G4V8A4 | Putative short chain dehydrogenase | 4.6 | 41,10 | 7 | - | - | - | NC |
| tr\|C7TXT9 | Putative uncharacterized protein | 4.42 | 36,12 | 7 | 4.92 | 36,12 | 7 | SP |
| tr\|Q5DAL5 | Serine/threonine-protein phosphatase (Fragment) | 4 | 53,07 | 7 | 1.21 | 30,66 | 3 | NC |
| tr\|Q7Z0T6 | Adenosinetriphosphatase | 3.63 | 62,91 | 7 | 3.62 | 62,91 | 6 | - |
| tr\|Q5BSM0 | SJCHGC03807 protein (Fragment) | 3.5 | 86,79 | 7 | - | - | - | NC |
| tr\|Q5C2A1 | SJCHGC04425 protein (Fragment) | 2 | 55,11 | 7 | - | - | - | SP |
| tr\|C1L8N7 | Putative Lysosomal Pro-X carboxypeptidase | 1.28 | 38,76 | 7 | - | - | - | SP |
| tr\|Q5BZV0 | SJCHGC06786 protein (Fragment) | 1.05 | 46,81 | 7 | 0.09 | 59,57 | 6 | SP |
| tr\|C1LQN8 | Uncharacterized protein | 0.22 | 31,79 | 7 | 0.34 | 31,36 | 7 | SP |
| tr\|G4VRR2 | 2-oxoglutarate dehydrogenase | 0.14 | 30,61 | 7 | - | - | - | NC |
| tr\|Q5C489 | SJCHGC02183 protein (Fragment) | 0.05 | 27,64 | 7 | 0.14 | 2.899 | 8 | - |
| tr\|Q5DGS1 | Chromobox protein homolog 1 (Heterochromatin protein 1 homolog beta) | 0.05 | 65,97 | 7 | - | - | - | NC |
| tr\|C1LED9 | NFS1 nitrogen fixation 1 | 12.24 | 30,86 | 6 | - | - | - | - |
| tr\|C1LC25 | Ribosomal protein S3 | 11.52 | 74,34 | 6 | - | - | - | - |
| tr\|A0A183M8I9 | Uncharacterized protein | 11.35 | 41,62 | 6 | - | - | - | - |
| tr\|Q5DGT4 | SJCHGC01067 protein | 11.25 | 51,41 | 6 | - | - | - | - |
| tr\|Q86EU4 | Cytochrome c proximal | 11.21 | 85,18 | 6 | 9.43 | 85,18 | 5 | - |
| tr\|Q5DBY6 | DnaJ (Hsp40) homolog, subfamily A, member 1 | 11.19 | 50 | 6 | - | - | - | - |
| tr\|Q5C7Z7 | SJCHGC06198 protein (Fragment) | 10.95 | 57,52 | 6 | - | - | - | - |
| tr\|C1LGF1 | Phosphorylase kinase, alpha 2 | 10.55 | 64,19 | 6 | - | - | - | - |
| tr\|Q5DI41 | SJCHGC06520 protein | 10.5 | 46,36 | 6 | - | - | - | NC |
| tr\|Q5C6Y9 | SJCHGC08255 protein (Fragment) | 10.48 | 48,71 | 6 | 6.22 | 42,66 | 3 | SP |
| tr\|C1L6V5 | YME1-Like (Mitochondrial Escape) AAA protease | 10.42 | 42,41 | 6 | - | - | - | - |
| tr\|Q5C3Q5 | SJCHGC04071 protein (Fragment) | 10.2 | 47,15 | 6 | - | - | - | NC |
| tr\|Q5C1G7 | SJCHGC05226 protein (Fragment) | 10.16 | 39,03 | 6 | - | - | - | - |
| tr\|Q5C2N1 | SJCHGC03405 protein (Fragment) | 10.12 | 27,09 | 6 | 8.76 | 27,09 | 5 | NC |
| tr\|Q5DFU2 | NADH dehydrogenase (Ubiquinone) 1 beta subcomplex 10 | 9.94 | 49,52 | 6 | - | - | - | - |
| tr\|C1LFS7 | Voltage-dependent anion channel 1 | 9.75 | 35,35 | 6 | - | - | - | - |
| tr\|Q5DFE0 | 40S ribosomal protein S8 | 9.51 | 43,77 | 6 | - | - | - | - |
| tr\|Q5BQX6 | SJCHGC09770 protein | 9.48 | 58,74 | 6 | 10 | 58,74 | 7 | NC |
| tr\|Q5DA50 | Platelet-activating factor acetylhydrolase, isoform Ib, beta subunit | 9.22 | 63,16 | 6 | 2.75 | 26,32 | 2 | NC |
| tr\|C1L4P3 | Tektin-1 | 8.92 | 64,17 | 6 | 3.09 | 42,53 | 3 | NC |
| tr\|C1L6J6 | Hypotherical protein | 8.71 | 25,65 | 6 | - | - | - | SP |
| tr\|C1L6Z8 | Mitochondrial processing peptidase | 8.39 | 27,30 | 6 | - | - | - | - |
| tr\|C1L631 | Quinolinate phosphoribosyltransferase | 8.37 | 50,16 | 6 | - | - | - | NC |
| tr\|Q5DG45 | EH-domain containing protein (Fragment) | 8.15 | 31,79 | 6 | - | - | - | NC |
| tr\|Q5D8L3 | SJCHGC03591 protein | 7.99 | 63,70 | 6 | - | - | - | NC |
| tr\|Q5C2A4 | SJCHGC05238 protein (Fragment) | 7.96 | 46,77 | 6 | 3.52 | 21,38 | 2 | NC |
| tr\|Q5C3S4 | SJCHGC08757 protein (Fragment) | 7.33 | 51,88 | 6 | 7.24 | 28,11 | 4 | NC |
| tr\|Q5DBU4 | SJCHGC07093 protein | 6.86 | 77,06 | 6 | - | - | - | NC |
| tr\|D2JWR5 | cAMP-dependent protein kinase catalytic subunit | 5.81 | 47,71 | 6 | 8.66 | 34,86 | 6 | - |
| tr\|Q86FH5 | Clone ZZZ297 mRNA sequence | 5.76 | 43,16 | 6 | 1.62 | 43,16 | 6 | SP |
| tr\|C1LK26 | Inositol(Myo)-1(Or 4)-monophosphatase 2 | 5.59 | 25,27 | 6 | - | - | - | NC |
| tr\|A0A183LA20 | Uncharacterized protein | 4.1 | 22,61 | 6 | 3.92 | 23,56 | 4 | SP |
| tr\|C7TZ95 | Putative uncharacterized protein | 3.66 | 44,20 | 6 | 8.14 | 44,20 | 6 | SP |
| tr\|Q5C3I8 | SJCHGC05636 protein (Fragment) | 3.34 | 63,63 | 6 | - | - | - | - |
| tr\|C1LUQ7 | Peptidase inhibitor 16 | 2.86 | 47,08 | 6 | - | - | - | SP |
| tr\|Q5D9C3 | ADP-ribosylation factor 1-like protein | 2.66 | 75 | 6 | - | - | - | NC |
| tr\|Q5DE19 | Histone H2A | 2.61 | 47,33 | 6 | - | - | - | - |
| tr\|Q5DE42 | SJCHGC01653 protein | 2 | 65,71 | 6 | 2 | 28,15 | 6 | NC |
| tr\|Q5BX63 | SJCHGC06890 protein (Fragment) | 1.55 | 25 | 6 | 0.95 | 29,01 | 6 | - |
| tr\|C1LLV5 | NADH dehydrogenase (Ubiquinone) 1 beta subcomplex 10 | 0.45 | 51,66 | 6 | - | - | - | - |
| tr\|Q5BVL1 | SJCHGC02702 protein (Fragment) | 0.28 | 64,28 | 6 | - | - | - | - |
| tr\|C1L670 | Tektin-4 | 10.82 | 44,60 | 5 | 7.89 | 35,67 | 4 | NC |
| tr\|C4Q5U3 | Ankyrin 2,3/unc44, putative (Fragment) | 10.3 | 15,98 | 5 | - | - | - | NC |
| tr\|Q5C0R0 | Putative uncharacterized protein | 10.27 | 35,51 | 5 | - | - | - | - |
| tr\|C1LLP3 | Flotillin 1 | 10.09 | 41,08 | 5 | - | - | - | - |
| tr\|A0A183ME64 | Malic enzyme | 10.06 | 26,39 | 5 | - | - | - | NC |
| tr\|Q5DCR4 | RuvB-like helicase | 9.68 | 46,48 | 5 | - | - | - | - |
| tr\|C7TQQ1 | Alpha-centractin (Centractin) | 9.67 | 47,20 | 5 | - | - | - | - |
| tr\|Q5D8S6 | SJCHGC03291 protein | 9.59 | 49,07 | 5 | 4.83 | 34,11 | 2 | SP |
| tr\|Q86BY1 | Proteasome activator PA28 subunit | 9.37 | 51,81 | 5 | - | - | - | NC |
| tr\|C1LD72 | Ectonucleotide pyrophosphatase/phosphodiesterase family member 5 | 9.26 | 34,86 | 5 | 2.68 | 19,30 | 2 | SP |
| tr\|C1L494 | Uncharacterized protein | 9.24 | 48,03 | 5 | - | - | - | SP |
| tr\|A1XA55 | Adenylosuccinate lyase | 9.15 | 43,75 | 5 | - | - | - | - |
| tr\|A0A183ME55 | Uncharacterized protein | 9.12 | 27,23 | 5 | 3.4 | 22,92 | 2 | - |
| tr\|C1LM56 | Uncharacterized protein | 8.7 | 47,36 | 5 | - | - | - | SP |
| tr\|Q7Z1I6 | Cathepsin | 8.58 | 43,68 | 5 | 4.29 | 25,29 | 2 | SP |
| tr\|C1LFT7 | Leucine-rich repeat-containing protein 57 | 8.44 | 45,44 | 5 | - | - | - | NC |
| tr\|Q5C1E9 | SJCHGC09040 protein (Fragment) | 8.42 | 46,86 | 5 | - | - | - | NC |
| tr\|C9W186 | Lysophospholipase II | 8.3 | 64,75 | 5 | - | - | - | NC |
| tr\|C1LIE0 | LAMA-like protein 2 | 8.14 | 26,12 | 5 | - | - | - | NC |
| tr\|C1LIQ6 | Ubiquitin-activating enzyme E1 | 8.1 | 21,82 | 5 | 7.35 | 23,16 | 5 | NC |
| tr\|C1L4F7 | Microtubule-associated protein | 8.06 | 21,55 | 5 | - | - | - | NC |
| tr\|Q5C6L1 | Galectin (Fragment) | 8.01 | 65,31 | 5 | - | - | - | NC |
| tr\|C1L4W2 | Guanylate kinase | 8 | 24,87 | 5 | 8 | 28,85 | 5 | NC |
| tr\|G4VGB4 | Putative coatomer beta subunit | 8 | 26,37 | 5 | - | - | - | - |
| tr\|Q86ET9 | Ribosomal protein S8 | 7.98 | 33,00 | 5 | - | - | - | NC |
| tr\|Q5BYZ1 | SJCHGC01349 protein (Fragment) | 7.89 | 39,46 | 5 | - | - | - | NC |
| tr\|Q5DA66 | SJCHGC01925 protein | 7.81 | 49,29 | 5 | - | - | - | SP |
| tr\|A0A095AYT1 | Neurogenic locus notch protein 3 (Fragment) | 7.75 | 17,56 | 5 | - | - | - | - |
| tr\|C1L7H2 | Alpha subunit of casein kinase II | 7.71 | 47,26 | 5 | - | - | - | NC |
| tr\|Q5DEM7 | Dolichyl-diphosphooligosaccharide--protein glycosyltransferase subunit 1 | 7.7 | 31,83 | 5 | - | - | - | SP |
| tr\|Q5DBC6 | SJCHGC09313 protein | 7.53 | 55,64 | 5 | 9.24 | 51,38 | 4 | NC |
| tr\|C7TZX9 | Ribonuclease Oy (Fragment) | 7.52 | 21,43 | 5 | 8.53 | 21,43 | 5 | SP |
| tr\|Q8MUK6 | MA | 7.44 | 54,22 | 5 | - | - | - | NC |
| tr\|Q86F68 | Clone ZZD1069 mRNA sequence | 7.41 | 37,63 | 5 | - | - | - | - |
| tr\|Q5DAH2 | Prefoldin subunit 3 | 7.4 | 44,44 | 5 | - | - | - | NC |
| tr\|Q5DAN9 | Glycerol-3-phosphate dehydrogenase [NAD(+)] | 7.34 | 29,06 | 5 | 4.54 | 26,78 | 3 | - |
| tr\|Q86ER2 | Clone ZZD1514 mRNA sequence | 7.34 | 50 | 5 | 2 | 22,90 | 2 | NC |
| tr\|A0A183KBW0 | Pyruvate kinase | 7.31 | 13,01 | 5 | - | - | - | - |
| tr\|Q86F56 | SJCHGC00851 protein (Fragment) | 7.29 | 49,25 | 5 | 7.89 | 40,88 | 5 | SP |
| tr\|Q5DAF0 | Heterogeneous nuclear ribonucleoprotein A1, A2/B1 homolog | 7.27 | 43,68 | 5 | 2.88 | 14,32 | 2 | - |
| tr\|C1LUS4 | 40S ribosomal protein S6 | 6.96 | 49,16 | 5 | - | - | - | NC |
| tr\|C1LFB1 | Coatomer subunit delta | 6.87 | 27,30 | 5 | 1.81 | 17,66 | 2 | - |
| tr\|Q86FE7 | Clone ZZD1469 mRNA sequence | 6.82 | 38,10 | 5 | 5.88 | 32,73 | 4 | - |
| tr\|Q5DDA0 | Adenylyl cyclase-associated protein | 6.76 | 31,92 | 5 | - | - | - | - |
| tr\|C7TQV2 | Hypotheticial protein | 6.58 | 58,50 | 5 | - | - | - | SP |
| tr\|Q5DHY8 | Epididymal secretory protein E1 (Niemann Pick type C2 protein homolog) | 6.47 | 56,16 | 5 | 2.15 | 41,78 | 2 | SP |
| tr\|C1LHZ8 | Uncharacterized protein | 6.43 | 43,84 | 5 | - | - | - | - |
| tr\|Q86EI5 | Clone ZZD336 mRNA sequence | 6.42 | 70,56 | 5 | 5.83 | 64,99 | 5 | - |
| tr\|Q86FD7 | Clone ZZD1560 mRNA sequence | 6.31 | 46,05 | 5 | - | - | - | SP |
| tr\|Q86F89 | Clone ZZZ65 mRNA sequence | 6.29 | 30,09 | 5 | 3.45 | 18,93 | 2 | SP |
| tr\|Q86RB3 | 21.7 kDa protein | 6.29 | 50,26 | 5 | 4.72 | 43,23 | 3 | NC |
| tr\|Q86ET8 | SJCHGC04696 protein (Fragment) | 6.28 | 58,75 | 5 | - | - | - | NC |
| tr\|Q5DH98 | SJCHGC05498 protein | 6.25 | 52,24 | 5 | - | - | - | NC |
| tr\|Q86EN9 | 60S acidic ribosomal protein P0 | 6.1 | 34,20 | 5 | - | - | - | - |
| tr\|C1LCT0 | Phosphatase 2A inhibitor I2PP2A | 6.02 | 47,20 | 5 | - | - | - | NC |
| tr\|A0A095A2F7 | Phosphoglucomutase-1 | 6.01 | 17,73 | 5 | 4.37 | 15,47 | 2 | NC |
| tr\|Q5DG13 | GrpE protein homolog | 5.93 | 70,50 | 5 | - | - | - | NC |
| tr\|C1L8G6 | RAS-like GTP-binding protein | 5.91 | 34,38 | 5 | - | - | - | NC |
| tr\|Q5DEE1 | SJCHGC09190 protein | 5.62 | 37,40 | 5 | 4.38 | 21,53 | 4 | SP |
| tr\|Q5DFB5 | Ribosomal protein L10a | 5.21 | 35,01 | 5 | - | - | - | - |
| tr\|Q5C2D3 | SJCHGC09008 protein (Fragment) | 5.12 | 33,32 | 5 | - | - | - | - |
| tr\|Q5DDA2 | SJCHGC05516 protein | 5.1 | 49,47 | 5 | 5.17 | 37,88 | 4 | NC |
| tr\|Q5D8W0 | SJCHGC06710 protein | 4.55 | 48,71 | 5 | 1.4 | 36,89 | 2 | SP |
| tr\|Q5DCX5 | SJCHGC04892 protein | 3.71 | 36,00 | 5 | 4.26 | 36,00 | 5 | NC |
| tr\|Q5DGA4 | SJCHGC09069 protein | 3.29 | 61,33 | 5 | - | - | - | - |
| tr\|Q86EC7 | Clone ZZD528 mRNA sequence | 2.67 | 30,14 | 5 | - | - | - | - |
| tr\|C1LRH0 | Uncharacterized protein | 2.34 | 23,28 | 5 | 2.57 | 32,42 | 5 | SP |
| tr\|Q5DGN7 | Rab-protein 8 | 2.26 | 46,34 | 5 | - | - | - | NC |
| tr\|G4VIQ1 | Putative mannosyl-oligosaccharide glucosidase | 2.22 | 20,11 | 5 | - | - | - | SP |
| tr\|A0A183QAA5 | Uncharacterized protein | 2.09 | 43,09 | 5 | 2.19 | 39,23 | 5 | - |
| tr\|Q5DGB7 | SJCHGC03910 protein | 2.02 | 43,77 | 5 | - | - | - | NC |
| tr\|Q86QQ1 | IR2 (Fragment) | 1.56 | 38,96 | 5 | 9.46 | 31,16 | 5 | - |
| tr\|C7TZY8 | Ribonuclease Oy (Fragment) | 1.03 | 25,74 | 5 | 1.34 | 25,74 | 5 | SP |
| tr\|Q86DY7 | 40S ribosomal protein S6 | 0.71 | 39,68 | 5 | - | - | - | - |
| tr\|C1L6L8 | Tissue specific transplantation antigen P35B | 8.75 | 48,73 | 4 | - | - | - | - |
| tr\|C1LFG5 | NADH:ubiquinone reductase 42kD subunit precurs | 8.61 | 33,97 | 4 | - | - | - | NC |
| tr\|Q5DCX4 | Ferritin | 8.55 | 75,70 | 4 | - | - | - | - |
| tr\|Q5DDJ6 | SJCHGC06773 protein | 8.5 | 29,35 | 4 | 3.85 | 14,67 | 2 | - |
| tr\|Q5DEQ9 | Coatomer subunit epsilon | 8.3 | 61,15 | 4 | - | - | - | NC |
| tr\|Q5DGG1 | SJCHGC09117 protein | 8.12 | 25,90 | 4 | 4.23 | 22,56 | 2 | SP |
| tr\|C1L8C8 | Small subunit ribosomal protein S2e | 8.05 | 59,61 | 4 | - | - | - | NC |
| tr\|Q5DDX5 | Ribosomal protein L18 | 7.93 | 48,93 | 4 | 5.32 | 56,91 | 3 | - |
| tr\|Q5DE52 | CG3195-PC, isoform C | 7.87 | 60,00 | 4 | - | - | - | NC |
| tr\|Q5DDQ5 | SJCHGC01481 protein | 7.85 | 30,43 | 4 | - | - | - | NC |
| tr\|A0A183JLT6 | GTP-binding nuclear protein | 7.81 | 56,01 | 4 | 5.65 | 50,45 | 3 | NC |
| tr\|Q5C5L3 | SJCHGC06471 protein (Fragment) | 7.77 | 55,79 | 4 | - | - | - | NC |
| tr\|Q5BXQ1 | SJCHGC07586 protein (Fragment) | 7.7 | 55,11 | 4 | - | - | - | - |
| tr\|Q5D9E8 | SJCHGC01901 protein | 7.64 | 58,99 | 4 | - | - | - | - |
| tr\|A0A183JDH3 | Delta-aminolevulinic acid dehydratase | 7.61 | 58,70 | 4 | 4.32 | 28,25 | 2 | NC |
| tr\|G4VT44 | Putative calmodulin | 7.56 | 73,76 | 4 | 6.66 | 65,57 | 4 | NC |
| tr\|Q5DCQ0 | SJCHGC05391 protein | 7.48 | 35,67 | 4 | 2.28 | 14,11 | 2 | NC |
| tr\|Q5DFU4 | Actin-related protein 2/3 complex subunit 4 | 7.43 | 57,56 | 4 | 6 | 28,49 | 3 | - |
| tr\|Q5DAM8 | RuvB-like helicase | 7.32 | 17,98 | 4 | - | - | - | - |
| tr\|Q5DC34 | SJCHGC01809 protein | 7.24 | 18,62 | 4 | - | - | - | - |
| tr\|C1LKU2 | Erythrocyte band 7 integral membrane protein | 7.12 | 46,27 | 4 | - | - | - | NC |
| tr\|Q5DC03 | Hypotheticial protein | 7.09 | 28,70 | 4 | 2.28 | 20,82 | 2 | SP |
| tr\|Q5C1F2 | SJCHGC07617 protein (Fragment) | 6.9 | 50 | 4 | 6.35 | 39,66 | 5 | NC |
| tr\|C1LEE6 | Putative GMP synthase | 6.88 | 23,95 | 4 | - | - | - | NC |
| tr\|C1LIF1 | Peptidyl--hydroxyglycine--amidating lyase | 6.86 | 37,52 | 4 | 8.1 | 2.494 | 4 | SP |
| tr\|C1LFY3 | Isocitrate dehydrogenase (NAD+) | 6.78 | 51,20 | 4 | - | - | - | - |
| tr\|C1LEY0 | Spermatogenesis-associated protein 6 | 6.75 | 31,67 | 4 | 7.23 | 36,62 | 4 | NC |
| tr\|Q5DHC5 | Putative Prefoldin subunit 2 | 6.61 | 48,33 | 4 | - | - | - | NC |
| tr\|C1L8J7 | 40S ribosomal protein S3a | 6.51 | 47,49 | 4 | - | - | - | NC |
| tr\|C1LYI9 | Calcium-binding EF-hand,domain-containing protein | 6.43 | 84,50 | 4 | 6.68 | 84,50 | 4 | NC |
| tr\|C1LFR2 | Dentin sialophosphoprotein | 6.39 | 9,070 | 4 | 5.87 | 90,70 | 4 | SP |
| tr\|G4LZC4 | Linker histone H1 | 6.38 | 47,33 | 4 | - | - | - | - |
| tr\|C1LI59 | Transmembrane emp24 domain-containing protein 10 | 6.36 | 37,38 | 4 | - | - | - | SP |
| tr\|Q5DFX8 | Pyruvate dehydrogenase E1 component alpha | 6.28 | 37,76 | 4 | - | - | - | NC |
| tr\|Q5DFB6 | NADH dehydrogenase [ubiquinone] 1 alpha subcomplex subunit 8 | 6.28 | 48,87 | 4 | - | - | - | NC |
| tr\|Q5C2V0 | SJCHGC06560 protein (Fragment) | 6.11 | 52,97 | 4 | - | - | - | NC |
| tr\|C1LQQ5 | Mitochondrial import receptor subunit TOM34 | 6.02 | 34,43 | 4 | - | - | - | NC |
| tr\|C1LA91 | Translationally-controlled tumor homolog | 5.98 | 46,75 | 4 | 6.8 | 43,79 | 4 | NC |
| tr\|C1LQR4 | Major egg antigen (P40) | 5.87 | 21,89 | 4 | 4.5 | 30,55 | 3 | NC |
| tr\|Q5D8J6 | SJCHGC06034 protein | 5.65 | 27,55 | 4 | - | - | - | - |
| tr\|C1LG51 | Uncharacterized protein | 5.58 | 48,28 | 4 | - | - | - | - |
| tr\|Q86F50 | Clone ZZD1110 mRNA sequence | 5.41 | 24,04 | 4 | - | - | - | - |
| tr\|Q5DFC1 | Dolichyl-diphosphooligosaccharide--protein glycosyltransferase 48 kDa subunit | 5.36 | 39,48 | 4 | - | - | - | SP |
| tr\|Q3KZ44 | SJCHGC09365 protein | 5.35 | 35,73 | 4 | - | - | - | - |
| tr\|C1LG22 | Coenzyme Q6 homolog, monooxygenase | 5.25 | 19,91 | 4 | - | - | - | - |
| tr\|Q5DHS3 | Phosphomannomutase | 5.15 | 57,56 | 4 | 3.21 | 35,12 | 2 | NC |
| tr\|Q5DD54 | Guanine nucleotide-binding protein subunit beta 2-like 1 | 4.98 | 31,52 | 4 | - | - | - | - |
| tr\|Q5C2T7 | SJCHGC08721 protein (Fragment) | 4.95 | 48,44 | 4 | - | - | - | NC |
| tr\|Q5DAD4 | Eukaryotic translation initiation factor 3 subunit D | 4.88 | 16,35 | 4 | - | - | - | - |
| tr\|Q5D918 | SJCHGC06659 protein | 4.79 | 36,77 | 4 | - | - | - | NC |
| tr\|F0UXG3 | Cystatin-B (Stefin-B) | 4.72 | 76,23 | 4 | 4.09 | 41,58 | 3 | - |
| tr\|Q5C0R7 | SJCHGC03323 protein (Fragment) | 4.66 | 43,04 | 4 | 3.28 | 14,76 | 3 | NC |
| tr\|C1LIE3 | Thioredoxin-like 2 | 4.41 | 49,30 | 4 | 3.01 | 37,67 | 3 | NC |
| tr\|C1LH37 | Putative iron-dependent peroxidase | 4.38 | 22,34 | 4 | 5.33 | 29,89 | 3 | SP |
| tr\|Q5C146 | SJCHGC05925 protein (Fragment) | 4.38 | 17,74 | 4 | - | - | - | SP |
| tr\|C1LLZ5 | Ubiquitin carboxyl-terminal hydrolase | 4.37 | 31,34 | 4 | - | - | - | NC |
| tr\|Q5DA58 | SJCHGC02117 protein | 4 | 10,83 | 4 | 4.54 | 93,41 | 4 | SP |
| tr\|Q5C317 | SJCHGC01097 protein (Fragment) | 3.92 | 59,06 | 4 | - | - | - | - |
| tr\|C1LHZ2 | ADP-ribosylation factor 4 | 3.9 | 60,00 | 4 | 3.05 | 40,00 | 3 | NC |
| tr\|Q5DHL4 | SJCHGC06792 protein | 3.56 | 49,54 | 4 | 3.36 | 49,54 | 3 | SP |
| tr\|C1LVZ5 | Tetraspanin | 3.53 | 20,18 | 4 | - | - | - | SP |
| tr\|G4M1L1 | Vesicular amine transporter, putative | 3.4 | 16,75 | 4 | - | - | - | SP |
| tr\|Q5BX10 | SJCHGC04602 protein (Fragment) | 3.19 | 35,64 | 4 | - | - | - | NC |
| tr\|Q5BXH9 | SJCHGC04970 protein (Fragment) | 3.1 | 21,09 | 4 | - | - | - | SP |
| tr\|Q5C2D4 | SJCHGC07107 protein (Fragment) | 2.01 | 41,42 | 4 | 2.58 | 31,90 | 2 | - |
| tr\|A0A183N4U7 | Uncharacterized protein | 2 | 38,15 | 4 | - | - | - | NC |
| tr\|G4V664 | Similar to TatD DNAse domain containing 1 | 2 | 23,00 | 4 | - | - | - | NC |
| tr\|C1LKU5 | Erythrocyte band 7 integral membrane protein | 1.58 | 31,63 | 4 | - | - | - | NC |
| tr\|Q5DFB9 | SJCHGC01915 protein | 1.44 | 40,66 | 4 | - | - | - | SP |
| tr\|A0A183MS33 | Uncharacterized protein | 1.18 | 12,44 | 4 | - | - | - | NC |
| tr\|B1WA71 | Dynein light chain LC6, flagellar outer arm | 0.68 | 67,03 | 4 | - | - | - | NC |
| tr\|C1LRG8 | Uncharacterized protein | 0.37 | 24,54 | 4 | 0.75 | 38,64 | 5 | SP |
| tr\|C1LQN4 | Uncharacterized protein | 0.16 | 39,84 | 4 | 0.3 | 44,06 | 5 | SP |
| tr\|Q86EN8 | Clone ZZD1582 mRNA sequence | 0.1 | 35,35 | 4 | - | - | - | - |
| tr\|G4V5U5 | Putative macroglobulin/complement | 0.07 | 15,15 | 4 | 0.28 | 10,81 | 2 | SP |
| tr\|C8CHI6 | Methionine aminopeptidase 2 | 6.74 | 29,80 | 3 | - | - | - | - |
| tr\|Q86DV7 | Clone ZZZ66 mRNA sequence | 6.44 | 38,56 | 3 | - | - | - | - |
| tr\|Q5C1F6 | SJCHGC05675 protein (Fragment) | 6.37 | 27,41 | 3 | - | - | - | NC |
| tr\|Q5DAR0 | SJCHGC04873 protein | 6.32 | 47,04 | 3 | - | - | - | NC |
| tr\|Q5D9K2 | SJCHGC09130 protein | 6.27 | 30,25 | 3 | 4.81 | 22,81 | 2 | NC |
| tr\|C1LED7 | ATP-dependent 6-phosphofructokinase | 6.23 | 26,84 | 3 | - | - | - | - |
| tr\|C1LIQ7 | Myophilin | 6.18 | 34,25 | 3 | 0.2 | 21,92 | 2 | - |
| tr\|Q5BTG2 | SJCHGC01414 protein (Fragment) | 6.11 | 60,82 | 3 | - | - | - | NC |
| tr\|A0A095CE06 | Alpha-2-macroglobulin-like protein 1 | 6.1 | 12,52 | 3 | 4.02 | 98,71 | 2 | - |
| tr\|Q5C0P0 | SJCHGC05057 protein (Fragment) | 6.08 | 30,29 | 3 | - | - | - | SP |
| tr\|A0A183NX35 | Uncharacterized protein | 6.02 | 25,22 | 3 | - | - | - | NC |
| tr\|C1LG41 | Protein FAM82B | 6.01 | 14,65 | 3 | - | - | - | - |
| tr\|Q5C4Q0 | SJCHGC08461 protein (Fragment) | 6 | 30,43 | 3 | - | - | - | NC |
| tr\|A0A183RJG7 | Uncharacterized protein | 6 | 41,67 | 3 | 3.03 | 28,33 | 2 | NC |
| tr\|C1LS91 | 40S ribosomal protein SA | 5.98 | 37,99 | 3 | 2.87 | 16,85 | 2 | NC |
| tr\|Q5DBM0 | Annexin | 5.98 | 24,28 | 3 | 6 | 20,34 | 3 | - |
| tr\|Q5DCQ5 | SJCHGC06657 protein | 5.98 | 38,96 | 3 | 4.3 | 34,27 | 2 | - |
| tr\|C1LE88 | Structure specific recognition protein 1 | 5.77 | 15,45 | 3 | - | - | - | - |
| tr\|C1LF96 | Ethanolamine kinase 1 | 5.61 | 27,41 | 3 | 4.09 | 29,37 | 2 | - |
| tr\|C1LD93 | Charged multivesicular body protein 1a (Chromatin-modifying protein 1a) | 5.58 | 43,83 | 3 | - | - | - | NC |
| tr\|C1LJ93 | Eukaryotic translation initiation factor 3 subunit B | 5.5 | 27,07 | 3 | - | - | - | - |
| tr\|Q5C4P1 | SJCHGC05029 protein (Fragment) | 5.45 | 25,24 | 3 | - | - | - | SP |
| tr\|Q5BWB2 | SJCHGC04979 protein (Fragment) | 5.42 | 31,58 | 3 | - | - | - | NC |
| tr\|Q5DC50 | Sulfhydryl oxidase | 5.33 | 69,63 | 3 | - | - | - | NC |
| tr\|C1L4Y2 | Heterogeneous nuclear ribonucleoprotein U-like protein 1 | 5.19 | 16,94 | 3 | - | - | - | NC |
| tr\|C1L3V5 | Procollagen-lysine, 2-oxoglutarate 5-dioxygenase 3 | 5.16 | 13,31 | 3 | - | - | - | SP |
| tr\|Q5D948 | Malignant T cell amplified sequence 1 (Fragment) | 5.13 | 47,79 | 3 | - | - | - | - |
| tr\|G4V9M4 | Neurotracting/lsamp/neurotrimin/obcam related cell adhesion molecule | 5.12 | 17,52 | 3 | - | - | - | - |
| tr\|C1LH78 | Actin-like protein | 5.06 | 28,85 | 3 | 5.02 | 31,54 | 3 | - |
| tr\|Q5BXX9 | SJCHGC04834 protein (Fragment) | 5.05 | 32,96 | 3 | - | - | - | SP |
| tr\|C1LMR7 | Putative Cofilin-1 | 5.04 | 46,66 | 3 | - | - | - | - |
| tr\|Q86EP8 | Clone ZZD1555 mRNA sequence | 5.01 | 30,09 | 3 | - | - | - | NC |
| tr\|Q86F66 | Clone ZZD1070 mRNA sequence | 4.89 | 18,79 | 3 | - | - | - | NC |
| tr\|G4VMJ5 | Putative 60s ribosomal protein L11 | 4.86 | 39,44 | 3 | - | - | - | - |
| tr\|C7TYY1 | Somula protein | 4.82 | 41,26 | 3 | 2.02 | 39,80 | 2 | SP |
| tr\|G4M0F6 | Macrophage scavenger receptor-related | 4.79 | 11,86 | 3 | 3.4 | 12,56 | 2 | NC |
| tr\|Q5BS55 | Barrier-to-autointegration factor | 4.77 | 57,77 | 3 | - | - | - | - |
| tr\|A0A183ME52 | 40S ribosomal protein S4 | 4.76 | 45,48 | 3 | - | - | - | NC |
| tr\|A0A183MRB7 | Uncharacterized protein | 4.74 | 23,10 | 3 | - | - | - | NC |
| tr\|A4GU99 | RNA-binding protein 8A | 4.74 | 39,19 | 3 | 2.14 | 13,07 | 2 | - |
| tr\|A0A094ZG81 | Small nuclear ribonucleoprotein Sm D3 | 4.73 | 34,02 | 3 | - | - | - | - |
| tr\|C1LJX0 | UV excision repair protein RAD23 homolog B | 4.59 | 34,29 | 3 | - | - | - | NC |
| tr\|G4VN72 | Putative tropomyosin | 4.53 | 50,34 | 3 | 3.77 | 26,75 | 2 | NC |
| tr\|Q5DDM1 | Polymerase (RNA) II (DNA directed) polypeptide E | 4.48 | 45,62 | 3 | - | - | - | - |
| tr\|Q5DI27 | SJCHGC06484 protein | 4.42 | 30,32 | 3 | - | - | - | SP |
| tr\|C1LF00 | Glutaminyl-peptide cyclotransferase | 4.33 | 22,31 | 3 | - | - | - | SP |
| tr\|Q5BY91 | SJCHGC01769 protein (Fragment) | 4.29 | 36,88 | 3 | - | - | - | - |
| tr\|Q5C3Q8 | SJCHGC02377 protein (Fragment) | 4.24 | 24,17 | 3 | - | - | - | NC |
| tr\|C1LNG4 | Uncharacterized protein | 4.24 | 37,92 | 3 | 0.75 | 34,97 | 3 | SP |
| tr\|Q5D9P3 | Calcyphosin-like protein | 4.22 | 21,15 | 3 | - | - | - | - |
| tr\|A0A183LMH8 | Uncharacterized protein | 4.17 | 27,03 | 3 | - | - | - | - |
| tr\|Q5C3C2 | SJCHGC05634 protein (Fragment) | 4.15 | 21,00 | 3 | 3.3 | 20,22 | 2 | NC |
| tr\|C1LUF5 | ATP:ADP antiporter | 4.14 | 42,77 | 3 | - | - | - | - |
| tr\|G4VTQ8 | Putative 40s ribosomal protein S9 | 4.09 | 46,34 | 3 | - | - | - | - |
| tr\|Q5DF09 | SJCHGC02626 protein | 4.03 | 31,63 | 3 | 4.04 | 29,24 | 3 | NC |
| tr\|Q5DDH1 | SJCHGC02110 protein | 4.03 | 56,55 | 3 | - | - | - | NC |
| tr\|C1LNL3 | Eukaryotic initiation factor 4A | 4.02 | 41,58 | 3 | - | - | - | NC |
| tr\|A0A183KXZ0 | Uncharacterized protein | 4.02 | 46,12 | 3 | - | - | - | NC |
| tr\|A0A095BWP6 | DNA helicase | 4.01 | 12,68 | 3 | 0 | 10,23 | 3 | - |
| tr\|C1LDX0 | Hypotheticial protein | 4 | 12,72 | 3 | 3.89 | 15,71 | 2 | SP |
| tr\|C4QCA2 | Ribonucleoside-diphosphate reductase small chain, putative | 4 | 33,41 | 3 | - | - | - | NC |
| tr\|Q5DHQ5 | Signal peptidase complex catalytic subunit SEC11 | 3.95 | 51,91 | 3 | - | - | - | NC |
| tr\|G4VNT1 | Putative coatomer alpha subunit | 3.89 | 15,09 | 3 | - | - | - | NC |
| tr\|Q5BQW6 | SJCHGC09783 protein | 3.88 | 49,41 | 3 | - | - | - | NC |
| tr\|G4VFX7 | Putative histidyl-tRNA synthetase | 3.86 | 34,33 | 3 | - | - | - | - |
| tr\|Q5DBT0 | Cytochrome C Oxidase | 3.53 | 80,66 | 3 | - | - | - | NC |
| tr\|C1L4S3 | Quinoid DihyroPteridine Reductase | 3.52 | 29,66 | 3 | - | - | - | NC |
| tr\|Q5BXT2 | SJCHGC08765 protein (Fragment) | 3.47 | 18,88 | 3 | - | - | - | - |
| tr\|Q5DBU7 | SJCHGC06734 protein | 3.32 | 47,09 | 3 | - | - | - | NC |
| tr\|C1LEI6 | Uncharacterized protein | 3.32 | 9,262 | 3 | 4.06 | 13,42 | 3 | - |
| tr\|Q5BZ59 | SJCHGC04070 protein (Fragment) | 3.27 | 37,13 | 3 | 3.78 | 2.375 | 3 | - |
| tr\|A0A183KCS0 | tRNA-splicing ligase RtcB homolog | 3.25 | 8,107 | 3 | - | - | - | NC |
| tr\|Q5DHE4 | SJCHGC04453 protein | 3.1 | 47,09 | 3 | 2.62 | 27,09 | 2 | NC |
| tr\|A0A183R1G4 | Uncharacterized protein | 3.02 | 20,98 | 3 | - | - | - | - |
| tr\|Q5D9G0 | SJCHGC08686 protein (Fragment) | 2.98 | 32,24 | 3 | - | - | - | - |
| tr\|O01372 | 22.6kDa membrane-associated antigen | 2.98 | 38,74 | 3 | - | - | - | NC |
| tr\|A0A183QSF2 | Uncharacterized protein | 2.97 | 37,20 | 3 | - | - | - | - |
| tr\|A0A183PGY4 | Uncharacterized protein | 2.87 | 47,88 | 3 | - | - | - | NC |
| tr\|Q86E14 | Gamma-aminobutyric acid receptor-associated protein-like 2 | 2.65 | 34,45 | 3 | 2.68 | 45,37 | 2 | - |
| tr\|Q86FE2 | SJCHGC01749 protein | 2.63 | 35,69 | 3 | - | - | - | SP |
| tr\|C1L4C4 | Glia maturation factor beta | 2.45 | 55,00 | 3 | - | - | - | NC |
| tr\|Q5C7Y0 | SJCHGC09066 protein (Fragment) | 2.25 | 33,86 | 3 | - | - | - | NC |
| tr\|A0A094ZUT6 | Copine-8 | 2.14 | 8,450 | 3 | - | - | - | NC |
| tr\|Q5BY50 | SJCHGC05695 protein (Fragment) | 2.09 | 28,76 | 3 | - | - | - | NC |
| tr\|A0A094ZHV1 | Isocitrate dehydrogenase [NAD] subunit, mitochondrial (Fragment) | 2.01 | 35,28 | 3 | - | - | - | NC |
| tr\|Q5C6G9 | SJCHGC06148 protein (Fragment) | 1.96 | 22,05 | 3 | - | - | - | SP |
| tr\|A0A094ZDN0 | AP-2 complex subunit alpha | 1.66 | 17,33 | 3 | - | - | - | NC |
| tr\|Q86FA1 | Clone ZZZ329 mRNA sequence | 1.62 | 25,60 | 3 | - | - | - | SP |
| tr\|Q5BXR1 | SJCHGC05267 protein (Fragment) | 1.22 | 54,43 | 3 | - | - | - | NC |
| tr\|C1LYM5 | Calcium-binding EF-hand,domain-containing protein | 0.96 | 78,86 | 3 | 1.23 | 78,86 | 3 | NC |
| tr\|Q5DAM5 | SJCHGC00999 protein | 0.77 | 38,51 | 3 | - | - | - | NC |
| tr\|Q5BWB6 | SJCHGC03871 protein (Fragment) | 0.61 | 23,63 | 3 | - | - | - | - |
| tr\|C1LIQ9 | Myophilin | 0.57 | 29,67 | 3 | 4.96 | 2.967 | 3 | - |
| tr\|Q5DAS0 | Actin-related protein 2/3 complex subunit 3 | 0.36 | 68,51 | 3 | - | - | - | NC |
| tr\|C7TZS1 | Adenylyl cyclase-associated protein (Fragment) | 0.3 | 33,12 | 3 | - | - | - | - |
| tr\|G4VDC3 | Putative uncharacterized protein | 0.29 | 19,07 | 3 | - | - | - | NC |
| tr\|Q26554 | Rab-related GTP-binding protein | 0.18 | 31,22 | 3 | - | - | - | - |
| tr\|Q5DAB8 | SJCHGC02859 protein | 0.16 | 31,65 | 3 | - | - | - | NC |
| tr\|Q5BXQ9 | SJCHGC06980 protein (Fragment) | 0.06 | 23,54 | 3 | - | - | - | - |
| tr\|C7TY45 | Thioredoxin domain-containing protein 4 | 4.63 | 28,81 | 2 | - | - | - | SP |
| tr\|C1LJ60 | Putative 26S proteasome non-ATPase regulatory subunit 11 | 4.59 | 33,57 | 2 | - | - | - | - |
| tr\|Q5DCP8 | Prefoldin subunit 4 | 4.57 | 45,08 | 2 | - | - | - | NC |
| tr\|Q86EP9 | Ribosomal protein S5a | 4.52 | 62,37 | 2 | - | - | - | NC |
| tr\|C1LF55 | Uncharacterized protein | 4.44 | 31,42 | 2 | - | - | - | - |
| tr\|C1L3Q7 | Pyridoxal (Pyridoxine, vitamin B6) kinase | 4.35 | 47,45 | 2 | - | - | - | SP |
| tr\|Q5DED2 | SJCHGC02251 protein | 4.16 | 21,25 | 2 | 4.28 | 21,25 | 2 | NC |
| tr\|Q5C246 | SJCHGC05125 protein (Fragment) | 4.16 | 44,26 | 2 | - | - | - | NC |
| tr\|Q5BR29 | SJCHGC09710 protein | 4.14 | 45,15 | 2 | - | - | - | NC |
| tr\|Q5DES5 | Translocon-associated protein subunit beta | 4.14 | 26,66 | 2 | - | - | - | SP |
| tr\|Q5DCB3 | Protein-L-isoaspartate O-methyltransferase | 4.12 | 55,00 | 2 | - | - | - | NC |
| tr\|Q5DFG9 | Putative uncharacterized protein | 4.11 | 32,74 | 2 | - | - | - | SP |
| tr\|A0A183NWL0 | Uncharacterized protein | 4.1 | 34,90 | 2 | - | - | - | NC |
| tr\|Q5DGZ0 | F-actin capping protein subunit beta | 4.03 | 26,35 | 2 | - | - | - | NC |
| tr\|C1LVM1 | Ribosomal protein L9 | 4.03 | 48,91 | 2 | - | - | - | - |
| tr\|A0A183QG72 | Uncharacterized protein | 4.03 | 26,98 | 2 | - | - | - | - |
| tr\|A0A095BTU9 | Actin-related protein 2-A | 4.02 | 22,84 | 2 | - | - | - | - |
| tr\|A0A183LAM0 | Uncharacterized protein | 4.02 | 17,08 | 2 | - | - | - | NC |
| tr\|Q86F25 | SJCHGC02568 protein | 4.02 | 51,96 | 2 | - | - | - | NC |
| tr\|Q5D8H8 | SJCHGC01511 protein | 4.02 | 28,63 | 2 | - | - | - | - |
| tr\|C7TTM2 | Egg protein CP422 | 4.02 | 75 | 2 | - | - | - | SP |
| tr\|Q5C280 | SJCHGC07297 protein (Fragment) | 4.01 | 24,88 | 2 | - | - | - | - |
| tr\|C1LGQ4 | Proteasome 26S subunit subunit 4 ATPase | 4.01 | 21,78 | 2 | - | - | - | NC |
| tr\|A0A183RZ42 | Uncharacterized protein | 4.01 | 35,26 | 2 | - | - | - | - |
| tr\|A0A183PGL5 | MICOS complex subunit MIC60 | 4.01 | 29,64 | 2 | - | - | - | - |
| tr\|G4VAJ9 | 40S ribosomal protein S12 | 4.01 | 41,22 | 2 | - | - | - | NC |
| tr\|C1LE91 | Eukaryotic translation initiation factor 2A | 4.01 | 9,000 | 2 | 4.03 | 13,67 | 2 | - |
| tr\|C1L818 | KH domain-containing, RNA-binding, signal transduction-associated protein 1 | 4.01 | 38,89 | 2 | - | - | - | NC |
| tr\|A0A183MG44 | Innexin | 4 | 19,14 | 2 | - | - | - | NC |
| tr\|C1L7P6 | T-complex protein 1 subunit beta | 4 | 33,93 | 2 | - | - | - | - |
| tr\|Q5BW74 | SJCHGC04006 protein (Fragment) | 4 | 21,40 | 2 | 4 | 21,40 | 2 | - |
| tr\|Q5BY46 | SJCHGC08023 protein (Fragment) | 4 | 33,16 | 2 | - | - | - | NC |
| tr\|C9W185 | Putative uncharacterized protein | 4 | 37,92 | 2 | 4 | 3.862 | 2 | NC |
| tr\|C1LJK9 | Tetraspanin | 4 | 14,29 | 2 | - | - | - | SP |
| tr\|Q5DEU4 | SJCHGC08395 protein | 3.96 | 29,19 | 2 | 4 | 17,38 | 2 | NC |
| tr\|Q5C2S9 | SJCHGC03466 protein (Fragment) | 3.95 | 19,76 | 2 | - | - | - | SP |
| tr\|Q5C5A6 | SJCHGC04759 protein (Fragment) | 3.95 | 49,16 | 2 | - | - | - | - |
| tr\|G4V8L2 | Putative uncharacterized protein | 3.95 | 20,73 | 2 | - | - | - | - |
| tr\|C1LPL5 | Putative Stress-induced-phosphoprotein 1 | 3.93 | 44,51 | 2 | - | - | - | - |
| tr\|C1LJM7 | 26S proteasome regulatory subunit N12 | 3.93 | 32,08 | 2 | - | - | - | NC |
| tr\|Q5BSC7 | MICOS complex subunit MIC10 | 3.92 | 32,96 | 2 | - | - | - | - |
| tr\|Q5DHT7 | SJCHGC02803 protein | 3.87 | 40,47 | 2 | - | - | - | SP |
| tr\|Q5DG56 | SJCHGC03962 protein | 3.85 | 18,58 | 2 | - | - | - | NC |
| tr\|A0A094ZHC9 | Putative G-protein coupled receptor 179 | 3.82 | 21,08 | 2 | - | - | - | SP |
| tr\|C1LQ32 | Putative small nuclear ribonucleoprotein Sm D2 | 3.81 | 59,68 | 2 | - | - | - | NC |
| tr\|Q5DCY0 | SJCHGC02280 protein | 3.8 | 5,578 | 2 | - | - | - | SP |
| tr\|C1L3Q0 | Glutathione synthetase | 3.68 | 16,67 | 2 | - | - | - | - |
| tr\|G4M201 | Stromal cell-derived factor 2-like protein | 3.67 | 29,67 | 2 | - | - | - | SP |
| tr\|Q5DFL7 | Putative uncharacterized protein | 3.66 | 23,87 | 2 | 3.25 | 15,32 | 2 | NC |
| tr\|G4LV54 | Fer-1-related | 3.61 | 7,707 | 2 | - | - | - | - |
| tr\|Q5BXT8 | SJCHGC08926 protein (Fragment) | 3.57 | 24,79 | 2 | 3.82 | 24,79 | 2 | NC |
| tr\|C1LQW5 | Small ubiquitin-related modifier | 3.56 | 44,44 | 2 | - | - | - | NC |
| tr\|A0A183MV06 | Uncharacterized protein | 3.55 | 19,85 | 2 | 3.22 | 17,44 | 2 | - |
| tr\|C1LHA6 | Estradiol 17-beta-dehydrogenase 12-B | 3.54 | 13,68 | 2 | - | - | - | SP |
| tr\|Q5C689 | SJCHGC03832 protein (Fragment) | 3.5 | 35,06 | 2 | - | - | - | NC |
| tr\|G4V6B6 | Putative chromosome transmission fidelity factor | 3.49 | 10,53 | 2 | - | - | - | - |
| tr\|Q86EJ1 | Clone ZZD323 mRNA sequence | 3.48 | 27,59 | 2 | - | - | - | SP |
| tr\|Q5DB20 | SJCHGC04614 protein | 3.48 | 16,92 | 2 | - | - | - | SP |
| tr\|Q5DBV3 | Putative uncharacterized protein | 3.4 | 32,58 | 2 | - | - | - | - |
| tr\|Q5DAQ3 | Ribosomal Protein, Large subunit | 3.39 | 30,00 | 2 | - | - | - | NC |
| tr\|Q5DHV9 | SJCHGC09267 protein | 3.38 | 21,69 | 2 | - | - | - | NC |
| tr\|C1L4E5 | Actin related protein 2/3 complex, subunit 2 | 3.35 | 20,12 | 2 | - | - | - | NC |
| tr\|C1LIK2 | Eukaryotic translation initiation factor 3 subunit L | 3.32 | 14,86 | 2 | - | - | - | NC |
| tr\|G4VSL7 | Obg-like ATPase 1 | 3.24 | 7,828 | 2 | - | - | - | - |
| tr\|Q5BYJ0 | SJCHGC04275 protein (Fragment) | 3.23 | 26,87 | 2 | - | - | - | NC |
| tr\|C1L7V4 | Heat shock protein 67B2 | 3.22 | 36,07 | 2 | - | - | - | SP |
| tr\|A0A183PDA8 | Uncharacterized protein | 3.22 | 35,22 | 2 | - | - | - | NC |
| tr\|Q86EJ6 | Clone ZZD289 mRNA sequence | 3.21 | 22,75 | 2 | - | - | - | - |
| tr\|Q86DV1 | Clone ZZZ83 mRNA sequence | 3.21 | 39,03 | 2 | - | - | - | NC |
| tr\|Q5BY41 | SJCHGC06075 protein (Fragment) | 3.21 | 20,16 | 2 | - | - | - | NC |
| tr\|Q5C1E5 | SJCHGC06140 protein (Fragment) | 3.2 | 22,11 | 2 | - | - | - | - |
| tr\|C1LE56 | Malic enzyme | 3.17 | 26,55 | 2 | - | - | - | NC |
| tr\|Q5DFN3 | SJCHGC05602 protein | 3.14 | 42,57 | 2 | - | - | - | SP |
| tr\|Q5DFT5 | SJCHGC02033 protein | 3.12 | 38,85 | 2 | 3.55 | 23,99 | 2 | NC |
| tr\|Q5DC17 | SJCHGC05295 protein | 3.12 | 29,53 | 2 | - | - | - | - |
| tr\|Q5D8X1 | SJCHGC02842 protein | 3.11 | 27,41 | 2 | - | - | - | SP |
| tr\|C1LJT2 | Diazepam-binding inhibitor | 3.09 | 47,24 | 2 | 4 | 3.625 | 2 | NC |
| tr\|C1LRY1 | Ribosomal protein L17 | 3.07 | 33,16 | 2 | - | - | - | NC |
| tr\|A0A094ZZE3 | Basement membrane-specific heparan sulfate proteoglycan core protein | 3.07 | 26,62 | 2 | - | - | - | - |
| tr\|C7TXU2 | Regulator of chromosome condensation, RCC1,domain-containing protein | 3.06 | 12,98 | 2 | - | - | - | NC |
| tr\|Q5D981 | SJCHGC01869 protein | 3.04 | 17,13 | 2 | - | - | - | SP |
| tr\|Q5BVT6 | SJCHGC05037 protein (Fragment) | 2.97 | 21,61 | 2 | - | - | - | NC |
| tr\|Q86F64 | S-phase kinase-associated protein 1A | 2.95 | 41,10 | 2 | - | - | - | NC |
| tr\|A0A095C0B6 | Uncharacterized protein | 2.93 | 12,18 | 2 | - | - | - | SP |
| tr\|A0A183NYC3 | Uncharacterized protein | 2.91 | 9,465 | 2 | - | - | - | - |
| tr\|C1LDT7 | Placenta-specific gene 8 protein | 2.91 | 23,92 | 2 | - | - | - | NC |
| tr\|C1L466 | Chitinase domain-containing protein 1 | 2.9 | 26,28 | 2 | 3.7 | 24,23 | 2 | SP |
| tr\|Q5BZ09 | SJCHGC05847 protein (Fragment) | 2.89 | 22,66 | 2 | - | - | - | - |
| tr\|Q5DCA1 | SJCHGC07336 protein | 2.83 | 33,79 | 2 | 4.13 | 30,34 | 2 | - |
| tr\|Q5DHS9 | SJCHGC06793 protein | 2.78 | 33,18 | 2 | 3.32 | 33,18 | 2 | SP |
| tr\|C1LEQ8 | NADH dehydrogenase (Ubiquinone) Fe-S protein 1 | 2.72 | 18,28 | 2 | - | - | - | - |
| tr\|C1L985 | Eukaryotic translation elongation factor 1 beta 2 | 2.71 | 58,79 | 2 | - | - | - | - |
| tr\|G4V8C5 | Putative alpha-glucosidase | 2.71 | 19,15 | 2 | - | - | - | NC |
| tr\|Q5BWY6 | SJCHGC06832 protein (Fragment) | 2.7 | 8,696 | 2 | - | - | - | SP |
| tr\|Q5DEL6 | SJCHGC01506 protein | 2.59 | 20,50 | 2 | - | - | - | NC |
| tr\|A0A183MVN2 | Uncharacterized protein | 2.59 | 21,17 | 2 | - | - | - | NC |
| tr\|C1LEW7 | Aldehyde dehydrogenase 18 family, member A1 | 2.58 | 18,97 | 2 | - | - | - | NC |
| tr\|C1LNK4 | Egg protein CP1531 | 2.58 | 23,37 | 2 | - | - | - | SP |
| tr\|Q5DAJ0 | Calcium-regulated heat stable protein 1 (Calcium-regulated heat-stable protein of 24 kDa) | 2.57 | 36,75 | 2 | - | - | - | NC |
| tr\|C7TZP6 | Suppressor of profilin 2 (Fragment) | 2.56 | 31,65 | 2 | - | - | - | NC |
| tr\|C1LH00 | Uncharacterized protein | 2.55 | 28,27 | 2 | 2.94 | 28,27 | 2 | SP |
| tr\|Q5BX84 | SJCHGC02073 protein (Fragment) | 2.39 | 32,31 | 2 | 3.28 | 29,92 | 2 | NC |
| tr\|Q5BSQ1 | SJCHGC03596 protein | 2.36 | 53,25 | 2 | - | - | - | NC |
| tr\|Q5DBW9 | RAB family | 2.34 | 68,47 | 2 | - | - | - | NC |
| tr\|A0A183NUQ7 | Uncharacterized protein | 2.32 | 15,50 | 2 | - | - | - | - |
| tr\|Q5DG11 | Putative uncharacterized protein | 2.31 | 60,96 | 2 | 2.59 | 5.4 | 2 | SP |
| tr\|Q5BTN1 | Elongation factor-2 (Fragment) | 2.27 | 79,22 | 2 | - | - | - | NC |
| tr\|C7TZS5 | Lysyl-tRNA synthetase (Fragment) | 2.26 | 17,53 | 2 | - | - | - | NC |
| tr\|Q5DBX5 | SJCHGC06817 protein | 2.25 | 32,37 | 2 | 2.41 | 33,32 | 2 | SP |
| tr\|Q5DCH3 | Ubiquitin carboxyl-terminal hydrolase | 2.25 | 16,67 | 2 | - | - | - | NC |
| tr\|Q5BR36 | SJCHGC09702 protein (Fragment) | 2.17 | 43,77 | 2 | - | - | - | NC |
| tr\|G4VHS0 | Putative 40s ribosomal protein S14 | 2.14 | 51,31 | 2 | - | - | - | - |
| tr\|C1L3P2 | DNA-directed RNA polymerase II subunit C | 2.08 | 28,56 | 2 | - | - | - | NC |
| tr\|Q5BRB5 | SJCHGC08770 protein (Fragment) | 2.07 | 20,99 | 2 | - | - | - | NC |
| tr\|Q5C2U1 | SJCHGC08243 protein (Fragment) | 2.01 | 28,31 | 2 | - | - | - | NC |
| tr\|G4V7X9 | Adducin related protein | 2.01 | 22,21 | 2 | - | - | - | - |
| tr\|A0A183PGJ8 | Uncharacterized protein | 2.01 | 21,18 | 2 | - | - | - | NC |
| tr\|G4V8P1 | Putative tektin | 2.01 | 33,07 | 2 | - | - | - | NC |
| tr\|Q5DE01 | Ribosomal protein S15 | 2 | 16,55 | 2 | - | - | - | - |
| tr\|Q86FJ4 | SJCHGC06654 protein (Fragment) | 2 | 21,98 | 2 | 2 | 21,98 | 2 | SP |
| tr\|Q86EY7 | SJCHGC09497 protein | 2 | 37,04 | 2 | - | - | - | - |
| tr\|G4VBC5 | Cathepsin F (C01 family) | 2 | 20,38 | 2 | - | - | - | SP |
| tr\|Q5DB43 | SJCHGC02877 protein | 1.87 | 43,86 | 2 | - | - | - | NC |
| tr\|A0A183MTC5 | Uncharacterized protein | 1.87 | 18,27 | 2 | - | - | - | - |
| tr\|C1LK33 | Glycine cleavage system H protein | 1.82 | 31,58 | 2 | - | - | - | - |
| tr\|Q5DEW2 | SJCHGC01637 protein | 1.8 | 49,77 | 2 | - | - | - | NC |
| tr\|Q5DHX0 | SJCHGC06263 protein | 1.77 | 38,08 | 2 | - | - | - | - |
| tr\|C1LJT8 | ATP-dependent Clp protease proteolytic subunit | 1.76 | 23,28 | 2 | - | - | - | - |
| tr\|O02034 | Amidase | 1.75 | 28,65 | 2 | - | - | - | NC |
| tr\|Q5DCQ8 | Myosin alkali light chain 1 | 1.68 | 49,36 | 2 | - | - | - | - |
| tr\|Q5DAI5 | SJCHGC09312 protein | 1.68 | 17,81 | 2 | - | - | - | NC |
| tr\|Q5BYW1 | SJCHGC05984 protein (Fragment) | 1.67 | 16,84 | 2 | - | - | - | NC |
| tr\|Q5DHF7 | Serine/threonine-protein phosphatase 2A activator | 1.56 | 25,06 | 2 | 2.48 | 22,58 | 2 | NC |
| tr\|G4V9N7 | Egf-like domain protein | 1.47 | 12,04 | 2 | 3.11 | 10,18 | 3 | SP |
| tr\|Q5DFH8 | SJCHGC06606 protein | 1.36 | 26,03 | 2 | 3.84 | 26,03 | 2 | NC |
| tr\|A0A095AYR0 | Alpha-mannosidase | 1.29 | 10,36 | 2 | - | - | - | SP |
| tr\|C1L3W0 | Myosin-7 | 1.14 | 40,27 | 2 | - | - | - | - |
| tr\|C1LGV1 | Alpha-glucosidase | 1.09 | 16,96 | 2 | - | - | - | NC |
| tr\|Q5C6U4 | SJCHGC04301 protein (Fragment) | 0.8 | 7,553 | 2 | - | - | - | NC |
| tr\|Q5BRV6 | SJCHGC07249 protein (Fragment) | 0.67 | 25 | 2 | - | - | - | NC |
| tr\|B3W649 | Putative uncharacterized protein (Fragment) | 0.66 | 34,27 | 2 | 1.3 | 34,27 | 2 | NC |
| tr\|C1LE34 | GLIPR1-like protein 1 | 0.59 | 25,49 | 2 | 2.75 | 25,49 | 2 | NC |
| tr\|Q5BSU9 | MICOS complex subunit MIC60 | 0.54 | 31,86 | 2 | - | - | - | NC |
| tr\|C1LKW0 | Uncharacterized protein | 0.53 | 46,18 | 2 | 0.81 | 24,86 | 2 | SP |
| tr\|Q5BRK1 | SJCHGC08068 protein (Fragment) | 0.52 | 37,83 | 2 | - | - | - | NC |
| tr\|C1LNE9 | Hypotherical protein | 0.5 | 33,05 | 2 | - | - | - | - |
| tr\|C1L849 | Putative Cofilin-1 | 0.49 | 45,19 | 2 | - | - | - | - |
| tr\|G4VMW9 | Putative vesicular-fusion protein nsf | 0.46 | 21,67 | 2 | 0.77 | 22,60 | 2 | - |
| tr\|C1LJ79 | RAS-like GTP-binding protein | 0.32 | 28,09 | 2 | - | - | - | NC |
| tr\|C1LXE0 | Putative uncharacterized protein | 0.16 | 31,77 | 2 | - | - | - | SP |
| tr\|G4V7R9 | Dolichyl-diphosphooligosaccharide--protein glycosyltransferase 48 kDa subunit | 0.13 | 18,06 | 2 | - | - | - | SP |
| tr\|Q5DEQ5 | SJCHGC09276 protein | 0.12 | 18,50 | 2 | 1.14 | 18,50 | 3 | - |
| tr\|A0A183QEQ9 | Uncharacterized protein | 0.08 | 8,409 | 2 | - | - | - | - |
| tr\|A0A183PPD7 | Uncharacterized protein | 0.06 | 20,93 | 2 | - | - | - | NC |
| tr\|G4LVD1 | Putative uncharacterized protein | 0.05 | 20,93 | 2 | - | - | - | NC |
| tr\|G4LY02 | NIF3-like protein 1 | 0.05 | 23,81 | 2 | - | - | - | - |
| ESP: Egg Secretory Proteins. Unused: Quantification for proteins in the ProteinPilot software. %Cov: ratio of the protein sequence covered by the matched peptides. Peptides (95%): total number of detected peptides with 95% of confidence. SecP (Secretome P 2.0) results described as: SP, indicate presence of predicted signal sequence; NC, indicate non-classical secreted proteins; ( - ), non-detected. Dark color highlights protein identified both in eggs and ESP. White lines are proteins exclusively found in the eggs. | | | | | | | | |
