# Supplementary material for: Qualitative and quantitative proteomic analyses of Schistosoma japonicum eggs and egg-derived secretory-excretory proteins
Source: Parasit Vectors. 2019 Apr 16;12:173. doi: 10.1186/s13071-019-3403-1 (PMC6469072; doi:10.1186/s13071-019-3403-1)
Supplement: Supplementary file 5 — Additional file 5: Table S2. Proteins identified with significant variation in S. japonicum immature eggs compared with mature eggs, listed in descending order of variation. [file 13071_2019_3403_MOESM5_ESM.docx]

| **Supplementary Table 2.** Proteins identified with significant variation in *S. japonicum* immature eggs compared with mature eggs, listed in descending order of variation. | | | | | | | |
| --- | --- | --- | --- | --- | --- | --- | --- |
| **Molecular function** | **Accession** | **Protein name** | **Unused** | **%Cov** | **Peptides (95%)** | **SecP** | **Variation(±SD)** |
| Catalytic activity | tr\|G4M201 | Stromal cell-derived factor 2-like protein | 3,67 | 29,67 | 2 | SP | 266(±84) |
| n/a | tr\|Q5DA91 | Receptor expression-enhancing protein | 12,89 | 35,98 | 9 | NC | 143(±44) |
| Binding | tr\|Q5DH23 | SJCHGC09129 protein | 22 | 80,83 | 196 | - | 108(±22,5) |
| Catalytic activity | tr\|Q5DA69 | SJCHGC01759 protein | 30,79 | 94,09 | 57 | - | 38,3(±17,9) |
| Catalytic activity | tr\|O45033 | Calpain (Fragment) | 13,42 | 35,78 | 8 | - | 20,9(±8,5) |
| Binding | tr\|G4V9U8 | Putative uncharacterized protein | - | - | - | - | 18,1(±5,85) |
| Catalytic activity | tr\|Q86DY0 | Putative tyrosine 3-monooxygenase | 36,58 | 75,19 | 28 | - | 15,9(±7,85) |
| Binding | tr\|Q5DAJ0 | Calcium-regulated heat stable protein 1 (Calcium-regulated heat-stable protein of 24 kDa) | 2,57 | 36,75 | 2 | NC | 15,6(±3,35) |
| Binding | tr\|Q86EI5 | Clone ZZD336 mRNA sequence | 6,42 | 70,56 | 5 | - | 13,4(±0,4) |
| Catalytic activity | tr\|A0A183LMH8 | Uncharacterized protein | 4,17 | 27,03 | 3 | - | 11,5(±2,3) |
| n/a | tr\|C1LNT1 | Estrogen-regulated protein EP45 | 16,65 | 34,40 | 9 | NC | 11,3(±2,45) |
| Catalytic activity | tr\|C1LF96 | Ethanolamine kinase 1 | 5,61 | 27,41 | 3 | - | 10,9(±1) |
| n/a | tr\|Q5D8S6 | SJCHGC03291 protein | 9,59 | 49,07 | 5 | SP | 10,3(±4,75) |
| n/a | tr\|Q5D925 | SJCHGC09142 protein | 10,6 | 76,08 | 15 | - | 10,2(±3,9) |
| Binding | tr\|Q5DG56 | SJCHGC03962 protein | 3,85 | 18,58 | 2 | NC | 10,2(±2,1) |
| Catalytic activity | tr\|Q5DHC0 | Proteasome subunit beta type | 27,21 | 76,88 | 22 | - | 10,1(±1,45) |
| Catalytic activity | tr\|C1L4W2 | Guanylate kinase | 8 | 24,87 | 5 | NC | 9,43(±2,2) |
| Transporter activity | tr\|C1LUF5 | ATP:ADP antiporter | 4,14 | 42,77 | 3 | - | 8,75(±0,9) |
| Catalytic activity | tr\|Q27776 | Glyceraldehyde-3-phosphate dehydrogenase | 70,79 | 94,66 | 83 | - | 8,49(±2,3) |
| Binding | tr\|A0A183QAA5 | Uncharacterized protein | 2,09 | 43,09 | 5 | - | 7,53(±1,65) |
| Catalytic activity | tr\|C1LIX2 | Putative Thioredoxin, mitochondrial | 8,3 | 80,54 | 7 | NC | 7,44(±0,95) |
| n/a | tr\|C1LA91 | Translationally-controlled tumor protein homolog | 5,98 | 46,75 | 4 | NC | 7,1(±2,35) |
| n/a | tr\|Q5DDQ4 | SJCHGC06119 protein |  |  |  | NC | 7,1(±1,55) |
| Binding | tr\|C1LVE4 | Tubulin beta chain | 8 | 91,80 | 99 | NC | 6,06(±1,25) |
| n/a | tr\|Q5DHT7 | SJCHGC02803 protein | 3,87 | 40,47 | 2 | SP | 6,01(±2,75) |
| Binding | tr\|Q5DDA0 | Adenylyl cyclase-associated protein | 6,76 | 31,92 | 5 | - | 5,9(±1,05) |
| Binding | tr\|Q5DBM0 | Annexin | 5,98 | 24,28 | 3 | - | 5,73(±1,25) |
| n/a | tr\|Q5BQQ3 | Putative dynein light chain | - | - | - | - | 5,55(±0,15) |
| Catalytic activity | tr\|C1L730 | Peptidyl-prolyl cis-trans isomerase | 18,02 | 72,04 | 19 | SP | 5,07(±1,3) |
| n/a | tr\|Q5D8P1 | SJCHGC06542 protein | 9,97 | 72,78 | 11 | NC | 4,81(±0,65) |
| Binding | tr\|Q5DEW6 | SJCHGC09345 protein | 17,96 | 34,72 | 9 | SP | 4,5(±0,95) |
| Binding | tr\|C1L8V7 | Proliferating cell nuclear antigen | 34,01 | 71,92 | 20 | NC | 4,27(±0,6) |
| n/a | tr\|C1LFB1 | Coatomer subunit delta | 6,87 | 27,30 | 5 | - | 4,14(±0,9) |
| Enzyme regulator activity | tr\|C1LIQ6 | Ubiquitin-activating enzyme E1 | 8,1 | 21,82 | 5 | NC | 4,11(±1,05) |
| n/a | tr\|A0A095BVQ5 | Uncharacterized protein | - | - | - | SP | 3,87(±0,2) |
| Catalytic activity | tr\|Q5DFZ8 | Fructose-bisphosphate aldolase | 91,95 | 96,42 | 136 | - | 3,86(±0,6) |
| Catalytic activity | tr\|Q5DBE6 | ADP-ribose pyrophosphatase, mitochondrial | 17,13 | 93,40 | 16 | NC | 3,8(±1,05) |
| n/a | tr\|C1LCT0 | Phosphatase 2A inhibitor I2PP2A | 6,02 | 47,20 | 5 | NC | 3,79(±0,2) |
| Binding | tr\|Q5DFR7 | SJCHGC06291 protein | 32,7 | 82,40 | 36 | NC | 3,55(±1,2) |
| Catalytic activity | tr\|Q5D9T6 | S-methyl-5'-thioadenosine phosphorylase | 61,18 | 94,65 | 79 | - | 3,5(±0,25) |
| n/a | tr\|Q5DCY0 | SJCHGC02280 protein | 3,8 | 5,578 | 2 | SP | 3,44(±0,15) |
| Binding | tr\|C1LJT2 | Diazepam-binding inhibitor | 3,09 | 47,24 | 2 | NC | 3,27(±0,45) |
| n/a | tr\|A0A183N2T0 | Uncharacterized protein | 26,39 | 63,51 | 21 | - | 3,2(±0,9) |
| n/a | tr\|A0A183R3C1 | Uncharacterized protein | 3,38 | 41,17 | 28 | - | 3,07(±0,7) |
| Binding | tr\|C1LMR7 | Putative Cofilin-1 | 5,04 | 46,66 | 3 | - | 3,04(±0,8) |
| Catalytic activity | tr\|Q5DBC8 | Aspartate aminotransferase | 26,4 | 65,23 | 26 | NC | 3,03(±0,5) |
| Catalytic activity | tr\|C1L860 | L-lactate dehydrogenase | 47,65 | 89,73 | 41 | NC | 3,01(±0,15) |
| Enzyme regulator activity | tr\|F0UXG3 | Cystatin-B (Stefin-B) | 4,72 | 76,23 | 4 | - | 2,85(±1,15) |
| Structural molecule activity | tr\|G4VGB4 | Putative coatomer beta subunit | 8 | 26,37 | 5 | - | 2,76(±1,15) |
| Catalytic activity | tr\|Q5DHV9 | SJCHGC09267 protein | 3,38 | 21,69 | 2 | NC | 2,74(±0,35) |
| Binding | tr\|C1L8C8 | Small subunit ribosomal protein S2e | 8,05 | 59,61 | 4 | NC | 2,73(±1,3) |
| Catalytic activity | tr\|Q5DAL5 | Serine/threonine-protein phosphatase (Fragment) | 4 | 53,07 | 7 | NC | 2,68(±1,05) |
| Catalytic activity | tr\|Q5BZG5 | SJCHGC05582 protein (Fragment) | 12,65 | 39,57 | 7 | - | 2,67(±0,95) |
| Binding | tr\|C4Q5U3 | Ankyrin 2,3/unc44, putative (Fragment) | 10,3 | 15,98 | 5 | NC | 2,62(±0,3) |
| Binding | tr\|A0A183KH93 | Uncharacterized protein | 4,01 | 92,01 | 211 | - | 2,58(±1) |
| Catalytic activity | tr\|Q5DBQ9 | SJCHGC01755 protein | 20,18 | 71,71 | 24 | - | 2,58(±0,9) |
| Binding | tr\|Q5C1F3 | SJCHGC04997 protein (Fragment) | 41,04 | 79,36 | 29 | - | 2,53(±1,15) |
| Binding | tr\|Q86ES4 | Actin depolymerizing factor-like protein | 16,26 | 80,58 | 9 | NC | 2,48(±0,65) |
| Catalytic activity | tr\|Q5DGI7 | SJCHGC02838 protein | 18,69 | 51,08 | 16 | SP | 2,45(±1,1) |
| Binding | tr\|C7TXR1 | Lymphocyte cytosolic protein 1 | 29,27 | 66,86 | 20 | - | 2,45(±0,6) |
| Catalytic activity | tr\|Q5DCQ5 | SJCHGC06657 protein | 5,98 | 38,96 | 3 | - | 2,41(±0,65) |
| Catalytic activity | tr\|Q5DD05 | SJCHGC05876 protein | 12,19 | 33,12 | 8 | NC | 2,35(±1) |
| Catalytic activity | tr\|Q86ER2 | Clone ZZD1514 mRNA sequence | 7,34 | 50 | 5 | NC | 2,35(±0,75) |
| Catalytic activity | tr\|Q9U8F3 | Thioredoxin | 19,63 | 83,02 | 34 | NC | 2,35(±0,65) |
| n/a | tr\|Q7Z0T6 | Adenosinetriphosphatase | 3,63 | 62,91 | 7 | - | 2,31(±1,1) |
| Antioxidant activity | tr\|Q5DEY1 | SJCHGC00794 protein | 44,91 | 74,33 | 63 | SP | 2,28(±0,15) |
| Catalytic activity | tr\|C1LFP4 | Putative aldehyde dehydrogenase 1B1 | 32,65 | 75,76 | 22 | NC | 2,2(±0,5) |
| Binding | tr\|A0A183RCJ8 | Uncharacterized protein | 2,03 | 30,84 | 11 | SP | 2,15(±0,65) |
| n/a | tr\|Q5C3C2 | SJCHGC05634 protein (Fragment) | 4,15 | 21,00 | 3 | NC | 2,12(±0,45) |
| n/a | tr\|C1LQM8 | Uncharacterized protein | 19,82 | 64,09 | 11 | SP | 2,11(±0,1) |
| n/a | tr\|Q5C5L3 | SJCHGC06471 protein (Fragment) | 7,77 | 55,79 | 4 | NC | 2,1(±0,15) |
| Catalytic activity | tr\|C1LRF1 | Aldo-keto reductase family 1, member B4 (Aldose reductase) | 51,02 | 78,71 | 43 | - | 2,04(±0,25) |
| Catalytic activity | tr\|Q5DFU2 | NADH dehydrogenase (Ubiquinone) 1 beta subcomplex 10 | 9,94 | 49,52 | 6 | - | 2,03(±0,8) |
| Catalytic activity | tr\|C1LLG3 | Histidine triad nucleotide binding protein 1 | 10,01 | 60,90 | 8 | NC | 2,02(±0,55) |
| Catalytic activity | tr\|C1LRH4 | Calcium/calmodulin-dependent serine protein kinase (MAGUK family) | 1,45 | 31,20 | 9 | SP | -2,01(±0,05) |
| n/a | tr\|C1LIX6 | Loss of heterozygosity 11 chromosomal region 2 gene A protein homolog | 43,14 | 50,84 | 32 | NC | -2,2(±0,45) |
| Binding | tr\|G4LZ30 | Calcium-binding protein, putative | - | - | - | NC | -2,2(±0,85) |
| n/a | tr\|C1LQJ2 | Uncharacterized protein | 30,97 | 60,39 | 53 | SP | -2,26(±0,4) |
| Electron carrier activity | tr\|Q86EU4 | Cytochrome c proximal | 11,21 | 85,18 | 6 | - | -2,31(±1) |
| n/a | tr\|Q5D981 | SJCHGC01869 protein | 3,04 | 17,13 | 2 | SP | -2,32(±0,45) |
| Catalytic activity | tr\|C1LK99 | Purine nucleoside phosphorylase | 55,04 | 96,85 | 96 | NC | -2,61(±0,4) |
| Catalytic activity | tr\|C1L5C5 | Putative aminopeptidase | 62,75 | 85,40 | 45 | NC | -2,69(±0,2) |
| n/a | tr\|Q86FB2 | SJCHGC05154 protein | - | - | - | SP | -2,89(±0,85) |
| Binding | tr\|C7TZX9 | Ribonuclease Oy (Fragment) | 7,52 | 21,43 | 5 | SP | -2,93(±0,95) |
| n/a | tr\|Q5BQX6 | SJCHGC09770 protein | 9,48 | 58,74 | 6 | NC | -3,41(±0,3) |
| Catalytic activity | tr\|C1LGJ8 | Transmembrane serine protease 8 | - | - | - | SP | -3,44(±0,95) |
| Binding | tr\|A0A183QVR5 | Histone H4 | 38,5 | 89,13 | 45 | - | -3,52(±0,5) |
| n/a | tr\|Q5DHY8 | Epididymal secretory protein E1 (Niemann Pick type C2 protein homolog) | 6,47 | 56,16 | 5 | SP | -3,63(±1,25) |
| Structural molecule activity | tr\|Q5DDX5 | Ribosomal protein L18 | 7,93 | 48,93 | 4 | - | -3,8(±0,5) |
| Binding | tr\|C1LEH2 | Ribonuclease Oy | 7,61 | 29,75 | 7 | SP | -3,88(±0,3) |
| Receptor activity | tr\|G4M0F6 | Macrophage scavenger receptor-related | 4,79 | 11,86 | 3 | NC | -3,97(±0,9) |
| n/a | tr\|C1L670 | Tektin-4 | 10,82 | 44,60 | 5 | NC | -3,99(±0,92) |
| Catalytic activity | tr\|C1LGF1 | Phosphorylase kinase, alpha 2 | 10,55 | 64,19 | 6 | - | -4,02(±1,05) |
| n/a | tr\|C1LDX0 | Hypotheticial protein | 4 | 12,72 | 3 | SP | -4,22(±0,8) |
| Catalytic activity | tr\|C1LGA4 | Glycogen [starch] synthase | - | - | - | NC | -4,22(±1,6) |
| Catalytic activity | tr\|C1LEW5 | Peptidase M8, leishmanolysin,domain-containing protein | 20,83 | 36,82 | 14 | - | -4,43(±0,7) |
| Catalytic activity | tr\|C7TYR0 | Proteasome subunit beta type | 19,87 | 71,21 | 18 | - | -5,26(±1,8) |
| Catalytic activity | tr\|C1LH45 | Fructose-1,6-bisphosphatase | 26,58 | 67,04 | 22 | NC | -5,45(±2,65) |
| Binding | tr\|A0A183MGF1 | Uncharacterized protein | 15,28 | 26,51 | 10 | - | -5,52(±2,75) |
| Catalytic activity | tr\|A0A094ZFT4 | Glycogen debranching enzyme (Fragment) | 9,9 | 22,41 | 8 | - | -6,36(±0,15) |
| n/a | tr\|A0A183RJG7 | Uncharacterized protein | 6 | 41,67 | 3 | NC | -6,43(±0,45) |
| n/a | tr\|C1LEY0 | Spermatogenesis-associated protein 6 | 6,75 | 31,67 | 4 | NC | -6,75(±2,05) |
| n/a | tr\|Q5DED2 | SJCHGC02251 protein | 4,16 | 21,25 | 2 | NC | -7,34(±3,2) |
| Catalytic activity | tr\|Q5C3M2 | SJCHGC03231 protein (Fragment) | 20,93 | 60,19 | 18 | - | -7,84(±0,65) |
| n/a | tr\|C7TTM2 | Egg protein CP422 | 4,02 | 75 | 2 | SP | -8,05(±1,7) |
| n/a | tr\|G4VN72 | Putative tropomyosin | 4,53 | 50,34 | 3 | NC | -8,5(±1,8) |
| Binding | tr\|Q5DF77 | SJCHGC01853 protein | 17,63 | 68,84 | 22 | - | -8,78(±3,7) |
| n/a | tr\|C1LUR6 | Peptidase inhibitor 16 | 15,02 | 52,38 | 11 | SP | -8,95(±2,4) |
| Binding | tr\|C1LYI9 | Calcium-binding EF-hand,domain-containing protein | 6,43 | 84,50 | 4 | NC | -9,3(±2) |
| Catalytic activity | tr\|C1L6Z8 | Mitochondrial processing peptidase | 8,39 | 27,30 | 6 | - | -9,74(±0,85) |
| Binding | tr\|C1LEZ4 | Tegumental protein | 13,99 | 59,21 | 12 | NC | -9,89(±2,1) |
| n/a | tr\|Q5D8R2 | SJCHGC08964 protein (Fragment) | 17,21 | 65,93 | 10 | - | -9,92(±0,9) |
| n/a | tr\|C1LFS0 | Tektin-2 | 15,53 | 45,89 | 9 | NC | -9,95(±3,9) |
| n/a | tr\|C7TZ90 | Putative uncharacterized protein | 6,19 | 28,94 | 8 | SP | -10,2(±2,4) |
| n/a | tr\|Q5BWI4 | SJCHGC04358 protein (Fragment) | 7,45 | 30,86 | 14 | NC | -10,6(±1,3) |
| Catalytic activity | tr\|Q5DEU4 | SJCHGC08395 protein | 3,96 | 29,19 | 2 | NC | -11,6(±1.10) |
| Catalytic activity | tr\|C1LP66 | Aquaporin-3 (AQP-3) | - | - | - | - | -14,4(±2.0) |
| n/a | tr\|C1LFL0 | Uncharacterized protein | 6,4 | 51,34 | 7 | SP | -15,9(±3,8) |
| Binding | tr\|Q86E42 | Annexin | 24,42 | 56,36 | 12 | NC | -16(±7,5) |
| n/a | tr\|C1L425 | Serpin B6 (Placental thrombin inhibitor) | 21,74 | 47,79 | 12 | - | -16,9(±4,4) |
| n/a | tr\|C1LS13 | Cell wall integrity and stress response component 1 | 19,24 | 56,25 | 20 | - | -31(±11,5) |
| n/a | tr\|Q5DI27 | SJCHGC06484 protein | 4,42 | 30,32 | 3 | SP | -47,6(±15,5) |
| n/a | tr\|Q5DFT5 | SJCHGC02033 protein | 3,12 | 38,85 | 2 | NC | -94,1(±1,8) |
| n/a | tr\|Q5DAC6 | SJCHGC05576 protein | 6 | 25,51 | 8 | SP | -103(±43,25) |
| Unused: Quantification for proteins in the ProteinPilot software. %Cov: ratio of the protein sequence covered by the matched peptides. Peptides (95%): total number of detected peptides with 95% of confidence. SecP (Secretome P 2.0) results described as: SP, indicate presence of predicted signal sequence; NC, indicate non-classical secreted proteins; ( - ), reported when proteins were not secretory proteins by the program prediction. Variation: average of time fold variation of a specific protein expression in *S. japonicum* immature eggs compared to its expression in mature eggs ± Standard deviation (SD). | | | | | | | |
